# *In vivo* oral insulin delivery *via* covalent organic frameworks†

**DOI:** 10.1039/d0sc05328g

**Published:** 2021-04-06

**Authors:** Farah Benyettou, Nawel Kaddour, Thirumurugan Prakasam, Gobinda Das, Sudhir Kumar Sharma, Sneha Ann Thomas, Fadia Bekhti-Sari, Jamie Whelan, Mohammed A. Alkhalifah, Mostafa Khair, Hassan Traboulsi, Renu Pasricha, Ramesh Jagannathan, Nassima Mokhtari-Soulimane, Felipe Gándara, Ali Trabolsi

**Affiliations:** New York University Abu Dhabi P.O. Box 129188 Abu Dhabi United Arab Emirates ali.trabolsi@nyu.edu; Laboratory of Physiology Physiopathology and Biochemistry of Nutrition, Department of Biology, University of Tlemcen Algeria; Department of Chemistry, College of Science, King Faisal University P.O. Box 400, Al-Ahsa 31982 Saudi Arabia; School of Chemistry, University of Bristol Cantocks Close Bristol BS8 1TS UK; Materials Science Institute of Madrid – CSIC Spain

## Abstract

With diabetes being the 7th leading cause of death worldwide, overcoming issues limiting the oral administration of insulin is of global significance. The development of imine-linked-covalent organic framework (nCOF) nanoparticles for oral insulin delivery to overcome these delivery barriers is herein reported. A gastro-resistant nCOF was prepared from layered nanosheets with insulin loaded between the nanosheet layers. The insulin-loaded nCOF exhibited insulin protection in digestive fluids *in vitro* as well as glucose-responsive release, and this hyperglycemia-induced release was confirmed *in vivo* in diabetic rats without noticeable toxic effects. This is strong evidence that nCOF-based oral insulin delivery systems could replace traditional subcutaneous injections easing insulin therapy.

## Abstract 2

We report the successful use of a gastro-resistant covalent organic framework for *in vivo* oral delivery of insulin.

## Graphical abstract

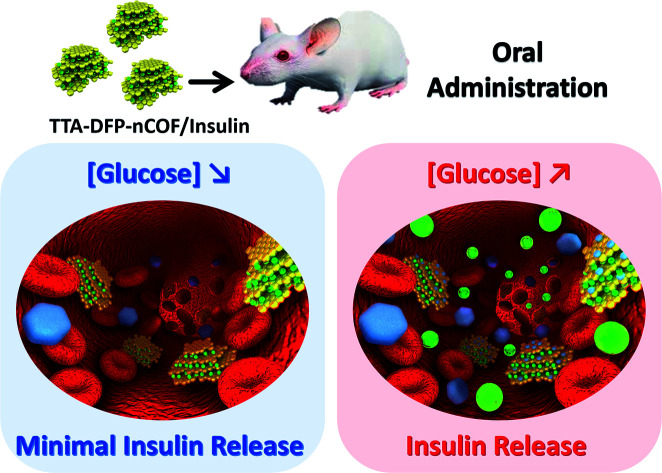

## Introduction

With diabetes being the 7th leading cause of death worldwide affecting nearly 10% of the world's population, with a quadrupling of its prevalence since 1980,^1^ and accounting for almost 15% of direct healthcare costs,^2^ its treatment is of global significance.^3^ Diabetes is a chronic disease occurring when no insulin is produced due to the absence of pancreatic β-cell islets (type 1)^4^ or the insulin that is produced is incapable of being effectively utilized by the body (type 2).^5^ Coupled with lifestyle changes, insulin therapy remains a key element in controlling and regulating blood glucose levels, with the primary mechanism being insulin injection. However, studies have shown delays in the onset of insulin therapy in a large proportion of people with uncontrolled diabetes, and in those who do eventually undertake treatment, there is a delay of more than 2 years.^6^ A fear of needles and self-injection^7^ as well as pain and anxiety^8^ are some of the many reasons why people are unwilling to start insulin therapy. Insulin pens alleviate some of these worries, as well as overcome dosage issues that exist with vials and syringes;^9^ however this method is itself not error free.^10^ A shift towards oral delivery of insulin has the potential to improve the uptake of insulin therapy and revolutionize diabetes care since it is a noninvasive therapeutic approach without the side effects caused by frequent subcutaneous (S.C.) injection.^11–14^

Orally delivered insulin is capable of reaching the systemic circulation after passing through the liver similar to physiologically secreted insulin, while subcutaneously injected insulin may result in peripheral hyperinsulinemia and associated complications.^15^ However, oral drug delivery faces numerous challenges including dissolution, bioavailability, solubility and its stability in the gastrointestinal (GI) tract.^16^ The oral bioavailability of insulin is severely hampered by its inherent instability in the GI tract and its low permeability across biological membranes in the intestine (less than 1%).^17^ Despite clinical trials of several oral insulin formulations,^18,19^ sufficient commercial development has not yet been achieved.^20^

To be considered an effective oral insulin delivery method, the proposed system must comprise a biocompatible, high-loading platform affording insulin protection against external acidic environments and enzymatic degradation, in addition to targeted drug delivery coupled with stimuli-responsive drug release such as hyperglycemia.^21^ Nanocarriers such as polymeric, inorganic and solid-lipid nanoparticles have emerged as effective insulin transporters, circumventing many of the problems associated with oral insulin delivery, and show promise for desirable biopharmaceutical and pharmacokinetic properties.^22–24^ However, recent clinical trials have resulted in failure due to toxicology, low levels of oral bioavailability and elevated intra-individual differences in insulin absorption, thereby offering strong evidence that challenges still persist.^25–28^ Two systems have, so far, been FDA approved for the oral delivery of insulin.^29,30^ The first one, developed by Oramed (ORMD-0801), incorporates both a species-specific protease inhibitor that protects active ingredients and a potent absorption enhancer that fosters their absorption across the intestinal epithelium. However, the system is non-specific, and its prolonged use may damage the stomach membrane and may lead to toxicity.^29,30^ The second, HDV-I by Diasome, is based on liposomes with hepatic targeting and suffers from instability in the GI tract, high cost and drug release during storage.^29,30^

Nanoparticles offer better storage and physiological stability compared to other nanosized colloidal carriers such as liposomes and emulsions,^27^ with nanoscale imine-linked covalent organic frameworks (nCOFs)^31^ in particular having shown tremendous potential as emerging nanomedicine candidates for drug delivery.^32–41^ nCOFs feature a long-range ordered structure in which the organic building blocks are spatially controlled in two or three dimensions leading to regular pores with diameters facilitating the loading and controlled release of large drugs and proteins/enzymes (Table S1†).^39,42,43^ In addition, their high flexibility in molecular architecture and functional design make them versatile and therefore give them unique responsivity to their environment.

Herein, our previously described^44^ imine-based nCOF obtained from the co-condensation of 2,6-diformylpyridine (DFP) and 4,4′,4′′-(1,3,5-triazine-2,4,6-triyl)trianiline (TTA) (denoted as TTA-DFP-nCOF) was prepared from highly crystalline nanoparticles using a seeded growth method^45,46^ and was successfully used as an oral insulin delivery system. The choice of the triazine-based TTA-DFP-nCOF was primarily based on its high stability under harsh conditions including acidic environments.^47,48^ The unique features of this delivery method are its high insulin-loading capacity (∼65 wt%), biocompatibility, insulin protection under harsh conditions and hyperglycemia-induced drug release. The insulin-loaded TTA-DFP-nCOF successfully crossed the intestinal barrier and sustainably reduced the blood glucose level *in vivo* in type 1 diabetic (T1D) rat model with the glucose level completely returning to normal as compared to the non-diabetic control group without inducing systemic toxicity. In comparison to the two FDA-approved technologies, our system is biocompatible, highly stable in the stomach, cost effective, specific and glucose-responsive, representing a step forward in the future of oral insulin delivery and a novel pathway toward the treatment of type 1 through nCOF-based oral insulin delivery.

## Results and discussion

**Fig. 1:**
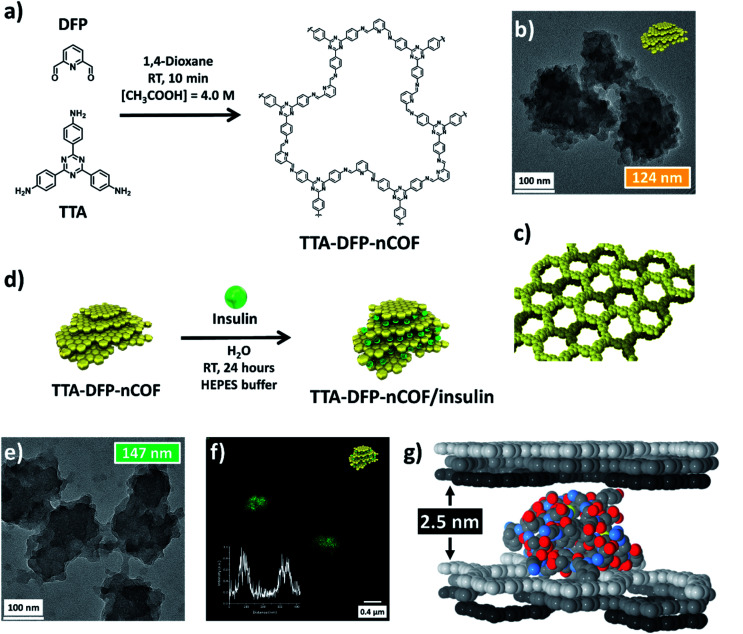
Insulin is intercalated between TTA-DFP-nCOF layers. (a) Chemical structure and synthesis of the TTA-DFP-nCOF. (b) HR-TEM image of the TTA-DFP-nCOF. Cartoon representation (yellow nanoparticles) of the shape of the TTA-DFP-nCOF. (c) Structural model of the TTA-DFP-nCOF, consisting of hcb layers that are disposed in the abc sequence, generating hexagonal channels along the stacking direction. (d) Schematic representation of the encapsulation of insulin between the layers of the TTA-DFP-nCOF. Cartoon representation (green spheres) represents the insulin. (e) HR-TEM image of TTA-DFP-nCOF/insulin. (f) Confocal microscopy image of TTA-DFP-nCOF/insulin-FITC; inset: fluorescence intensity. (g) van der Waals representation of the optimized location of the insulin monomer molecule intercalated between TTA-DFP-nCOF layers. Atoms belonging to the COF layers are displayed in white, grey, and black color, for each individual layer. For the insulin molecule, the C, N, O, and S atoms are grey, blue, red, and yellow, respectively. H atoms are omitted for clarity.

The TTA-DFP-nCOF was synthesized by co-condensation of DFP (21 mg, 0.15 mmol, 5 equivalents) and TTA (12 mg, 0.03 mmol, 1 equivalent), in anhydrous 1,4-dioxane (3 mL) in the presence of 0.5 mL of 13 M acetic acid ([acetic acid]_final_ = 4.0 M) at room temperature for 10 min (Fig. 1a). The solution was cleaned using dialysis in H_2_O to obtain a stable nanoparticle suspension. After 10 min at room temperature, imine-linked covalent organic nanoparticles with an average diameter of 123.7 nm (Fig. 1b and S2†) emerge from the clear solution without forming amorphous polyimine precipitates. A high concentration of acetic acid ([acetic acid]_final_ = 4.0 M) induces a rapid imine condensation reaction at room temperature and thus the formation of discrete nCOF crystalline nanosheets (Fig. S2 and S3†). The increased rate of monomer consumption induces both supersaturation in crystalline nanosheets and inhibition of crystallite growth into bigger structures. Subsequently, nanosheets agglomerate by stacking on each other to form polycrystalline nanoparticles of spherical shape with rough surfaces and small protrusions (Fig. S3†); this latter phenomenon is due to the small presence of H_2_O co-solvent which favors hydrogen bonding between nanosheets. When the synthesis is performed with pure acetic acid in the absence of H_2_O ([acetic acid]_final_ = 5.0 M), small crystalline nanosheets with limited stacking are obtained without nanoparticle formation (Fig. S4†).

From a drop-cast solution (Fig. S10†), under atomic force microscopy (AFM), the TTA-DFP-nCOF appears as uniform particles with a height of 7 nm, corresponding to the stacking of ∼18 nCOF layers. In solution, they present themselves as particles with an average hydrodynamic radius of 82 nm and a polydispersity index (PDI) of 0.1 from dynamic light scattering (DLS) analysis, with no precipitation observed over time. Once isolated as solids using a freeze-drying process, the nanoparticles could be re-dispersed in H_2_O and were stable in solution for at least twelve months, during which their size distribution remained unchanged (Fig. S11†). Crystallinity of the as-synthesized material was demonstrated by powder X-ray diffraction (PXRD), which features a strong peak at 2*θ* = 4.9° assigned to the (110) plane of the regularly ordered lattice, as well as a broad peak centered at 2*θ* = 25.6°, corresponding to the reflection from the (003) plane (Fig. 1c and S12†). Seed-mediated crystallization can explain the increased crystallinity as the amorphous imine polymer formation commonly observed in nCOF powders is not observed.^44,49^ N_2_ sorption demonstrates the permanent porosity of nCOFs. The shape of the isotherm combines type I and II features, and the Brunauer–Emmett–Teller (BET) surface area is 384.5 m^2^ g^−1^ (Fig. S13†). The isotherm displays an H3-type hysteresis indicative of aggregates of plate-like particles giving rise to slit-shaped pores,^50^ similar to those of nanosheet-based materials reported elsewhere.^51,52^

FTIR analysis of the nCOF shows the characteristic imine stretching at 1617 cm^−1^, a peak at 1582 cm^−1^ attributed to the C–C=N bond of the triazine and pyridine stretching, a broad and strong peak at 1365 cm^−1^ corresponding to the C–N bond of the triazine core and a strong vibration peak at 1507 cm^−1^ corresponding to the C=C aromatic bonds, along with the disappearance of the TTA primary amine N–H stretching at ∼3320 cm^−1^ and the DFP aldehyde stretching at 1711 cm^−1^ (Fig. S14†).^53,54^ The chemical stability of the TTA-DFP-nCOF under conditions designed to simulate the stomach environment (pH 2.0) as well as the bloodstream (pH 7.4) was assessed. The TTA-DFP-nCOF remained unaffected under both conditions as confirmed through TEM imaging (Fig. S2–S5†), PXRD and the BET analysis (Fig. S17†). These features prompted us to use these nCOFs for oral insulin delivery. Collectively, these bulk characterization experiments indicate that the nanosized TTA-DFP-nCOFs form high quality imine-linked nCOFs.

Insulin-loading of the TTA-DFP-nCOF was first realized by soaking the TTA-DFP-nCOF (5 mg) in insulin solution (5 mg in 2.5 mL HEPES buffer, pH 7.4). Insulin loading was monitored using ^1^H NMR over a period of 24 hours (Fig. S18†) and showed that the insulin signals progressively decreased following the addition of the nanoparticles due to the encapsulation of insulin inside the TTA-DFP-nCOF. However, no conclusions could be made on the nature of interactions from the NMR experiment.

The use of dye (FITC)-labelled-insulin (insulin-FITC) facilitates the quantification of the amount of loaded insulin. Insulin-FITC uptake was measured using fluorescence spectroscopy of the supernatant to quantify the concentration of unloaded insulin-FITC (Fig. S19 and S20†) with the TTA-DFP-nCOF exhibiting an insulin-FITC loading capacity of 64.6 ± 1.7 wt% (Fig. S20†), comparable to that of previously reported insulin encapsulation in porous materials (Table S2†).^55,56^ In order to visually show the presence of insulin-FITC inside the NPs, confocal microscopy analysis was performed. The fluorescent nature of the insulin-FITC loaded TTA-DFP-nCOF (*λ*_ex_ = 488 nm, Fig. 1f and S21†) confirmed the presence of insulin-FITC in the nanomaterial.

Upon insulin loading, the PXRD pattern of TTA-DFP-nCOF/insulin was nearly flat, as compared to that of the pristine TTA-DFP-nCOF (Fig. S12† and Table 1). It is anticipated that upon loading with insulin, the periodicity in the TTA-DFP-nCOF layers is affected to accommodate insulin molecules, thus losing crystallinity.^57–60^

**Table 1:** Physicochemical characterization of the TTA-DFP-nCOF before and after insulin loading

	TTA-DFP-nCOF	TTA-DFP-nCOF/insulin
PXRD, 2*θ* (plane)	4.9° (110), 25.6° (003)	4.9° (110), 25.6° (003)
AFM height (nm)	7	12
TEM width (nm)	124	147
Hydrodynamic radius (nm), (PDI^a^)	82, (0.1)	94, (0.2)
ζ-Potential (mV)	∼−16	∼−17
BET (m^2^ g^−1^), (TPV^b^, m^3^ g^−1^)	385, (0.50)	12, (0.02)

a PDI: polydispersity index.

b TPV: total pore volume.

When the TTA-DFP-nCOF was loaded with a reduced quantity of insulin (30% loading capacity, ESI†), the PXRD pattern showed the presence of 2*θ* = 4.9° and 25.6° peaks, although their intensities decreased significantly compared to those of the pristine nCOF, confirming our hypothesis. The uniformity of TTA-DFP-nCOF/insulin is evident from AFM analysis and increased to 12 nm upon insulin encapsulation compared to that of the pristine nCOF (Fig. S10† and Table 1). AFM images also show an increase in the nanoparticle width upon insulin loading, corroborated by TEM (Fig. 1e, S6–S8,† and Table 1). This suggests the slipping of nanosheets to accommodate the insulin molecules, a fact that is supported by the loss of crystallinity shown by PXRD. The hydrodynamic radii of the nanoparticles without and with insulin loading show an increase in solvated particle size as well as an increase in polydispersity from narrowly monodisperse to moderately polydisperse, showing a slight distribution in insulin encapsulation (Table 1 and Fig. S22†). ζ-Potential measurements show no statistically significant difference in pristine- and insulin-loaded nCOF yet they are both markedly different from insulin on its own, strongly supporting insulin encapsulation within the nanoparticles, rather than on the surface (Table 1 and Fig. S23†). Following insulin loading, N_2_ sorption (Fig. S13†) exhibits a Type IV isotherm, typical of mesoporous materials, with a low-pressure H4-type hysteresis which either indicates swelling of a non-rigid porous structure^50^ or, as observed by Bertier *et al.* for shales,^61^ that insufficient equilibration is achieved during measurements because of slow N_2_ diffusion in ultramicropores or that significant micropores exist but whose access is blocked.

Further evidence for insulin-loading between the nanosheets *versus* inclusion or diffusion through the micropore channels formed by stacking of nCOF layers is obtained from the size of the protein molecules (2.5–3 nm)^62^ compared to the diameter of the micropore channels (1.7 nm, Fig. S13†). Insulin molecules are likely intercalated between several layers, favored by the small size of the TTA-DFP-nCOF particles. Simulation of the adsorption of insulin molecules between TTA-DFP-nCOF layers was performed following a simulated annealing process, where one insulin monomer was included between two sets of three ABC-stacked layers (Fig. 1g). A loading amount corresponding to ∼70 wt% was calculated which is comparable to the maximum loading amount achieved experimentally (calculation details in the ESI†). To accommodate the insulin molecules, each set of layers was separated by 2.5 nm along the stacking direction during the simulation process. The resulting minimum energy conformation displayed in Fig. 1g shows how the insulin molecules are accommodated and interact with the nCOF layer atoms, which is also favored by the presence of pores, towards which some of the insulin terminal peptides are pointing. TEM dispersive X-ray spectroscopy (TEM-EDX) indicates that insulin is uniformly distributed throughout the TTA-DFP-nCOF (Fig. S9†).

In order to identify the nature of interactions between the TTA-DFP-nCOF and insulin, we conducted Fourier-transform infrared spectroscopy (FTIR) and X-ray photoelectron spectroscopy (XPS) studies. The FTIR spectrum of insulin displayed characteristic protein peaks: (i) at 2800–3000 cm^−1^ corresponding to the CH_2_ stretching bond, (ii) at 1645 cm^−1^ for amide I and (iii) at 1535 cm^−1^ corresponding to amide II primarily due to the C=O stretching vibration (Fig. S15 and S16†).^63^ After insulin entrapment in the TTA-DFP-nCOF, we observed the evolution of a new set of peaks at 2800–3000 cm^−1^ and at 1657 cm^−1^ corresponding to the CH_2_ and C=O (amide I) stretching of insulin, with significant shifts (Fig. S15 and S16†). The peak at 1535 cm^−1^ overlaps with the aromatic C=C stretching of the TTA-DFP-nCOF. In addition, the disappearance of the nCOF imine bond at 1617 cm^−1^ was also observed. These observations could be associated with weak insulin–TTA-DFP-nCOF interactions between the amide I C=O group of insulin and the nCOF imine bond, as reported by Sarmento *et al.*^64^ and Boonsongrit *et al.*^65^

The presence of sulfur along with the net increase of oxygen (around 10-fold) seen in the XPS survey spectrum of the TTA-DFP-nCOF/insulin compared with the survey spectrum of the TTA-DFP-nCOF represents clear evidence of insulin's presence within the nanoparticles (Fig. S24a and S26a†), since sulfur is present in insulin due to cysteine amino acids while it is absent in the nCOF (Fig. S25a†). The C 1s high resolution XPS spectrum of TTA-DFP-nCOF/insulin (Fig. S26b†) comprises four peaks at 283.4, 285.1, 286.3 and 288.2 eV attributed to the C=C sp^2^, C–C sp^3^, C–O and C–N bonds and carbon in carboxylic or amide groups, respectively.^66^ A significant increase in the C sp^3^ percentage could be observed in the deconvoluted C 1s peak of TTA-DFP-nCOF/insulin compared with C 1s of the nCOF (Fig. S24b†). This increase could be from the contribution of C sp^3^ present in the side chains of amino acids that exist in the structure of insulin. Fig. S25c† shows the O 1s XPS survey spectrum in insulin, where the peak is composed of three components centered at 531.4, 532.3 and 533.5 eV attributed to oxygen in carboxylate, amide and alcohol groups respectively.^67^ After loading the TTA-DFP-nCOF with insulin, an important decrease (*ca.* 12%) in the percentage of oxygen assigned to carboxylate was observed (Fig. S26c†). The deconvoluted N 1s spectrum of the TTA-DFP-nCOF (Fig. S24d†) indicates the presence of three distinct peaks at (i) 399.0 eV corresponding to the imine nitrogen, (ii) 399.9 eV associated with pyridinic nitrogen, and (iii) 400.7 eV attributed to quaternary nitrogen.^53^ On the other hand, the N 1s spectrum for TTA-DFP-nCOF/insulin exhibits the contribution of nitrogen from amino and amide groups present in insulin (Fig. S26d†). Additionally, a decrease in the percentage of the quaternary amine (∼8%) in the N 1s peak of the mixture compared with that of the TTA-DFP-nCOF was observed. The decrease in the percentage of oxygen assigned to carboxylate existing in insulin and the percentage of quaternary amine existing in the TTA-DFP-nCOF strongly indicate that the interactions occur between the carboxylate groups of insulin and the quaternary amine groups of the TTA-DFP-nCOF.

**Fig. 2:**
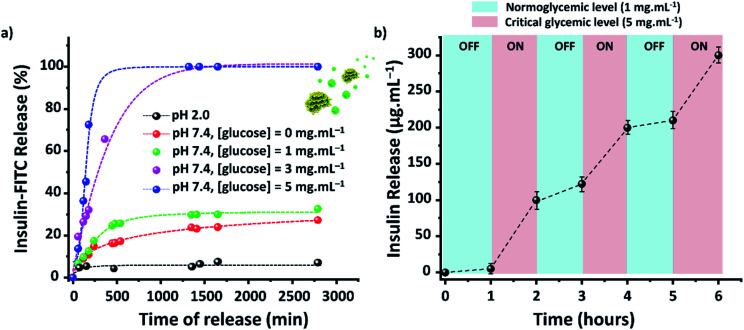
TTA-DFP-nCOF/insulin presents a glucose controlled release mode with delayed release and pH sensitivity. (a) *In vitro* accumulated insulin-release from the TTA-DFP-nCOF/insulin-FITC at 37 °C in PBS (10 mM) and pH 2.0 (black), or pH 7.4 in several glucose concentrations ([glucose] = 0 (red), 1 (green), 3 (pink) and 5 (blue) mg mL^−1^). (b) Pulsatile release profile of TTA-DFP-nCOF/insulin-FITC at 37 °C as a function of glucose concentration ([glucose] = 1 *versus* 5 mg mL^−1^). Error bars indicate ±S.D. of triplicate experiments.

The efficacy of TTA-DFP-nCOF/insulin-FITC *in vitro* was tested against both simulated gastric and intestinal fluids (SGF, pH 2.0; SIF, pH 7.4). Quantification of insulin-FITC release was carried out using fluorescence spectroscopy (ESI,† Section 4) and the experiments showed insignificant insulin release in both SGF (<5%) and SIF (<15%) following 24 h incubation (Fig. 2a).

The circular dichroism (CD) structure of the recovered insulin from TTA-DFP-nCOF/insulin under acidic conditions for 24 h displays the same pattern as that of native insulin (Fig. S29†). In order to confirm the stability of the TTA-DFP-nCOF/insulin, we performed a DLS study at pHs 7.4 and 2.0 and in the presence of lysozyme (an enzyme abundant in secretions including tears, saliva, and mucus) over a period of 24 hours, followed by TEM imaging. The size and morphology of TTA-DFP-nCOF/insulin do not vary under any of the conditions mimicking that of the stomach. Confinement of insulin between nanosheets of the nCOF nanoparticles protects it from unfolding and degradation, thus providing the safeguard necessary for oral delivery.^55^

Evaluation of the *in vitro* capacity for TTA-DFP-nCOF/insulin-FITC to respond to hyperglycemia-triggered drug release was investigated against various glucose concentrations; control, normal and diabetic, with [glucose] = 0, 1, 3 and 5 mg mL^−1^, respectively, in PBS (10 mM, pH = 7.4). Under control conditions, only 12% of insulin was released over a 24 h incubation period, rising to 28% for normoglycemia, indicating the slow, natural release of insulin. Importantly, however, under hyperglycemic conditions exhibited by diabetic patients (3 mg mL^−1^) there was almost 100% insulin release after 7.5 h of incubation (Fig. 2a).

With glucose levels naturally fluctuating between hunger and satiation,^68^ the on-off regulation of insulin release was monitored between the normal and hyperglycemic state every 1 h, with the insulin release profile of TTA-DFP-nCOF/insulin-FITC displaying a pulsatile pattern (Fig. 2b).

Compared to the size of insulin (*d* = 2.5–3.0 nm),^62^ glucose is a small molecule (*d* = 0.8 nm) that can fit inside the nCOF pores (1.7 nm). The TTA-DFP-nCOF can take up to 18 wt% of glucose (24 h, 5 mg mL^−1^) due to hydrogen bonding between the numerous hydroxyl groups and the nitrogen atoms of the framework (Fig. S32–S35†). To confirm that insulin release is specifically triggered by glucose, TTA-DFP-nCOF/insulin-FITC was incubated at 37 °C for 24 hours in the following: (i) human serum; (ii) a mix of 11 amino acids; and (iii) a saline solution of fructose (3 mg mL^−1^) or sucrose (3 mg mL^−1^; Fig. S27 and S28†). There was limited release of insulin observed up to 24 h under all four conditions tested, with a maximum insulin-FITC release of 15% in human serum, 3% in amino acids, 22% in fructose, and 13% in sucrose. However, after incubation with fructose or sucrose, and adjusting the glucose concentration to mimic hyperglycemia (3 mg mL^−1^), a burst release of insulin was observed, confirming the specific glucose-responsiveness of this nCOF system. The size and the polarity of the sugar molecules play a key role in the release of insulin. Sucrose is a larger molecule (1.06 nm) than fructose (0.8 nm) and glucose (0.8 nm) and therefore its access to the pores seems hindered. The differences in stereochemistry between fructose and glucose determine a relevant change in polarity, which might have a fundamental impact on the behavior of these molecules with the nCOF structure. Fructose being more hygroscopic than glucose has its hydroxyl groups less available to interact with the nitrogen atoms of the framework, which might explain the selective release of insulin with glucose.^69^ In addition, the small sized amino acids are likely to be zwitterionic at neutral pH, and this neutral-yet-charged species clearly prevents them from entering the nCOF structure.^70,71^

Under hyperglycemic conditions, glucose is forcefully diffused through the micropores of the nCOF displacing insulin from between the nanosheets. After 24 hour of hyperglycemic interactions, the TTA-DFP-nCOF was found to be almost insulin free. TTA-DFP-nCOF/glucose displayed a surface charge of −25.8 mV due to the large number of hydroxyl functional groups of glucose on the surface and within the framework (Fig. S33†). TTA-DFP-nCOF/insulin with glucose incubation displayed a charge of −19.6 mV. This strongly indicates the glucose concentration dependence of insulin displacement. The size of glucose results in preferential filling of nCOF pores with glucose, which are too small for insulin. However, once these pores are filled at normoglycemic concentrations, increasing the glucose concentration leads to glucose molecules penetrating the nanosheets, thereby displacing the insulin and forcing it out of the nanoparticle. However, the extent of glucose absorption into the micropores of the nCOF does not hinder the oral delivery of insulin. The TTA-DFP-nCOF can take up a maximum of 18 wt% of glucose under unrealistic hyperglycemic conditions which are considered unlikely to occur in a patient. Therefore, it is doubtful that TTA-DFP-nCOF/insulin would result in a hypoglycemic state by causing a sudden drop in glucose levels *in vivo* or in a patient. TEM, PXRD and BET analysis of the nCOF after insulin release under hyperglycemic conditions showed a decrease in size, restoration of the crystallinity to a certain degree, and increased BET surface area compared to TTA-DFP-nCOF/insulin (Fig. S35 and S36†). The preservation of insulin's secondary structure following release from the nCOF was assessed using circular dichroism and was found to be similar to that of original insulin (Fig. S30†); thus, nCOF-encapsulated insulin maintains both its structure and properties during transport and release, and, with this system exhibiting a glucose-triggered release mechanism, is an ideal candidate for the treatment of diabetes.

*In vitro* viability studies were carried out on 10 different cell lines (liver, colon, cervix, ovary, breast, kidney and brain, Fig. S37†) to demonstrate the potential of the TTA-DFP-nCOF as a biocompatible delivery vehicle. Both the TTA-DFP-nCOF and TTA-DFP-nCOF/insulin elicited no cytotoxic effects at TTA-DFP-nCOF concentrations up to 1 mg mL^−1^ following 48 h of incubation, indicating excellent biocompatibility and, therefore, great potential for oral application.

TEM was used to investigate the effects of TTA-DFP-nCOF/insulin on cellular structures and their interactions with organelles on 2 colon cell lines (RKO and HCT-116, 4 h incubation time) and the cells were analyzed 4 h, 24 h and 48 h post-treatment (50 μg mL^−1^; Fig. S38–S40†) since the material needs to cross the intestinal barrier. All samples treated with TTA-DFP-nCOF/insulin exhibited the regular ultrastructure of the RKO and HCT-116 cells, with a roundish cellular shape, a plasma membrane rich in protrusions (such as microvilli), a well-developed rough endoplasmic reticulum, Golgi apparatus, and mitochondria, which indicate the maintenance of metabolically active cells. Significant amounts of TTA-DFP-nCOF/insulin can be visualized within some of the treated cells and at their surface. Membrane deformation was also observed, confirming the internalization of TTA-DFP-nCOF/insulin by endocytosis. Over time, in both cell lines the TTA-DFP-nCOF/insulin could be found inside cell vacuoles in the perinuclear region but no more on the membrane; cells continued to grow and divide, confirming that TTA-DFP-nCOF/insulin is non-toxic and safe, with no deleterious effect on cell morphology, viability, and mitochondrial health, and does not lead to the production of any reactive oxygen species.

As the purpose of the material is to enter the bloodstream, a hemolysis assay was carried out to determine its biocompatibility and immunotoxicity to human erythrocytes.^72–74^ The hemolytic rates (HR) of the samples were found to be <2%, which, according to ASTM F 756-08 (Standard Practice for Assessment of Hemolytic Properties of Materials),^75^ is considered to be non-hemolytic as it falls below the HR threshold of <5%. Therefore, the hemolytic test results (Fig. S41†) indicate that the TTA-DFP-nCOF and TTA-DFP-nCOF/insulin are biocompatible and non-immunotoxic to human blood erythrocytes. This could be somehow related to the negatively charged surface of our nanoparticles since nanoparticles with a negative surface charge were proven to be not hemolytic.^76–79^

We next assessed the ability of the TTA-DFP-nCOF/insulin to cross the intestinal barrier in *ex vivo* experiments (Fig. S42†). The use of nanoparticles can improve the transporting ability of proteins through the intestinal wall while protecting them against degradation in gastric fluid.^80,81^ TTA-DFP-nCOF/insulin-FITC transportation across the intestinal wall was assessed by measuring the apparent permeability using an *ex vivo* technique in excised rat small intestine using the non-everted mouse small intestine sac model.^82,83^ The transportation of TTA-DFP-nCOF/insulin from the mucosal side to the serosal side of the non-everted mouse small intestine sac was measured and quantified by fluorescence measurements. The permeability of TTA-DFP-nCOF/insulin after 3 h was calculated to be 14.76 μg cm^−2^ (corresponding to 60.8% ± 14.2 of the initial dose), while that of pure insulin was reported to be 8.02 μg cm^−2^.^84^ This indicates that incorporation of insulin into the TTA-DFP-nCOF resulted in an approximately two fold increase of the permeability of insulin. Permeation data correlate with accumulation in the gut wall. This can possibly be attributed to enhancement of the surface area leading to a higher rate of insulin-FITC diffusion.^84^ The serosal side were collected and TEM imaging was performed, confirming that TTA-DFP-nCOF/insulin-FITC crossed the intestinal barrier. As shown in Fig. S43,† TTA-DFP-nCOF/insulin-FITC was present without modification of morphology or size on the serosal side. This accumulation may result from the potential cellular internalization of nanoparticles. Therefore, upon concluding the experiments, tissues were washed with normal saline, and nanoparticle accumulation in the gut wall was investigated by TEM (Fig. S44†). TEM images of the intestinal sections show their morphology with intact microvilli and the underlying architecture of the ileal mucosa. TTA-DFP-nCOF/insulin was located inside the goblet cells (GCs) of the intestinal tissue and excreted into the gut lumen through the secretion of intestinal GCs.^85^ These results confirm that TTA-DFP-nCOF/insulin can cross the intestinal barrier carrying the insulin cargo and does not cause obvious pathological changes in intestinal tissues.

**Fig. 3:**
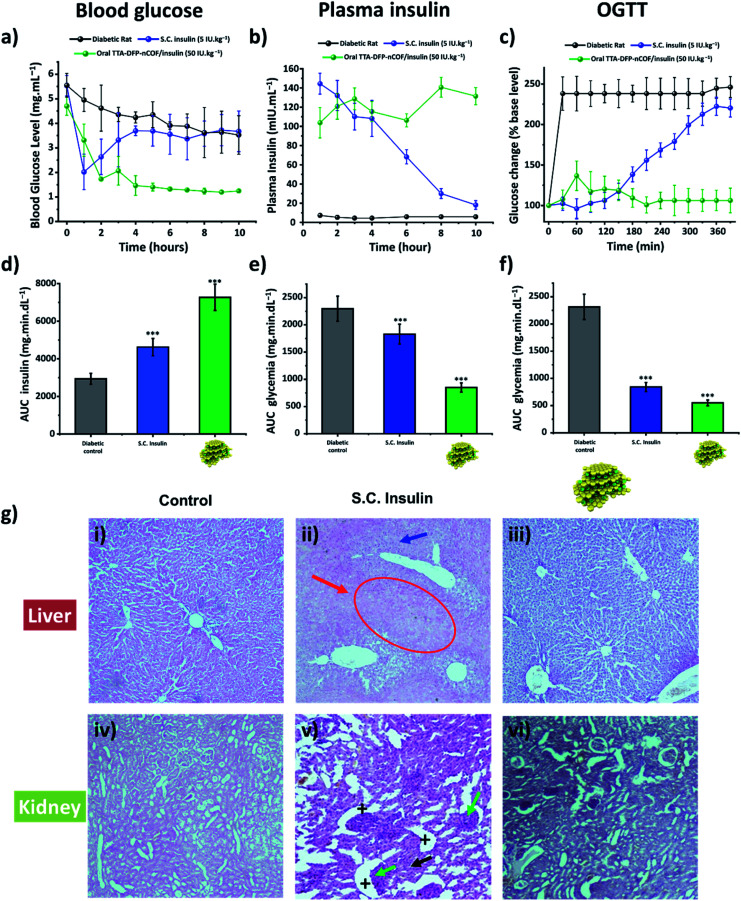
TTA-DFP-nCOF/insulin regulates glucose uptake *in vivo* without causing toxicity. (a) Blood glucose level evolution, (b) serum insulin level changes and (c) oral glucose tolerance test (OGTT) results of the STZ-induced diabetic rats over time after oral administration of TTA-DFP-nCOF/insulin (green) at an insulin dosage of 50 IU kg^−1^. The group with subcutaneous injection (S.C., blue) of insulin at 5 IU kg^−1^ was set as the positive control. Glycemic level, plasma insulin level and OGTT results of the diabetic rats (control) are illustrated in black. The corresponding area under the curve (AUC) is depicted in (d)–(f) for glycemic levels, plasma insulin levels and OGTT results, respectively. TTA-DFP-nCOF/insulin showed statistically significant differences in glycemic levels, plasma insulin levels and OGTT results compared with S.C. insulin solution and diabetic control (* *p* <0.05; ** *p* <0.01; *** *p* <0.001). Each value represents mean ± S.D. (*n* = 3). (g) Histopathological study. Sections of liver (i–iii) and kidney (iv–vi) of diabetic control rats (i and iv), S.C. insulin-injected rats (5 IU kg^−1^, (ii) and (v) and diabetic rats treated orally with TTA-DFP-nCOF/insulin (50 IU kg^−1^, (iii) and (vi) In figure (ii), the white arrow points to big hepatocytes, and the red arrow highlights necrosis of hepatocytes and a narrowing of the sinusoids. In figure (v), the + signs indicate Bowman capsules while the green arrow points to glomeruli hypertrophy, and tubule necrosis is indicated by the black arrow.

The *in vivo* pharmacological effect of glucose-responsive TTA-DFP-nCOF/insulin was evaluated by oral administration to a streptozotocin (STZ)-induced Type 1 diabetic (T1D) rat model (Fig. 3a–d and S45†).^86–89^ The changes in blood glucose levels of the diabetic rats as a function of time following oral administration of various formulations or subcutaneous injection of free-form insulin solution were determined (Fig. 3a–d and S45†). Oral delivery of the insulin-free TTA-DFP-nCOF (2 mg kg^−1^) or free-form insulin solution (50 IU kg^−1^) exhibited nearly no oral pharmacological availability as confirmed by the minimal hypoglycemic effect observed. By contrast, subcutaneous injection of free-form insulin solution (5 IU kg^−1^) resulted in a marked reduction of the blood glucose level within 1 h, though the effect was not sustained, with blood glucose levels rapidly returning to those similar to insulin-free nCOF-injected rats. However, following oral administration of TTA-DFP-nCOF/insulin (50 IU kg^−1^), a significant reduction in blood glucose levels similar to that in non-diabetic rats was observed within 2 h coupled with a sustained hypoglycemic effect for 10 h, replicating the normal glucose level of the non-diabetic control group (Fig. 3a). These results are in accordance with the plasma insulin levels which showed that TTA-DFP-nCOF/insulin-treated rats presented the highest level of plasmatic insulin. Subcutaneous insulin-injected rats experienced a peak in the plasma insulin level in the first hour after insulin injection, after which it rapidly decreased as described in the literature.^90^ The homeostatic model assessment (HOMA) is a method used to yield an estimate of insulin sensitivity, insulin resistance and β-cell function from fasting plasma insulin and glucose concentrations.^91^ Rats treated with TTA-DFP-nCOF/insulin presented a lower HOMA-IR (insulin-resistance index) and a higher HOMA-IS (insulin-sensitivity index) than the subcutaneous insulin injected rats, suggesting that the TTA-DFP-nCOF/insulin particles are better assimilated by the body than subcutaneous insulin (Table 2). Oral insulin is directly absorbed by the intestinal epithelium and reaches the liver through the portal vein, allowing maintenance of glucose homeostasis, whereas parenterally administered insulin never mimics naturally secreted insulin as it is first delivered to peripheral tissues.^92^ The calculations show that TTA-DFP-nCOF/insulin particles exhibit a high bioavailability (24.1%) compared to insulin-loaded particles described in the literature.^93–95^ This is in accordance with the insulin and glucose plasmatic levels observed.

**Table 2:** Measurements by the homeostatic model assessment (HOMA) of insulin resistance (HOMA-IR) and insulin-sensitivity (HOMA-IS) of β-cell function using the changes in insulin and glucose concentrations after subcutaneous insulin and TTA-DFP-nCOF/insulin treatment of diabetic rats. Each value represents mean ± S.D. (*n* = 3)

	S.C. insulin	TTA-DFP-nCOF/insulin
HOMA-IR	0.6 (0.3)	0.5 (0.1)
HOMA-IS	50.4 (11.0)	54.4 (13.2)

The oral glucose tolerance test (OGTT) was used to assess the ability of the body to take up glucose in post-prandial conditions and to evaluate the sensitivity of the body to endogenous insulin.^96^ Test animals first received the TTA-DFP-nCOF/insulin by gavage or subcutaneous insulin injection. Three hours after that, coinciding with the serum insulin peak previously observed, the animals received 2.5 g kg^−1^ of glucose dissolved in 1 mL of water, and then the glycaemic level was evaluated over a period of 280 min (Fig. 3c–f) and compared to subcutaneous insulin-injected group and the diabetic control group. Subcutaneous insulin severely decreases the plasma glucose level within the first two hours after glucose gavage, as described in the literature.^97^ Herein, from 150 min onwards, the glycaemic level begins to increase, almost reaching control values. It is reported that recurrent hypoglycaemic episodes caused by subcutaneous insulin therapy compromise the function and integrity of brain cells.^98^ The hypoglycaemic activity of TTA-DFP-nCOF/insulin, while initially more moderate than that of subcutaneous insulin, from 120 min onward, results in low and stable glycaemia for the duration of the study. Oral administration of insulin allows high concentrations of insulin to enter the portal vein without sustained peripheral hyperinsulinemia, thereby preventing neuropathy and retinopathy.^68^ Thus, oral delivery of TTA-DFP-nCOF/insulin results in low and stable glycaemia from 90 min.

Diabetes mellitus is often associated with alterations of kidney and liver functions in rats; therefore, we studied the impact of TTA-DFP-nCOF/insulin on these vital functions.^99^Fig. 3g shows the histopathological study of the liver and kidney to detect organ pathology in non-diabetic rats and S.C. insulin- and TTA-DFP-nCOF/insulin-treated rats. The S.C.-insulin treated group displays the commonly observed damage to the liver and kidney due to STZ administration to induce diabetes.^100–102^ Regarding the TTA-DFP-nCOF/insulin treated rats, it can be seen that there is no damage caused to any of these organs, suggesting that TTA-DFP-nCOF/insulin is non-toxic but also that oral delivery of insulin can in fact inhibit histopathological alterations induced by diabetes in rats.^100,101^

Furthermore, an exploration of biochemical markers for liver and kidney functions in the blood can provide additional useful information to identify the beginnings of toxic effects.^103^ Urea and creatinine are biomarkers commonly used to identify alterations in kidney function, while aspartate aminotransferase (ASAT) and alanine aminotransferase (ALAT) are biomarkers of liver damage; the literature reports elevations of these 4 parameters in diabetic rats (Fig. S46†).^104^ Overall, rats treated with TTA-DFP-nCOF/insulin present similar levels of urea, creatinine, ASAT and ALAT to the non-diabetic group (Fig. S46†), demonstrating that TTA-DFP-nCOF/insulin particles are not only non-toxic, but can also enhance kidney and liver functions compared to the diabetic control. Oral insulin delivery is described as being beneficial to kidney and liver functions.^68,105^

## Conclusions

In conclusion, we have successfully prepared and tested, *in vitro* and *in vivo*, a nanoscale imine-covalent organic framework (TTA-DFP-nCOF) as an oral insulin delivery system. The TTA-DFP-nCOF's crystalline and porous nature allows the highest loading of insulin to be achieved, with evidence showing that insulin is located between layers of the nCOF nanosheets, rather than in the porous channels. The TTA-DFP-nCOF was proven to protect encapsulated insulin *in vitro* under harsh conditions mimicking the stomach environment, while the sustainable release of insulin was accomplished under hyperglycemic conditions; importantly, insulin maintained its activity upon release from TTA-DFP-nCOF/insulin. The oral administration of TTA-DFP-nCOF/insulin to STZ-induced diabetic rats led to a continuous decline in the fasting blood glucose level within 2 to 4 h, and the hypoglycemic effect remained unchanged over 10 h *in vivo* showing high insulin bioavailability without systemic toxicity. The potential for this TTA-DFP-nCOF based oral insulin delivery system to replace traditional subcutaneous injections and enhance the uptake of insulin therapy amongst those in need has been demonstrated. We are currently in the process of testing other imine-linked COFs in order to establish a structure–activity relationship and decode the different parameters that make a given COF suitable for oral delivery of insulin.

## Author contributions

F. B. and A. T. conceived the idea and led the project; F. B. synthesized and characterized the insulin loaded nCOFs; S. S. and R. J. performed the AFM analysis; T. P. synthesized the DFP linker and performed the PXRD and NMR analysis; G. D. synthesized TTA linker; F. B. and R. P. performed the TEM analysis; H. T. and M. A. performed the XPS analysis and data analysis to understand the interactions between the nanoparticles and the insulin; F. B. performed the *in vitro* biological experiments; M. K. and F. B. performed the gastro-intestinal study; F. B., S. A. T. and R. P. prepared and analyzed the biological samples for TEM study of nanoparticles cell internalization; F. G. performed the simulation of the nCOFs structure as well the insulin and nCOF interactions; N. K., F. B. S. and N. M. S. performed *in vivo* TD1 rat study and participated in data analysis; F. B., J. W. and A. T. prepared the manuscript, and all authors contributed to the discussion of results and the final version.

## Conflicts of interest

There are no conflicts to declare.

## Supplementary Material

SC-012-D0SC05328G-s001
