# Supplementary material for: In vivo oral insulin delivery via covalent organic frameworks
Source: Chem Sci. 2021 Apr 6;12(17):6037–47. doi: 10.1039/d0sc05328g (PMC8098678; doi:10.1039/d0sc05328g)
Supplement: SC-012-D0SC05328G-s001 [file SC-012-D0SC05328G-s001.pdf]

Electronic Supplementary Material (ESI) for Chemical Science.
This journal is © The Royal Society of Chemistry 2021

Supporting Information

In Vivo Oral Insulin Delivery via Covalent Organic Frameworks

*Farah Benyettou, Nawel Kaddour, Thirumurugan Prakasam, Gobinda Das, Sudhir Kumar Sharma, Sneha Ann Thomas, Fadia Bekhti-Sari, Jamie Whelan, Mohammed A. Alkhalifah, Mostafa Khair, Hassan Traboulsi, Renu Pasricha, Ramesh Jagannathan, Nassima Mokhtari-Soulimane, Felipe Gandara, Ali Trabolsi**

Table of Contents

1. Reagents and techniques 3
2. Synthesis 5
2.1. Synthesis of 2,6-diformylpyridine (DFP) and 4,4',4''-(1,3,5-triazine-2,4,6-triyl)trianiline (TTA) 5
2.2. TTA-DFP-nCOF synthesis 6
2.3. Control experiment: TTA-DFP-nCOF synthesis in pure acetic acid 6
2.4. Insulin loading in TTA-DFP-nCOF 7
2.5. Insulin-FITC loading in TTA-DFP-nCOF 7
2.6. Insulin-loading in TTA-DFP-nCOF to achieve 30 % loading capacity 7
2.7. Glucose loading in TTA-DFP-nCOF 7
3. Characterizations 8
3.1. High resolution transmission electron microscopy (HRTEM) 8
3.2. AFM Analysis 15
3.3. Dynamic light scattering (DLS) characterization 15
3.4. Powder X-ray diffraction (PXRD) measurements 16
3.5. N_2 adsorption-desorption experiments 17
3.6. Fourier Transform infrared (FTIR) spectroscopy 18
3.7. TTA-DFP-nCOF characterization in stomach conditions (pH = 2) 22
3.8. ^{1}H NMR spectroscopy 23
3.9. Determination of the amount of insulin-FITC loaded in TTA-DFP-nCOF using fluorescence spectroscopy 24
3.10. DLS and Zeta(ζ)-potential characterization of TTA-DFP-nCOF/insulin 26
3.11. X-ray photoelectron (XPS) spectroscopy 27
4. pH- and glucose dependent Insulin-FITC Release from TTA-DFP-nCOF by fluorescence emission spectroscopy 31
4.1. Insulin release conditions 31
4.2. Circular dichroism (CD) spectroscopy 34
4.3 Stability study of TTA-DFP-nCOF/insulin 37
4.4. Glucose interaction 38
5. *In vitro* biological studies 42
5.1. Cell culture 42
5.2. *In vitro* cell viability 42
5.3. Intracellular distribution of nanoparticles using TEM analysis 45
5.4. Hemolysis assay 49
6. *Ex Vivo* permeation study across mouse intestinal sac 52
7. *In vivo* animal assessments 55
7.1. Animal 56
7.2. T1D Induction 56
7.3. *In vivo* hypoglycemic effect 56
7.4. *In vivo* plasma insulin level 58
7.5. *In vivo* oral glucose tolerance test (OGTT). 58
7.6. Histopathology 59
7.7. Biochemical determinations 59
References 60

1. Reagents and techniques

All reagents and starting materials were purchased from Sigma-Aldrich and used without further purification. The precursor, 2,6-diformylpyridine (DFP) was synthesized according to the published procedure with no modifications. Deionized water was used from Millipore Gradient Milli-Q water purification system. Thin-layer chromatography (TLC) was performed on silica gel 60 F254 (E. Merck). The plates were inspected under the UV light. Column chromatography was performed on silica gel 60F (Merck 9385, 0.040–0.063 mm). Infrared spectra were recorded on an Agilent Technologies Cary 600 Series FTIR Spectrometer using the ATR mode. PXRD patterns of the samples were recorded by using an X-ray Panalytical Empyrean diffractometer. High resolution transmission electron microscopy (HRTEM) images were obtained using a Talos F200X Scanning/Transmission Electron Microscope (STEM) with a lattice-fringe resolution of 0.14 nm at an accelerating voltage of 200 kV equipped with CETA 16M camera. The high resolution images of periodic structures were analyzed using TIA software. N_2 adsorption-desorption isotherms were obtained at 77 K using Micrometrics ASAP 2020 surface area analyzer. The topography of the self-templated samples was analyzed by dynamic atomic force microscopy (5500 Atomic Force Microscope; Keysight Technologies Inc., Santa Rosa, CA). We acquired topography, phase and amplitude scans simultaneously. Silicon cantilevers (NanosensorsTM, Neuchatel, Switzerland) with resonant frequencies of 250–300 kHz and force constants of 100– 130 Nm^{-1} were used. The set point value was kept at 2.5V. AFM scans were collected at 1024 points/lines with scan speed of 0.20 at fixed scan angle of 0°. Scan artifacts were minimized by acquiring a typical scan at an angle of 90o under identical image acquisition parameters. We used GwyddionTM free software (version 2.47), an SPM data visualization and analysis tool for post-processing the AFM scans. Emission spectra in water at room temperature were recorded on a Perkin Elmer LS55 Fluorescence Spectrometer. Dynamic light scattering (DLS) measurements were performed on a Malvern Zetasizer NanoSeries to obtain the size and ζ-potential of the nanoparticles. The XPS experiments were carried out on a Kratos Axis Ultra DLD spectrometer under a base pressure of $\sim 2\times10^{-10}$ mbar. A monochromated Al Kα X-ray source (1486.69 eV) was used to irradiate samples at room temperature. Far-UV spectra were recorded between 200 and 280 nm on a Chirascan CD spectrometer (Applied Photophysics, UK) with the lamp supplied with a flow of nitrogen. Fifty microlitres of the solution were added to a 0.1 mm path-length quartz cuvette (Hellma, UK) and the measurements were carried out at 20 °C (1 nm bandwidth resolution and 1 s acquisition time). Typically, at least two scans were recorded, and baseline and HEPES spectra were subtracted from each spectrum. Data were processed using Applied Photophysics Chirascan Viewer and Microsoft Excel. Phase contrast and fluorescence images were observed on an Olympus FV1000MPE confocal scanning microscope. Flow cytometry analyses were performed on Accuri C6 Flow Cytometer. The most favorable location of insulin molecules between COF layers was calculated with a simulated annealing process, using the Adsorption Locator module of Biovia Materials Studio. For this, the TTA-DFP COF structure was first modified by separating sets of three layers at a 25 Å distance, to allow the incorporation of insulin molecules. The insulin monomer was obtained from the 1zni structure of the protein data bank.[1] One insulin monomer was incorporated *per* nCOF unit cell, which corresponds to ~ 70

wt%. The Monte Carlo simulation was then run with the use of a universal forcefield after charge assignment, and the conformation with lowest adsorption energy was selected.

Table 1. Examples of COF materials loaded with enzymes in the literature and their applications.

COF	Enzyme	Results	Ref
**TPMM COFs**	Amylase	Catalytic application in starch hydrolysis	Samui et Al., 2020[2]
**COF-42-B**	Catalase, glucose oxidase (GOx)	Enzymatic immobilization in porous matrixes	Li et Al., 2020[3]
**COF-1 and COF-5**	Insulin conjugated to glucose oxidase (GOx)	Glucose and pH dual-responsive insulin delivery carriers, *in vitro* and *in vivo* on diabetic mice	Zhang et Al., 2020[4]
**TPB-DMTP-COF**	Lysozyme	Understanding the enzyme behavior upon association within a confined space	Sun et Al., 2019[5]
**COF-ETTA-EDDA**	Lipase	Enzyme immobilization	Sun et Al., 2019[5]
**PPF-2**	Lipase	Enzyme immobilization	Oliveira et Al., 2019[6]
**TPB-DMTP-COF**	Lipase	Host material for the immobilization and stabilization of enzymes	Sun et Al., 2018[7]
**COF-DhaTab**	Trypsin	Enzyme immobilization	Kandambeth, 2014[8]

Table S2. Examples of insulin delivery systems and their loading capacities.

	Material	Loading capacity	Reference
**Insulin-loaded chitosan nanoparticles**	Chitosan/poly ɣ-glutamic acid	71.8 %	Tennagels et al.[9]
**Insulin-loaded alginate nanoparticles**	Alginate-chitosan microspheres	56.7 %	Zhang et al.[10]
	Alginate microspheres	75 %	Ribeiro et al.[11]
**Insulin-loaded dextran nanoparticles**	Alginate/dextran sulfate (ADS)-NPs	72.4 %	Lopes et al[12]

**Insulin-loaded PLGA nanoparticles**	N-trimethyl chitosan chloride-coated PLGA nanoparticles	47.0%	Sheng et al.[13]
**Insulin-loaded PLA nanoparticles**	PLA-PEG microspheres	42.7 %	Sheshala et al.[14]
**Insulin-loaded PAA nanoparticles**	Polyallylamine (PAA) grafted with cholesteryl groups	86.5 %	Thompson et al.[15]
**Insulin-loaded nanoparticles containing CPP**	Eudragit S100® (ES)-coated chitosan nanoparticles + trans-activating transcriptional peptide (Tat)	80 %	Chen et al.[16]
**Lipid-Based Nanocarriers**	Solid lipid nanoparticles	43 %	Sarmento et al.[17]
	Nanostructured Lipid Carriers (NLCs)	55.3 %	Muntoni et al.[18]
	Nanoemulsions water in oil in water (w/o/w)	47.3 %	Li et al.[19]
**Liposomes**	Biotin-modified liposomes (BLPs)	45 %	Zhang et al.[20]
**Niosomes**	Span 60 niosomes	28.8 %	Ning et al.[21]
**Micelles**	Zwitterionic betaine polymer micelle	6.2 %	Han et al.[22]
**Microgel**	Glucose-responsive microgel	44.6 %	Gu et al.[23]
**Inorganic nanoparticles**	Chondroitin sulfate (CS)-capped gold nanoparticles (AuNPs)	90.1 %	Cho et al.[24]
	Silica nanoparticles	7.4 %	Zhao et al.[25]
	Zirconium Phosphate	28 %	Diaz et al.[26]
**Metal-organic framework**	MIL-100	35 %	Zhou et al.[27]

	ZIF-8	80.6 %	Duan et al.[28]
	NU-1000	40 %	Chen et al.[29]
	PCN-222/MOF-545	63 %	Wang et al.[30]

2. Synthesis

2.1. Synthesis of 2,6-diformylpyridine (DFP) and 4,4',4''-(1,3,5-triazine-2,4,6-triyl)trianiline (TTA)

2,6-diformylpyridine (DFP) was synthesized according to the published procedure with no modifications.[31]

4,4',4''-(1,3,5-triazine-2,4,6-triyl)trianiline (TTA): The triamine (TTA) was synthesized according to the published procedure with no modifications.[32]

2.2. TTA-DFP-nCOF synthesis

TTA-DFP-nCOF was synthesized by co-condensation of 2,6-diformylpyridine (DFP, 21 mg, 0.15 mmol, 5 equivalents) and 4,4',4''-(1,3,5-triazine-2,4,6-triyl)trianiline (TTA, 12 mg, 0.03 mmol, 1 equivalent), in 3 mL of anhydrous 1,4-dioxane in presence of 0.5 mL of acetic acid (13 M, [acetic acid]$_{final}$ = 4.0 M) at room temperature for 10 min (Figure S1). The solution was cleaned using dialysis in H_2O to obtain a stable colloidal suspension.

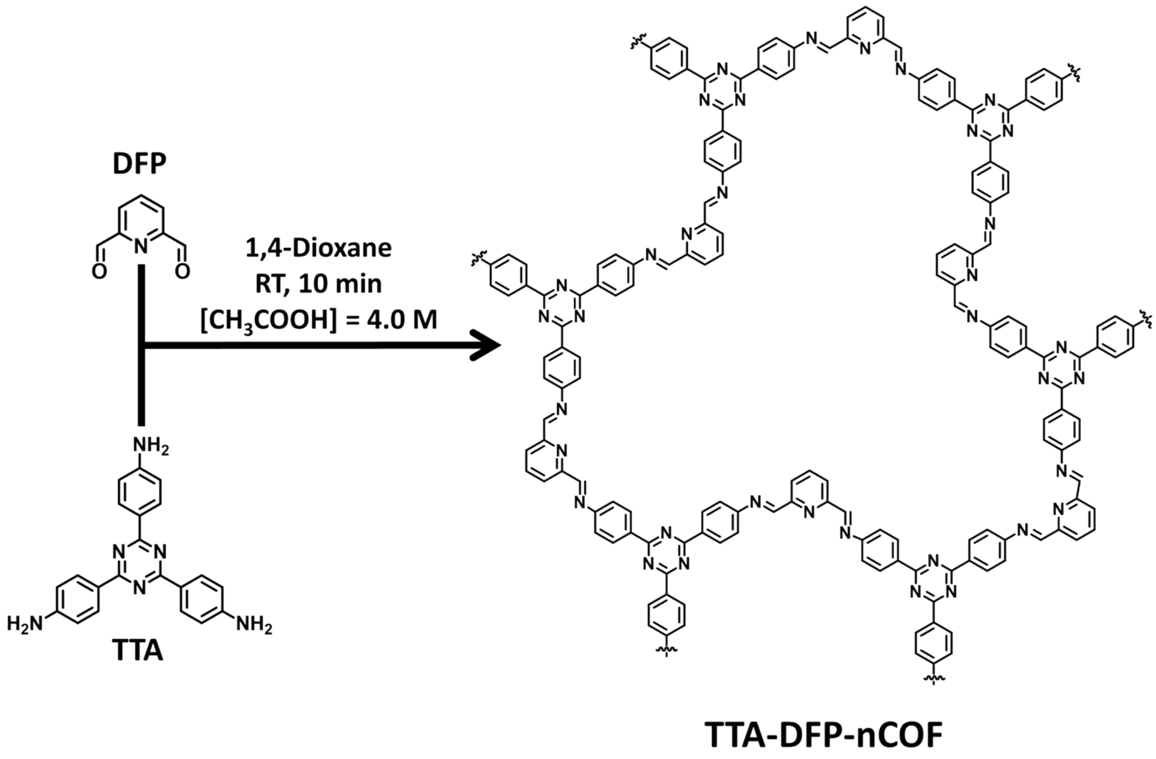

Figure S1. Synthetic route and chemical structure of TTA-DFP-nCOF.

2.3. Control experiment: TTA-DFP-nCOF synthesis in pure acetic acid

TTA-DFP-nCOF was synthesized by co-condensation of 2,6-diformylpyridine (DFP, 21 mg, 0.15 mmol, 5 equivalents) and 4,4',4''-(1,3,5-triazine-2,4,6-triyl)trianiline (TTA, 12 mg, 0.03 mmol, 1 equivalent), in 3 mL of anhydrous 1,4-dioxane in presence of 0.5 mL of acetic acid (17 M, ([acetic acid]$_{final}$ = 5.0 M) at room temperature for 10 min (Figure S1). The solution was cleaned using dialysis in H_2O to obtain a stable colloidal suspension.

2.4. Insulin loading in TTA-DFP-nCOF

Insulin was loaded into TTA-DFP-nCOF by a simple impregnation method. TTA-DFP-nCOF (5 mg) was suspended in 2 mL HEPES buffer, then a HEPES-buffered aqueous insulin solution ([insulin] = 10 mg.mL^{-1}, 1 mL) was added (TTA-DFP-nCOF:Insulin ratio = 1:2). The solution (pH 7.4) was stirred overnight at room temperature, cleaned with water several times by centrifugation and finally washed with deionized H_2O to remove unloaded insulin molecules.

2.5. Insulin-FITC loading in TTA-DFP-nCOF

Insulin-FITC (Sigma-Aldrich) was loaded into TTA-DFP-nCOF by a simple impregnation method. TTA-DFP-nCOF (5 mg) was suspended in 2 mL HEPES buffer, then a HEPES-buffered aqueous insulin solution ([insulin-FITC] = 10 mg.mL^{-1}, 1 mL) was added (TTA-DFP-nCOF:Insulin-FITC ratio = 1:2). The solution (pH 7.4) was stirred overnight at room temperature, cleaned with water several times by centrifugation and then washed with deionized H_2O to remove unloaded insulin-FITC molecules.

2.6. Insulin-loading in TTA-DFP-nCOF to achieve 30 % loading capacity

Insulin was loaded into TTA-DFP-nCOF by a simple impregnation method. TTA-DFP-nCOF (5 mg) were suspended in 2 mL HEPES buffer, then a HEPE buffered aqueous insulin solution ([insulin] = 2 mg.mL^{-1}, 0.2 mL) was added (TTA-DFP-nCOF:Insulin ratio = 2:1). The solution (pH 7.4) was stirred overnight at room temperature, cleaned with water several times by centrifugation and finally washed with deionized H_2O to remove unloaded insulin molecules.

2.7. Glucose loading in TTA-DFP-nCOF

Glucose was loaded into TTA-DFP-nCOF by impregnation. TTA-DFP-nCOF (5 mg) were suspended in 2 mL HEPES buffer, then an aqueous glucose solution ([glucose] = 5 mg.mL^{-1}, 1 mL) was added. The solution (pH 7.4) was stirred at room temperature overnight. The solution was then cleaned with water several times by centrifugation and washed with deionized H_2O to remove unloaded glucose molecules.

3. Characterizations

3.1. High resolution transmission electron microscopy (HRTEM).

High resolution transmission electron microscopy (HRTEM) images were obtained using a Talos F200X Scanning/Transmission Electron Microscope with a lattice-fringe resolution of 0.14 nm at an accelerating voltage of 200 kV equipped with a CETA 16M camera. The samples were prepared on holey carbon film mounted on a copper grid. A drop of diluted particle solution was spotted

on the grid and dried overnight at room temperature (298 K). The obtained images of periodic structures were analyzed using TIA software. All the relevant areas were marked using bright field imaging mode at spot size 3 and the marked areas were also scanned using the STEM-HDAAF mode at spot size 9 for imaging and spot size 6 for conducting the STEM-EDAX. The STEM mode helps in providing the elemental composition as it works on the principle of mass determination. Such measurements can be performed at low electron dose by collecting the high-angle dark-field signal using an annular detector. This mode is generally used to image the elements that have different masses, with the heavier mass element appearing brighter. The samples were scanned at spot size 9 and with screen current of 60 pA. The data was analyzed using Velox analytical software.

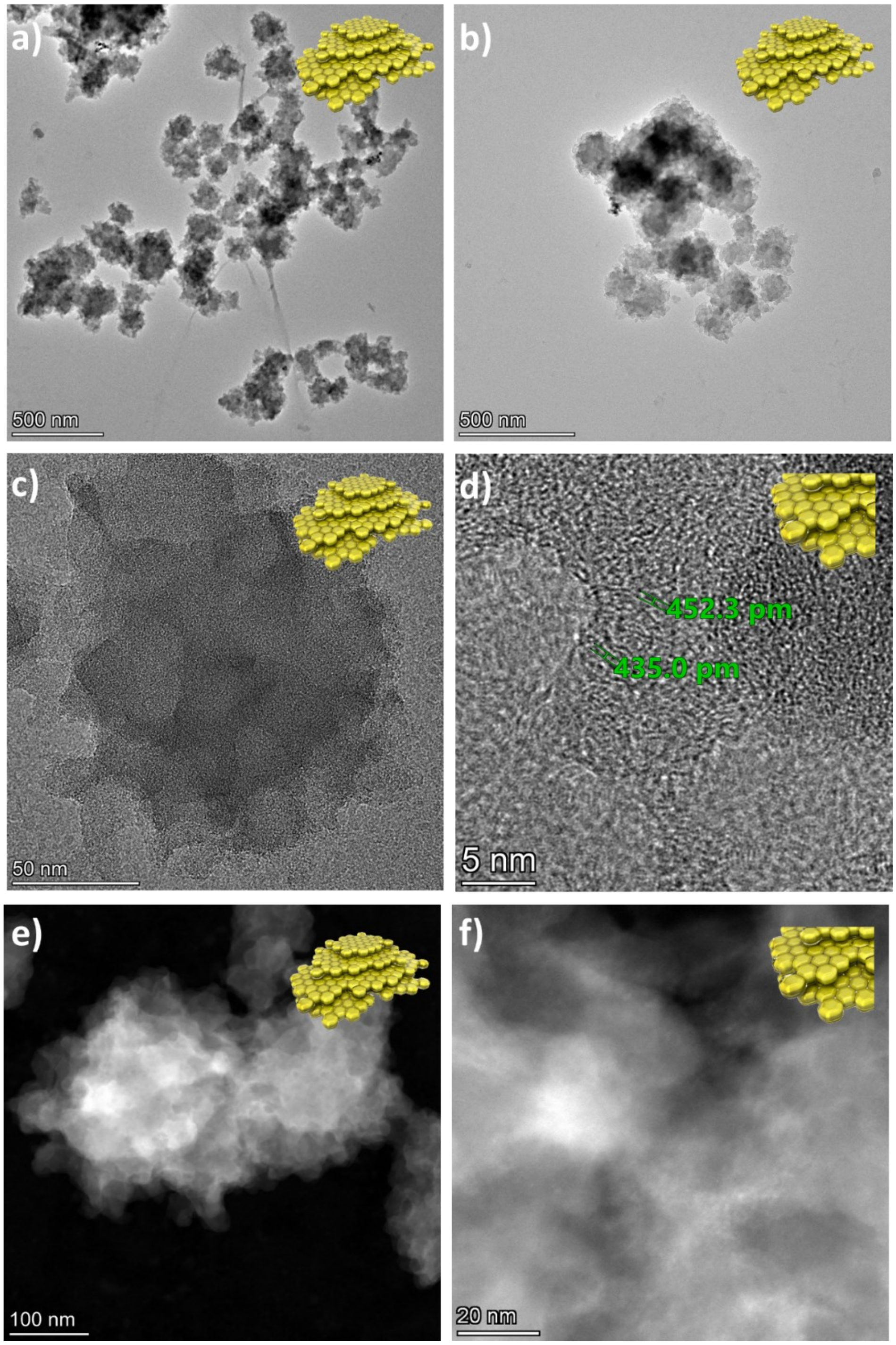

Figure S2. HR-TEM (a, b, c, d) and STEM (e, f) images of TTA-DFP-nCOF. Lattice fringe distances (d = 0.4 nm) corresponding to the (110) plane of the nCOF and confirming the crystallinity of the material are also shown.

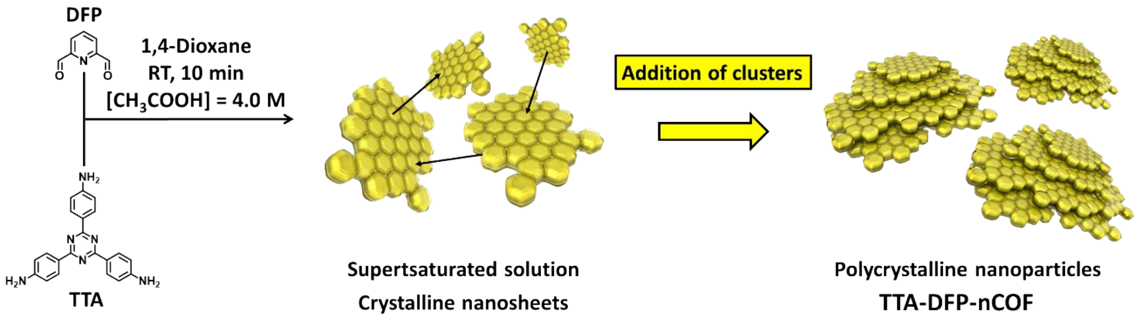

Figure S3. Proposed mechanism of TTA-DFP-nCOF formation. Black arrows represent the stacking of the nanosheets due to the small presence of H_2O co-solvent which favors hydrogen bonding between nanosheets.

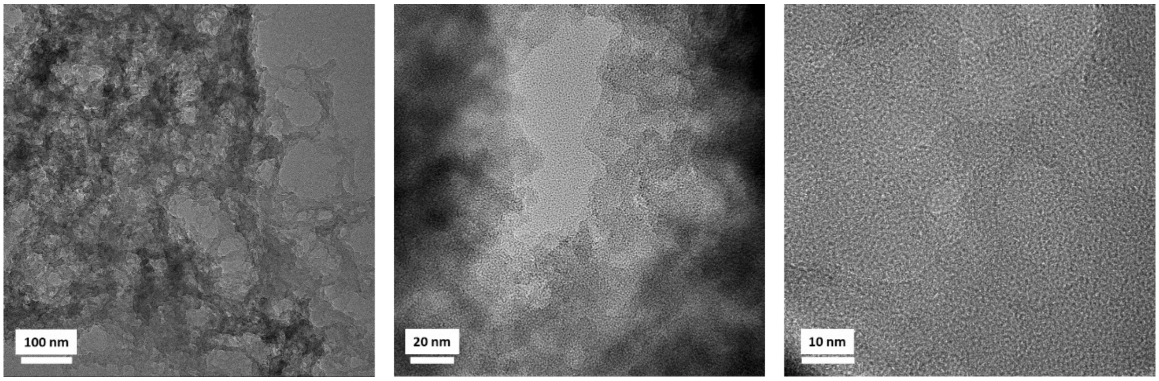

Figure S4. HRTEM images of TTA-DFP-nCOF synthesized using 0.5 mL of acetic acid (17 M, [acetic acid]$_{final}$ = 5.0 M, no H_2O co-solvent).

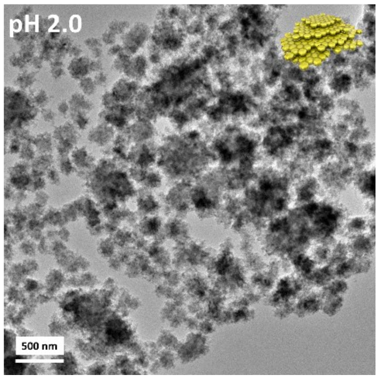

Figure S5. TEM image of TTA-DFP-nCOF suspended 24 hours at pH = 2.0 showing no alteration of the nCOF structure.

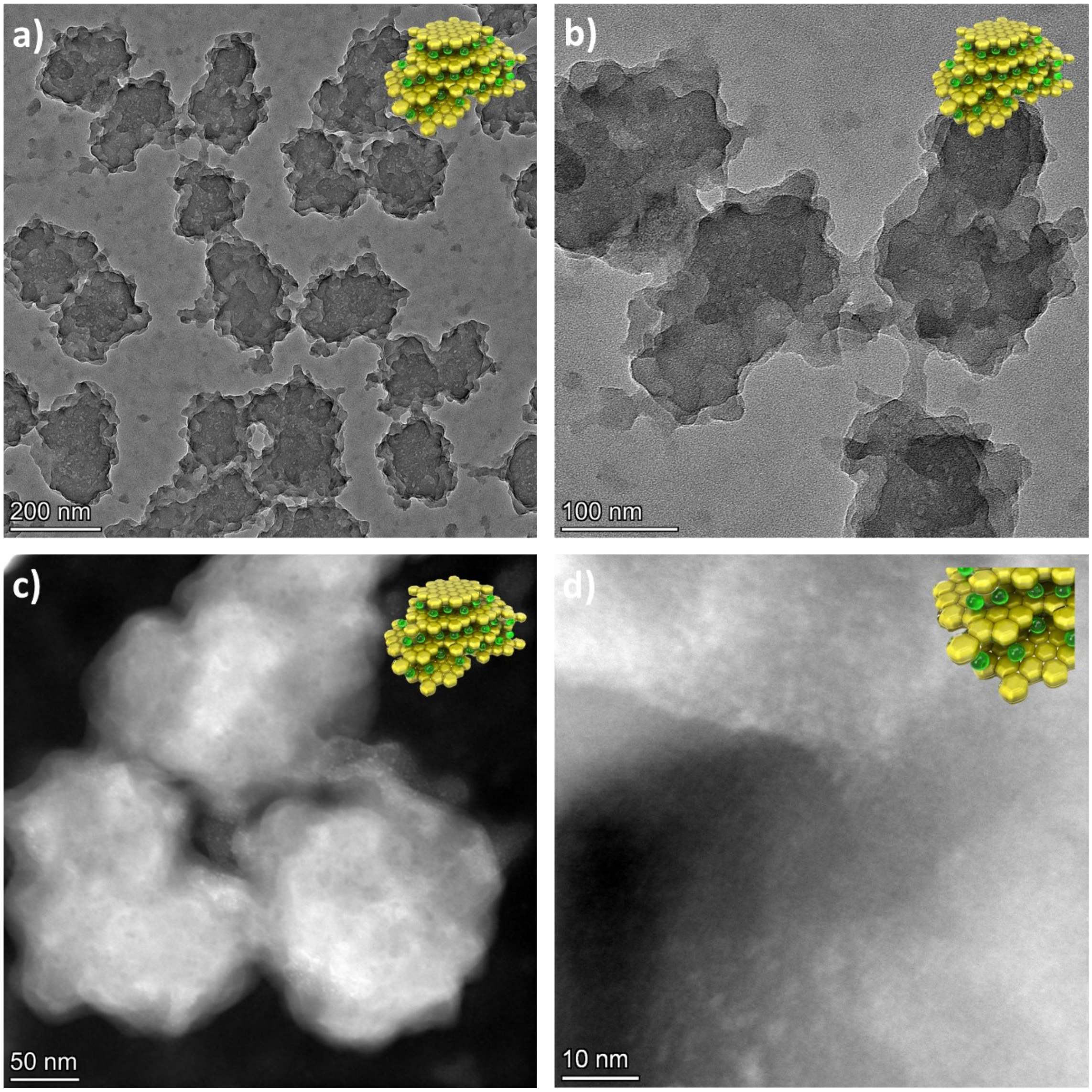

Figure S6. HR-TEM (a, b) and STEM (c, d) images of TTA-DFP-nCOF/insulin.

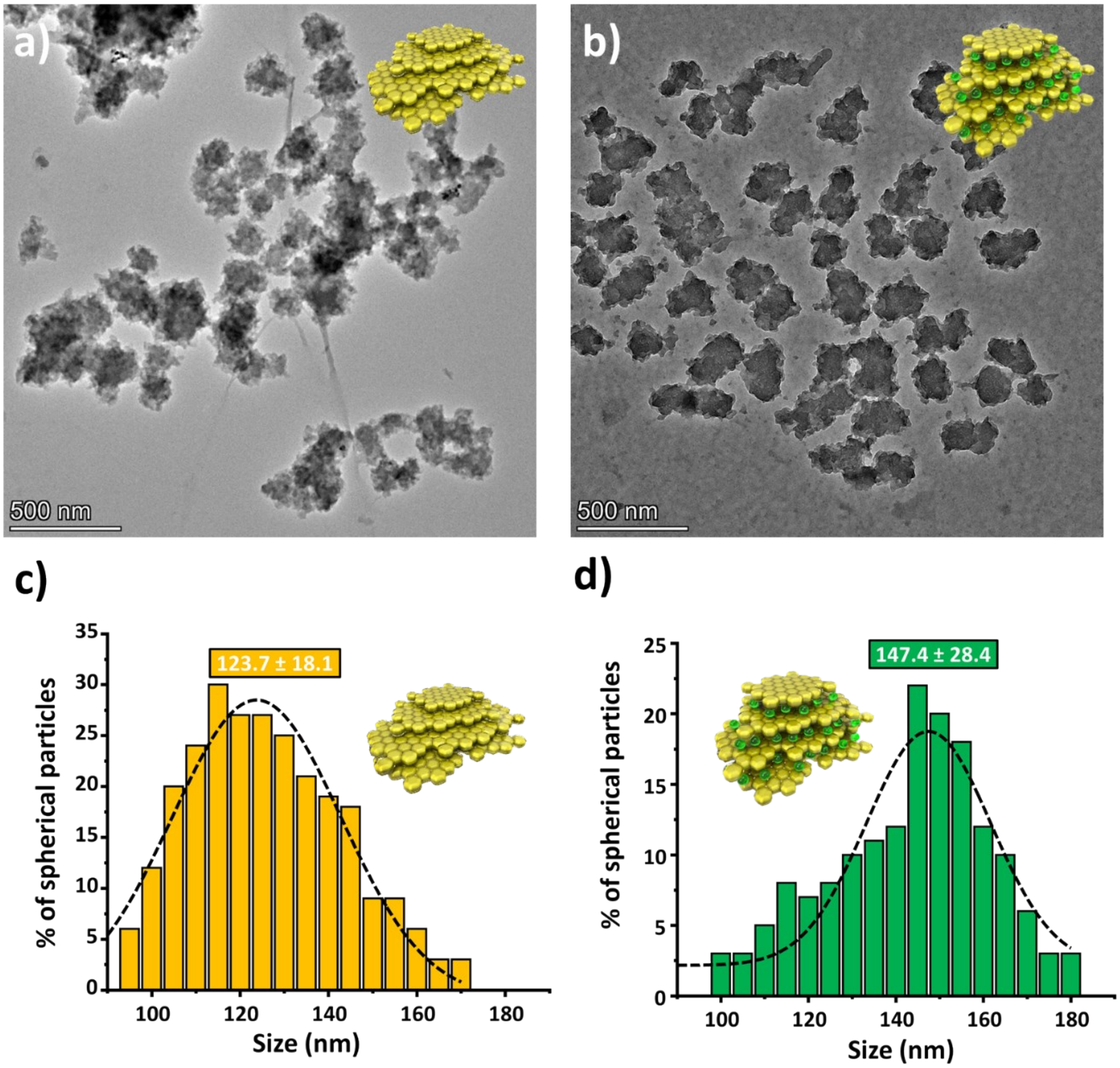

Figure S7. HR-TEM (a, b) images and size distributions (c, d) of TTA-DFP-nCOF (a, c) and TTA-DFP-nCOF/insulin (b, d). In order to estimate the average size of the particles, an average of 300 particles were counted.

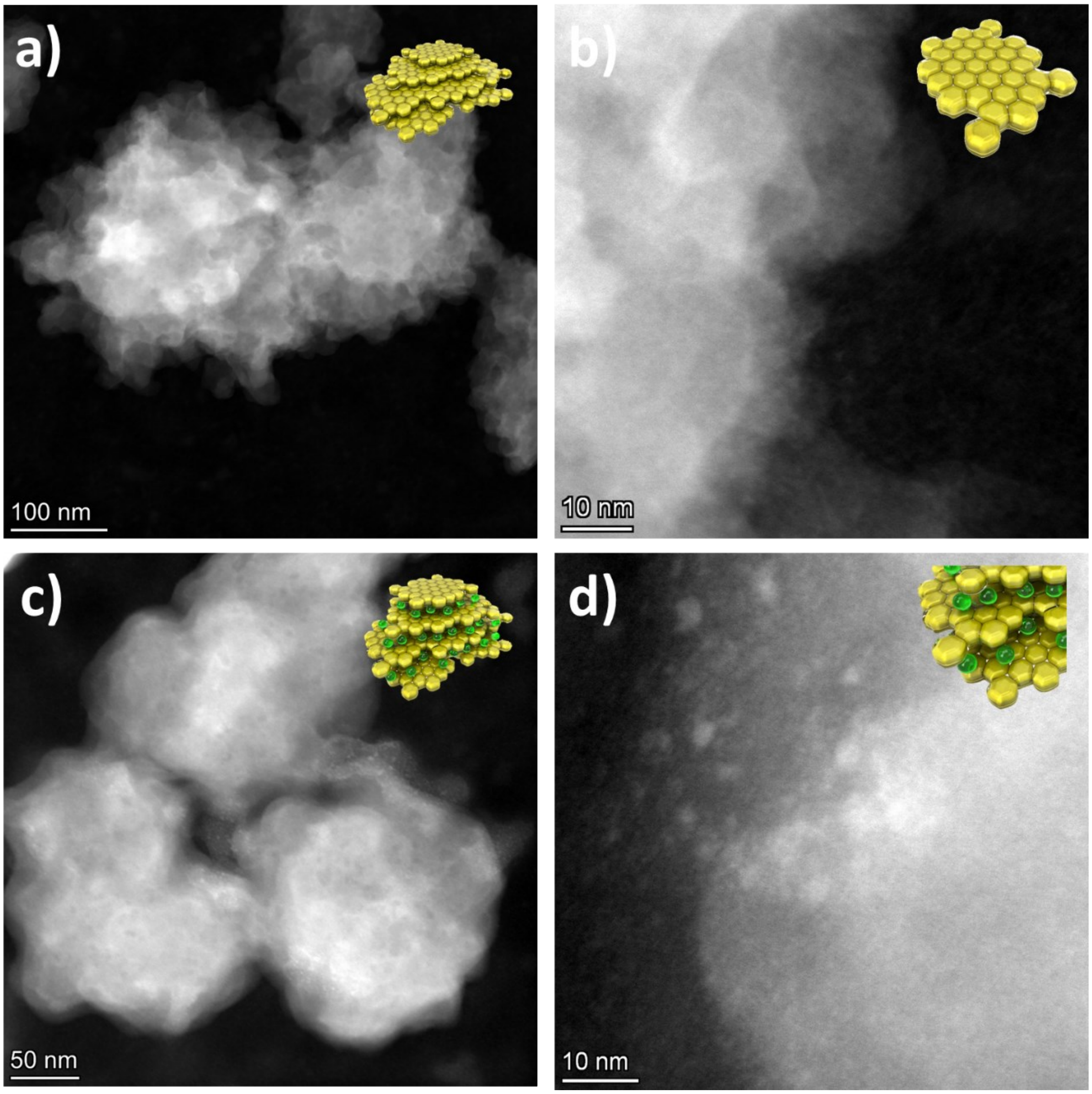

Figure S8. Comparison of STEM images for TTA-DFP-nCOF (a, b) and TTA-DFP-nCOF/insulin (c, d).

Elemental Mapping

The chemical mapping was carried out in STEM-EDAX mode wherein the energy-dispersive X-ray analysis (EDAX) was carried out using a super-X EDS detector. The system has superior sensitivity with resolution of ≤ 136eV@Mn-Kα for 10kcps at zero-degree sample tilt. The detector provides quick data even for low intensity EDS signals. The data is the sum of 4 detectors and the collection time for the elemental maps in fast mapping mode can be reduced to minutes from hrs. The data

was analyzed using Velox analytical software. The samples for the HRTEM study were prepared on holey carbon film mounted on a copper grid.

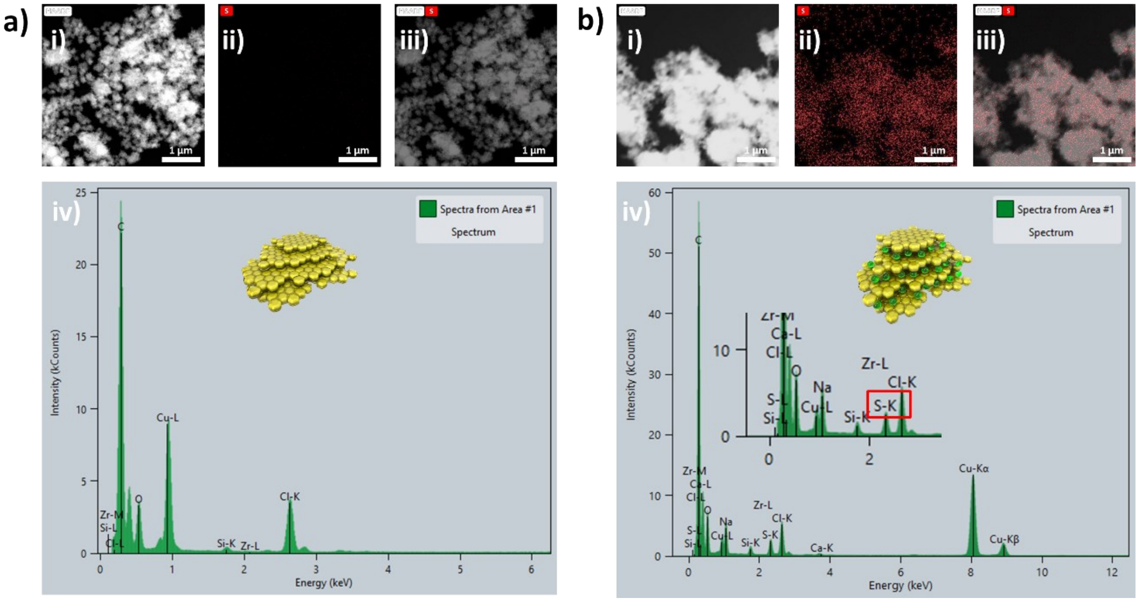

Figure S9. TEM mapping of sulfur element S in a) TTA-DFP-nCOF and b) TTA-DFP-nCOF/insulin. i) STEM image, ii) EDS mapping for S, iii) overlay of i) and ii) showing the localization of S elements in the nanoparticles. iv) Elemental analysis.

3.2. AFM Analysis

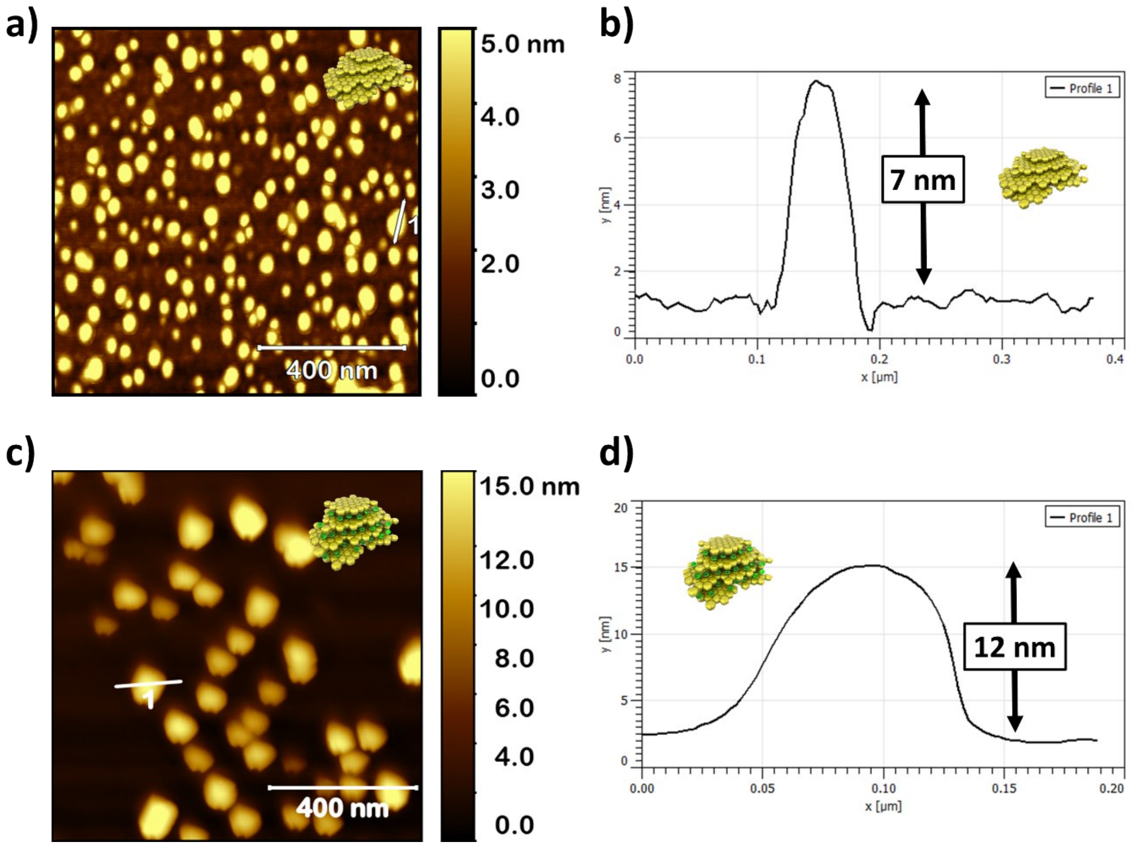

Figure S10. AFM images (a, c) and height profiles (b, d) of TTA-DFP-nCOFs (a, b) and TTA-DFP-nCOFs/Insulin (c, d).

3.3. Dynamic light scattering (DLS) characterization

DLS measurements were carried out on a Zetasizer Nano-ZS (Malvern Instruments) to determine the Zeta(ζ)-potential as well as the hydrodynamic size of the nanoparticles. All samples were analyzed at room temperature in 100 mM HEPES.

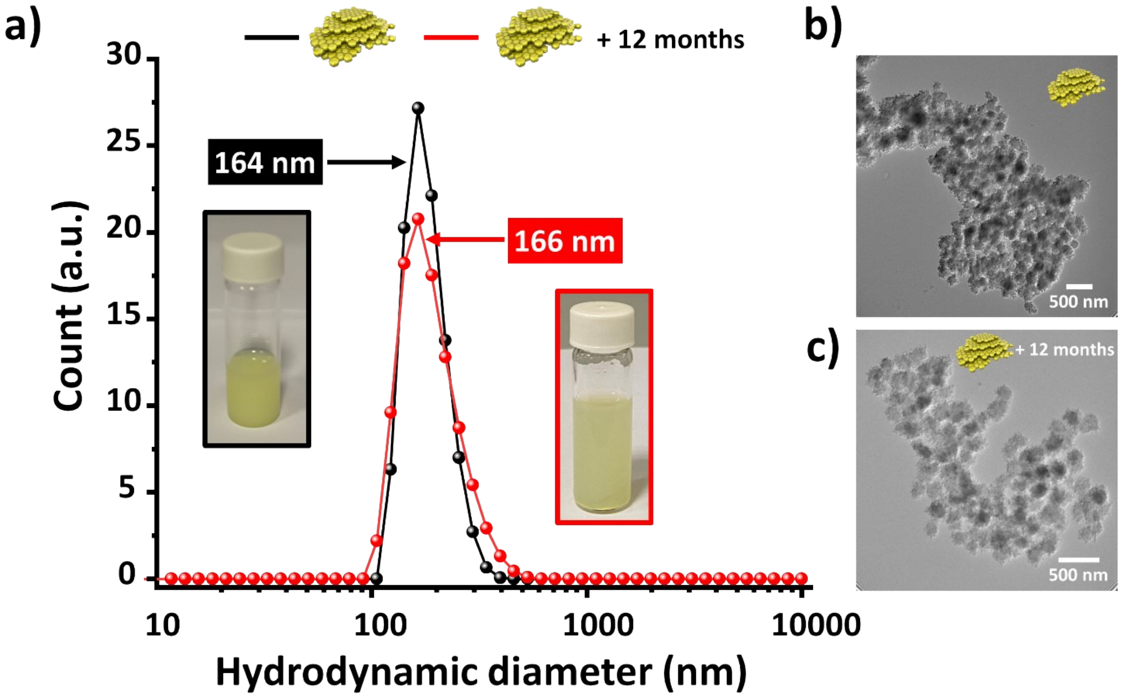

Figure S11. Hydrodynamic diameter (a) and TEM images (b, c) of TTA-DFP-nCOF after synthesis (black and b) and after 12 months (red and c) in 100 mM HEPES buffer at pH 7.4. Inset: pictures of the solutions at t = 0 and t = 12 months. The experiment was performed in triplicate.

3.4. Powder X-ray diffraction (PXRD) measurements

Powder X-ray diffraction (PXRD) measurements were carried out to confirm the crystalline nature of the framework. The TTA-DFP-nCOFs were found highly crystalline in nature. In fact, we observed a strong peak at 2θ of 4.9 ° assigned to the (110) plane of the regularly ordered lattice. TTA-DFP-nCOF shows a broad peak at ~24.80 corresponding to the reflection from the (003) plane.

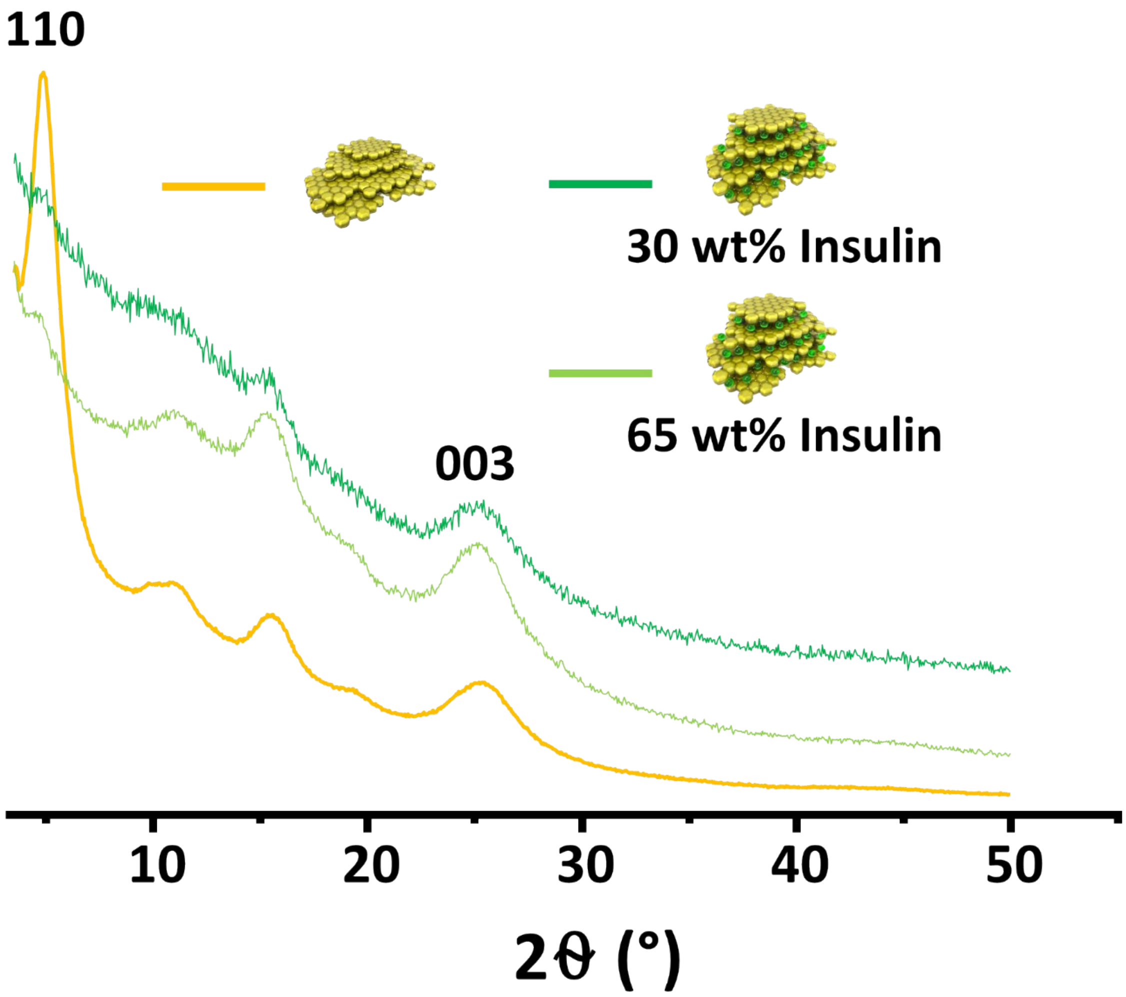

Figure S12. PXRD patterns of pristine TTA-DFP-nCOF (yellow) and TTA-DFP-nCOF/insulin (30 % loading capacity, dark green) and TTA-DFP-nCOF/insulin (65 % loading capacity, light green).

3.5. N_2 adsorption-desorption experiments

N_2 adsorption-desorption isotherms were obtained at 77 K using Micrometics ASAP 2020 surface area analyzer. Specific surface areas (S_{BET}) of the samples were calculated using Brunaur-Emmet-Teller (BET), whereas the pore volume (V_p) and pore size distribution (D_{BJH}) curves were obtained from Barrett-Joyner-Halenda (BJH) method.

Before measurements, the TTA-DFP-nCOF (empty or loaded with Insulin) was activated at 358 K for 24 h to remove the solvent and trapped gas. Based on the IUPAC classification system, TTA-

DFP-nCOF exhibited type-II isotherms, which are indicative of microporous materials. BET surface area was found to be 384.52 $m^2 g^{-1}$.

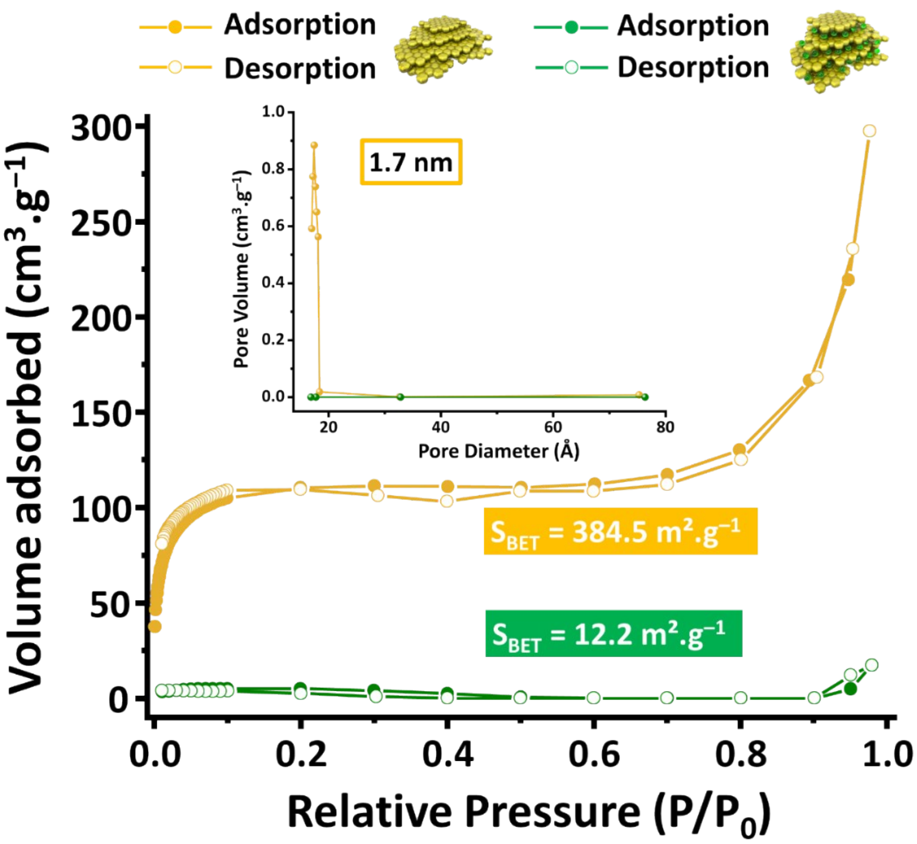

Figure S13. Nitrogen adsorption/desorption isotherms and pore size distribution curves (inset) at 77 K of TTA-DFP-nCOF before (yellow) and after loading with Insulin (green). The experiment was performed in triplicate.

3.6. Fourier Transform infrared (FTIR) spectroscopy

The TTA-DFP-nCOF formation as well as insulin loading was confirmed and characterized by ATR-IR spectroscopy using an Agilent Technologies Cary 600 Series FTIR spectrometer. The spectral data within the range of 4000 to 600 cm^{-1} were recorded, and 512 scans were averaged for each spectrum with a spectral resolution of 2 cm^{-1}. The spectrum of the background was recorded first and it was subtracted from the spectra of samples automatically.

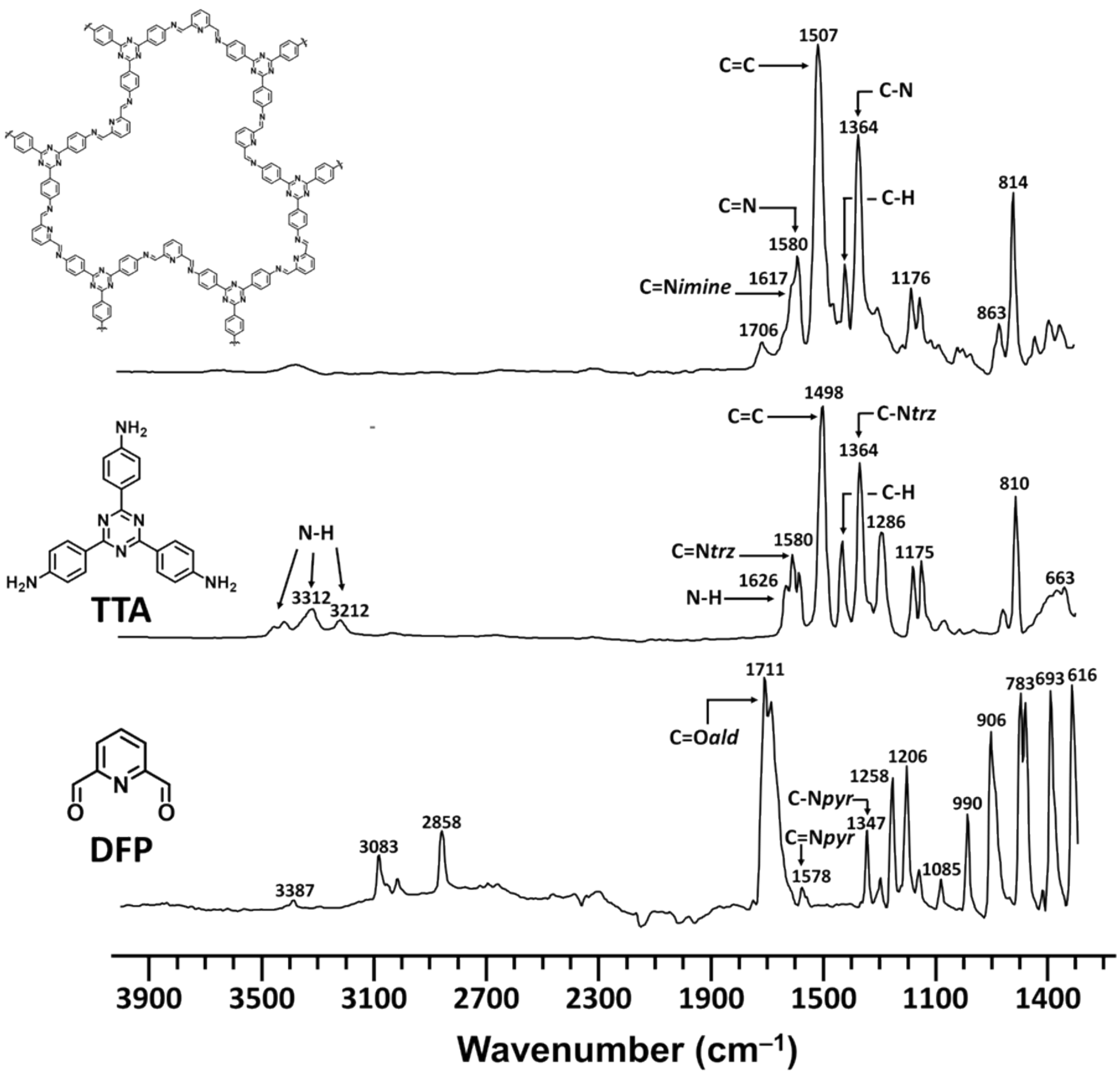

Figure S14. Stacked FTIR spectra of TTA-DFP-nCOF and its precursors, 2,6-diformylpyridine (DFP) and 4,4',4''-(1,3,5-triazine-2,4,6-triyl)trianiline (TTA). trz: triazine; ald: aldehyde; pyr: pyridine.

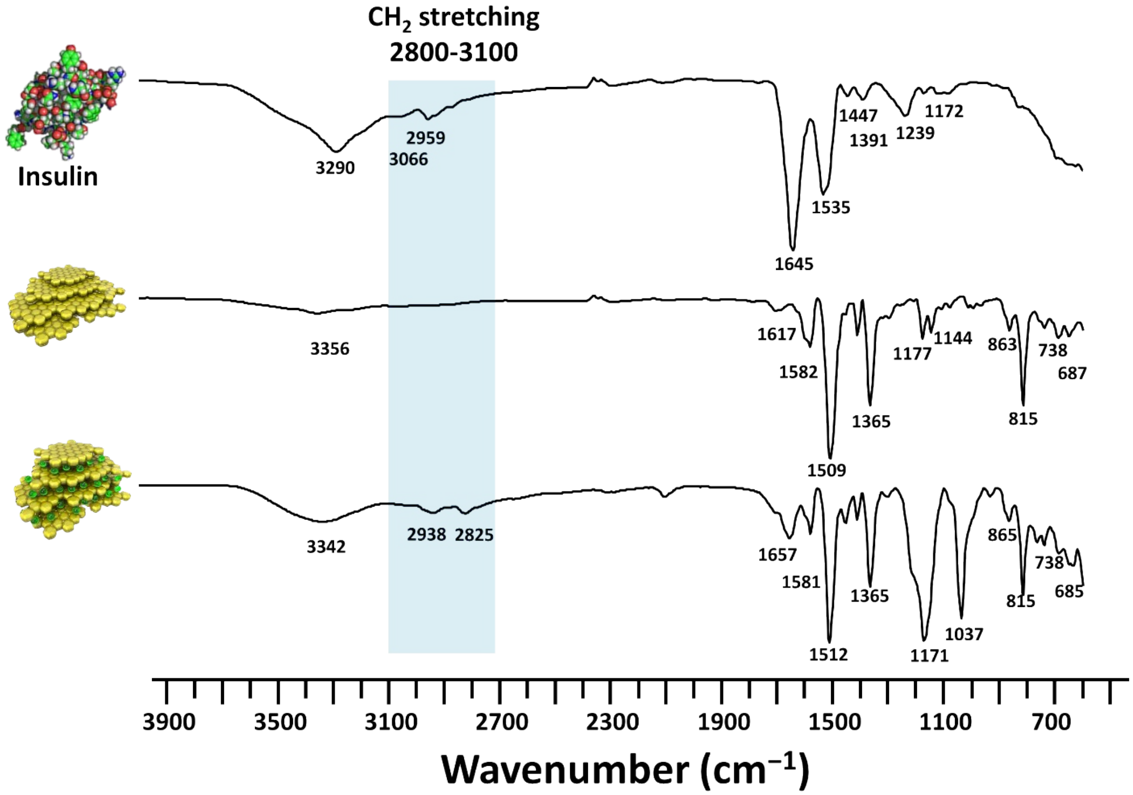

Figure S15. Stacked FTIR spectra of insulin (top), TTA-DFP-nCOF (middle) and TTA-DFP-nCOF/insulin (bottom).

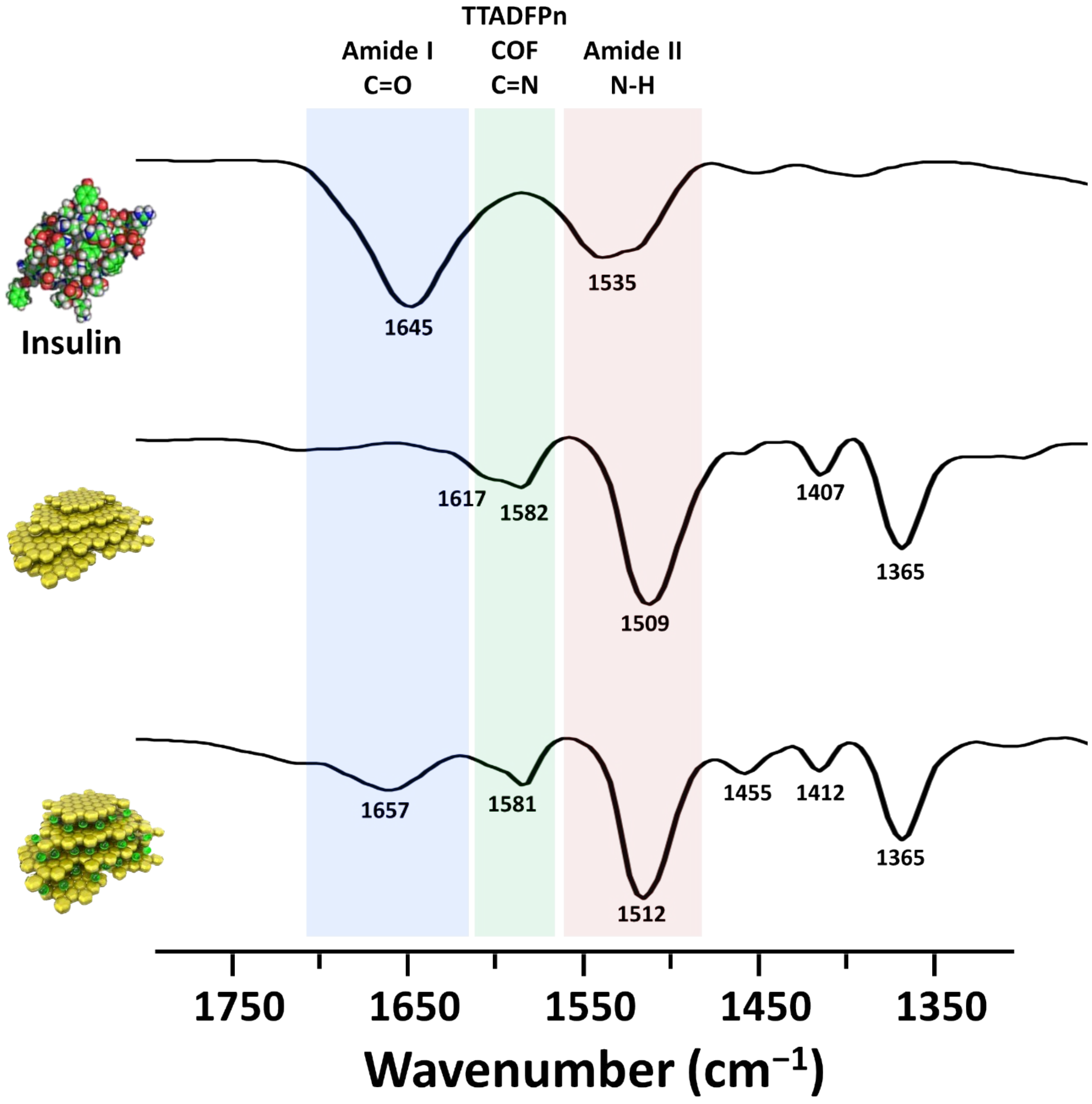

Figure S16. Comparison of the FTIR spectra of insulin (top), TTA-DFP-nCOF (middle) and TTA-DFP-nCOF/insulin (bottom) between 1750-1350 cm^{-1}.

3.7. TTA-DFP-nCOF characterization in stomach conditions (pH = 2)

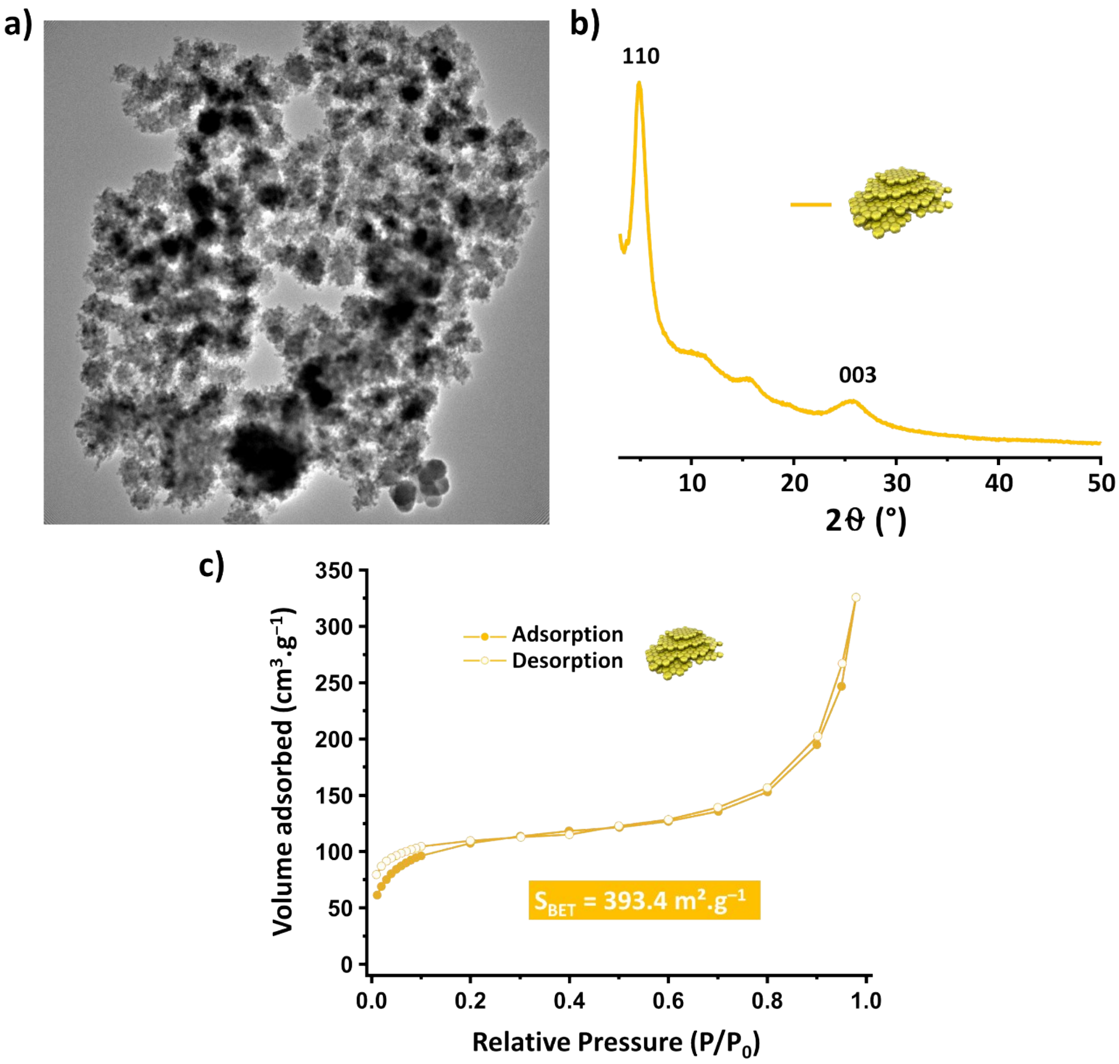

Figure S17. a) TEM image, b) PXRD pattern and c) nitrogen adsorption/desorption isotherms of TTA-DFP-nCOF suspended for 24 hours at pH = 2.0 showing no alteration of the nCOF structure.

3.8. ¹H NMR spectroscopy

¹H NMR spectroscopy was used to observe the uptake of insulin by TTA-DFP-nCOF.

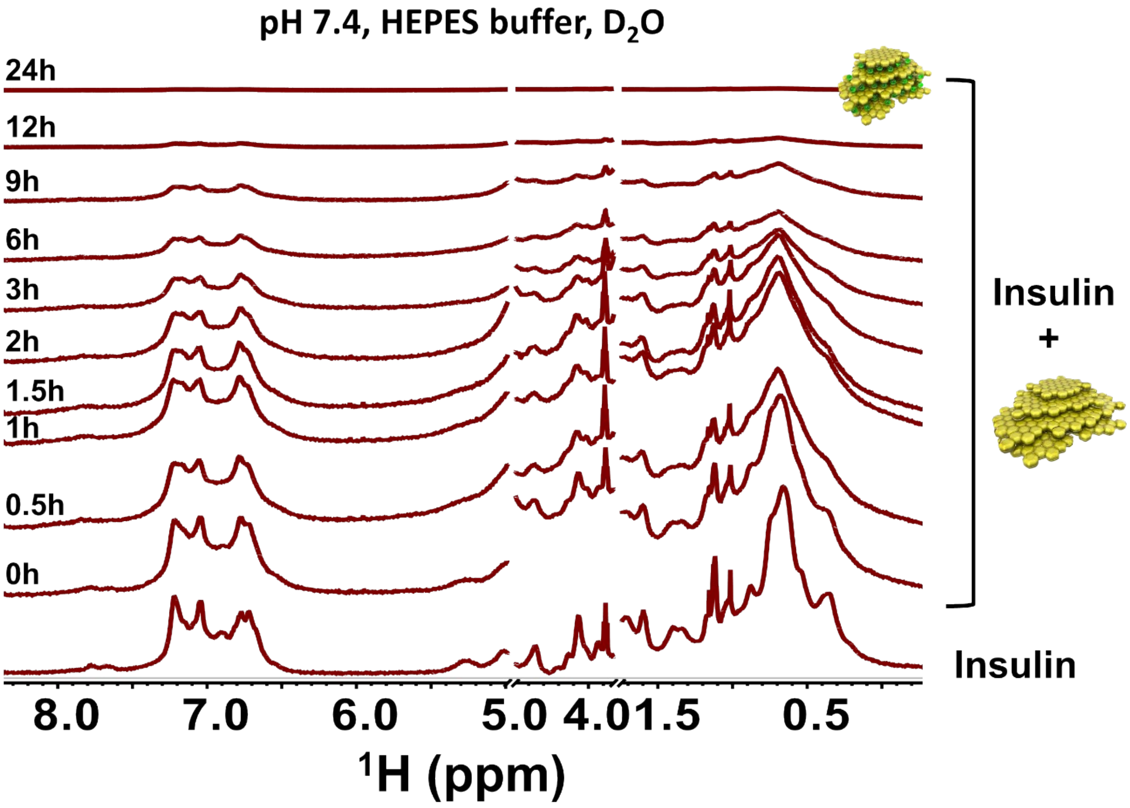

Figure S18. Monitoring insulin uptake into TTA-DFP-nCOF by ¹H NMR at regular time intervals. Stacked ¹H NMR spectra of insulin in the absence of TTA-DFP-nCOF (pH adjusted to 7.4 with an initial concentration of insulin of 10 mM. bottom trace), and in the presence of TTA-DFP-nCOF at t = 0, 30 min, 1h, 1.5h, 2h, 3h, 6h, 9h, 12h, and 24h in deuterated HEPES buffer solution in 500 MHz at 310 K.

3.9. Determination of the amount of insulin-FITC loaded in TTA-DFP-nCOF using fluorescence spectroscopy

At 37 °C, pH 7.4 and λ_{ex} = 488 nm, TTA-DFP-nCOF are not intrinsically fluorescent. This indicates that fluorescence quenching occurs within the TTA-DFP-nCOF construct. Such quenching can be attributed to electronic interactions between the excited Insulin-FITC and the TTA-DFP-nCOF, or to self-quenching of the dye in the nanoparticles where the effective concentration of the protein is relatively high. [33, 34] The intensity of solution fluorescence was measured in comparison to a calibration curve (Figure S19).

To estimate the amount of Insulin-FITC loading into TTA-DFP-nCOF, the unloaded insulin-FITC in the supernatant was determined by fluorescence spectroscopy based on comparison to a calibration curve of insulin standard solution.

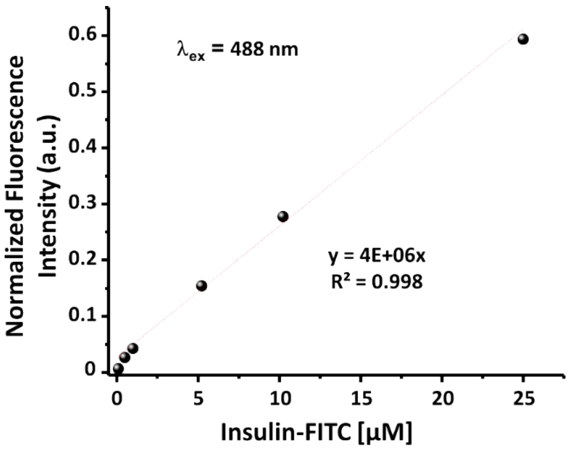

Figure S19. Calibration curve obtained by measuring the maximum fluorescence signal at different insulin-FITC concentrations (λ_{ex}= 488 nm, λ_{max} = 520 nm, $H_2 0$, 298 K).

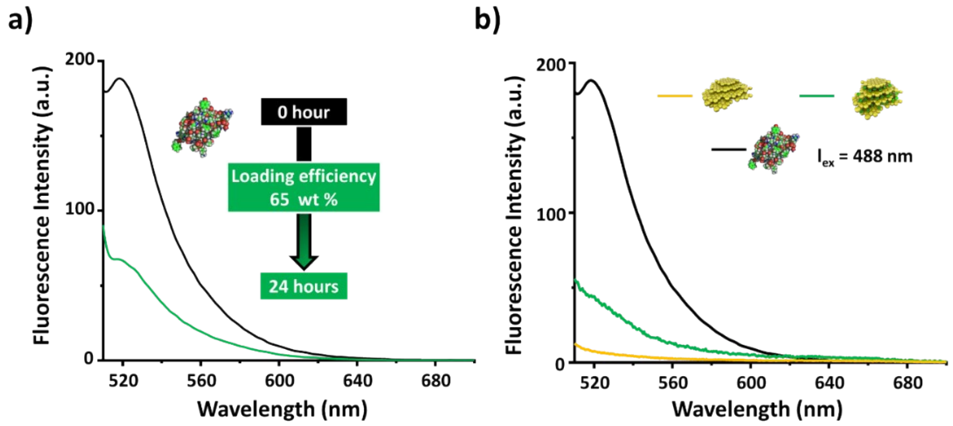

Figure S20. Fluorescence emission spectra of a) diluted supernatant solution of TTA-DFP-nCOF impregnated with insulin-FITC at t = 0 hour (black) and after 24 hours (green curve); b) TTA-DFP-nCOF (yellow), TTA-DFP-nCOF/insulin-FITC (green) and Insulin-FITC (black). λ_{ex}= 488 nm, H_2O at pH 7.4, 298 K. The experiment was performed in triplicate.

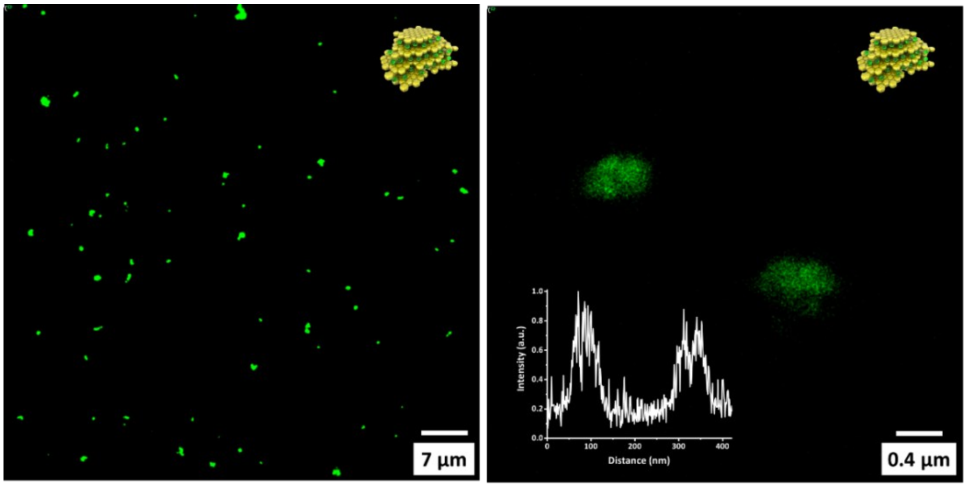

Figure S21. Confocal microscopy images of drop-cast TTA-DFP-nCOF/insulin-FITC on a cover slip to ensure immobilization of the NPs (λ_{ex} = 488 nm). The experiment was performed in triplicate.

3.10. DLS and Zeta(ζ)-potential characterization of TTA-DFP-nCOF/insulin

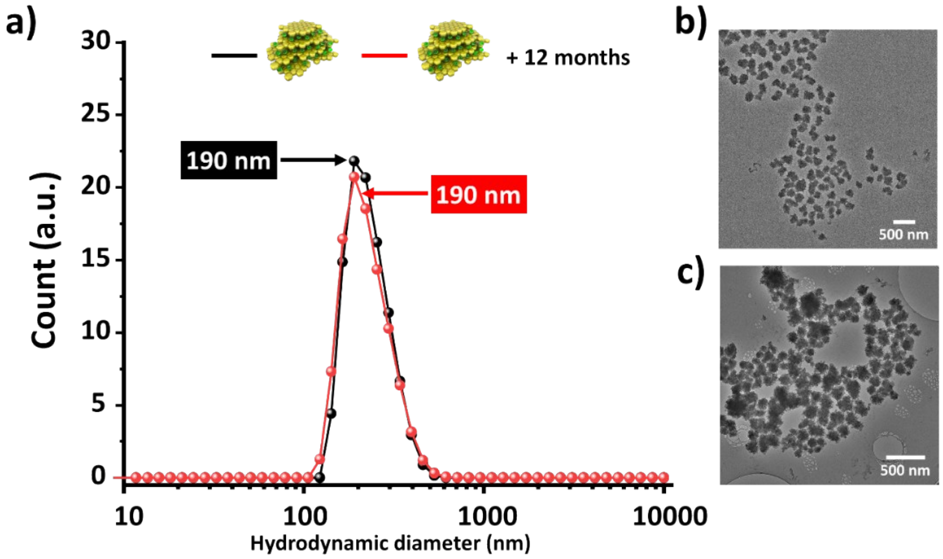

Figure S22. Hydrodynamic diameter (a) and TEM images (b, c) of TTA-DFP-nCOF/insulin after synthesis (black and b) and after 12 months (red and c) in 100 mM HEPES buffer at pH 7.4. The experiment was performed in triplicate.

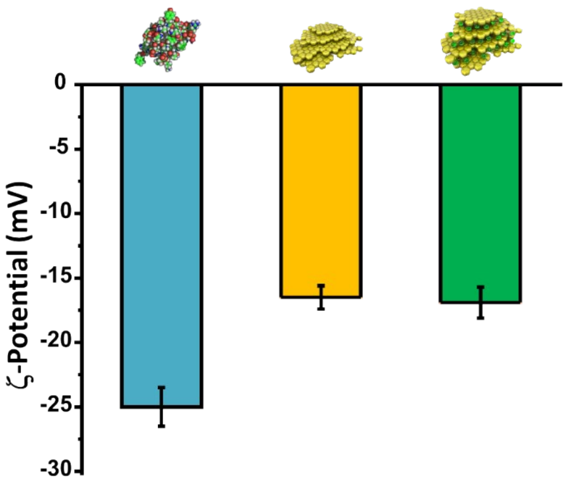

Figure S23. Zeta(ζ)-potential of insulin (blue), TTA-DFP-nCOF (yellow), and TTA-DFP-nCOF/insulin (green) at pH 7.4 in 100 mM HEPES. Error bars represent standard deviations of triplicate measurements.

3.11. X-ray photoelectron (XPS) spectroscopy

X-ray photoelectron spectroscopy (XPS) analysis was performed in order to analyse the elemental composition of TTA-DFP-nCOF before and after addition of insulin, as well as to understand the interactions occurring between the nCOF surface and the protein. XPS experiments were carried out on a Kratos Axis Ultra DLD spectrometer under a base pressure of ∼ 2×10^{-10} mbar. A monochromated Al Kα X-ray source (1486.69 eV) was used to irradiate samples at room temperature. XPS spectra were recorded from an analysis area of 700 µm × 300 µm. High-resolution XPS data of core levels was obtained with an energy resolution of 0.05 eV. For consistency, XPS measurements were calibrated to C1s (∼ 285 eV). Data was analyzed using CasaXPS package with Shirley background subtraction.

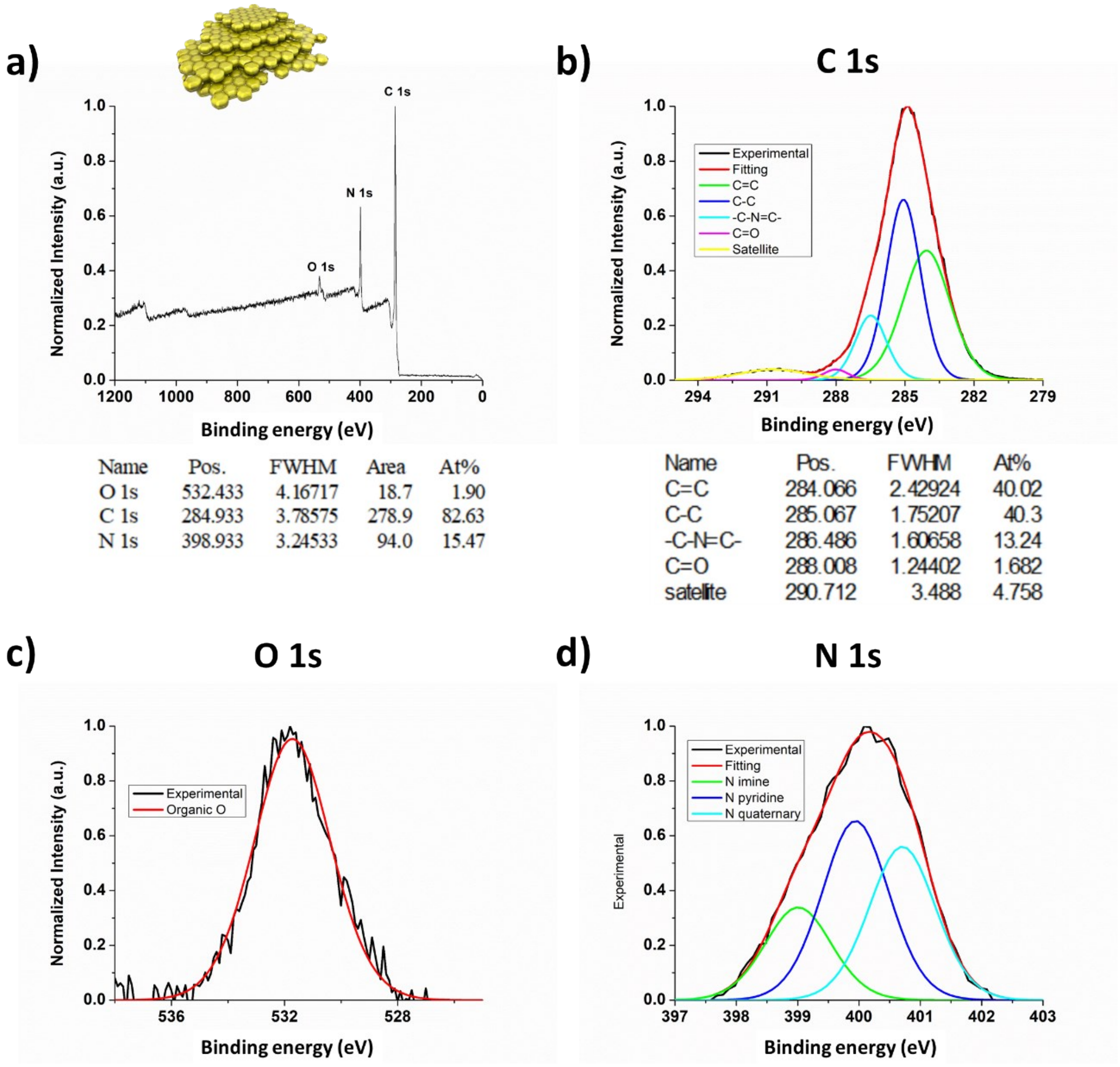

a)

Name	Pos.	FWHM	Area	At%
O 1s	532.433	4.16717	18.7	1.90
C 1s	284.933	3.78575	278.9	82.63
N 1s	398.933	3.24533	94.0	15.47

b)

Name	Pos.	FWHM	At%
C=C	284.066	2.42924	40.02
C-C	285.067	1.75207	40.3
-C-N=C-	286.486	1.60658	13.24
C=O	288.008	1.24402	1.682
satellite	290.712	3.488	4.758

c)

Name	Pos.	Area	At%
O 1s	531.806	21.3	100
Organic O	531.727	21.15	100

d)

Name	Pos.	Area	At%
N imine	399.009	9.80	10.92
N pyridine	399.943	18.94	21.12
N quaternary	400.707	16.22	18.08

Figure S24. High resolution XPS spectra of insulin. (a) XPS survey spectrum, and binding energy spectrum for (b) C1s, (c) O1s, (d) N 1s and (e) S 2s.

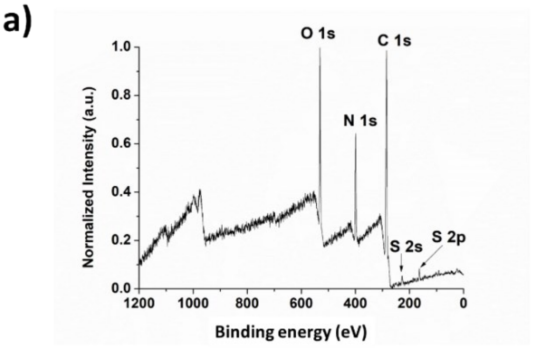

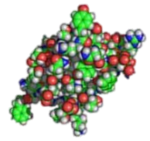

Insulin

Name	Pos.	FWHM	Area	At%
O 1s	530.897	3.09002	87.7	16.12
C 1s	284.897	4.24569	128.2	69.04
N 1s	399.397	3.13296	46.1	13.78
S 2p	163.397	2.08544	3.2907	1.05

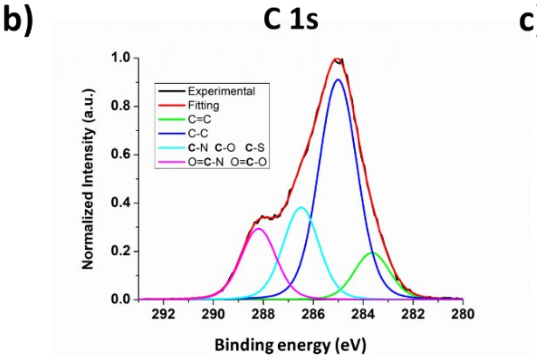

Name	Pos.	Area	At%
C=C	283.689	12.96	12.35
C-C	285.01	54.21	51.64
C-N C-O C-S	286.482	21.67	20.64
O=C-N O=C-O	288.182	16.14	15.37

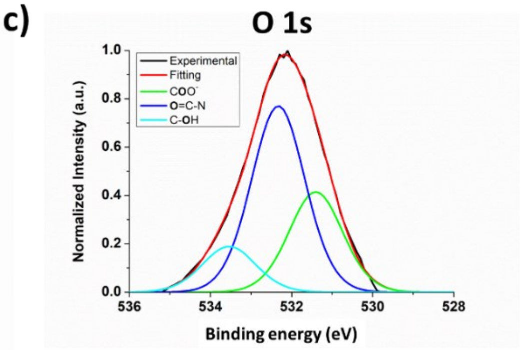

Name	Pos.	Area	At%
COO	531.404	21.05	30.18
O=C-N	532.33	39.09	56.05
C-OH	533.543	9.60	13.76

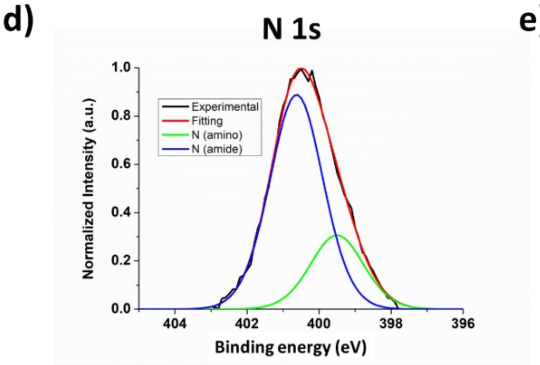

Name	Pos.	Area	At%
N (amine)	399.502	9.95	25.65
N (amide)	400.625	28.84	74.35

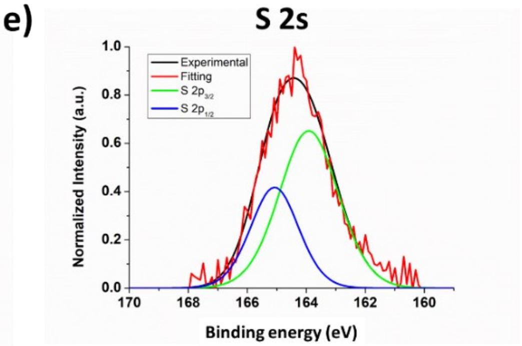

Name	Pos.	Area	At%
S 2p3/2	163.918	2.66	65.78
S 2p1/2	165.078	1.38	34.22

Figure S25. High resolution XPS spectra of TTA-DFP-nCOF. (a) XPS survey spectrum, and binding energy spectrum for (b) C1s, (c) O1s and (d) N 1s.

a)

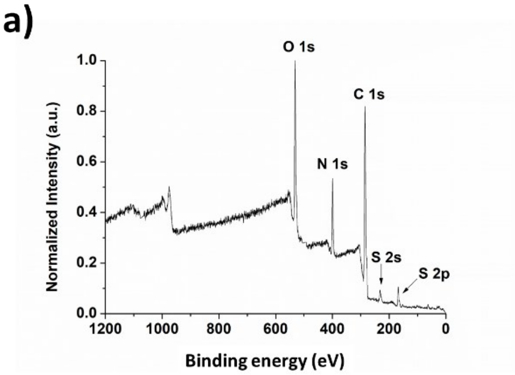

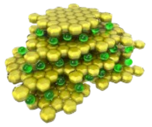

Name	Pos.	FWHM	Area	At%
O 1s	531.144	3.51672	150.0	20.23
C 1s	285.144	4.17036	163.4	64.60
N 1s	399.144	3.17232	52.3	11.48
S 2p	168.144	3.39329	15.6	3.68

b) **C 1s**

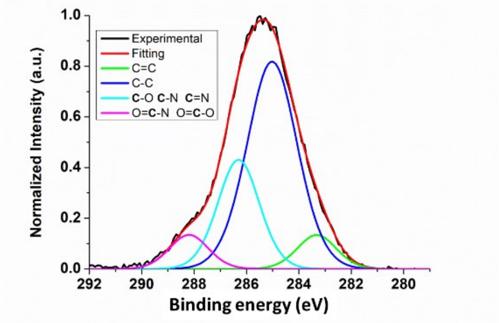

Name	Pos.	FWHM	L.Sh.	Area	At%
C=C	283.319	1.75572	GL(30)	6.91	7.532
C-C	285.024	2.25557	GL(30)	54.17	59.09
C-O C-N C=N	286.306	1.86982	GL(30)	23.68	25.83
O=C-N O=C-O	288.199	1.7474	GL(30)	6.93	7.554

c) **O 1s**

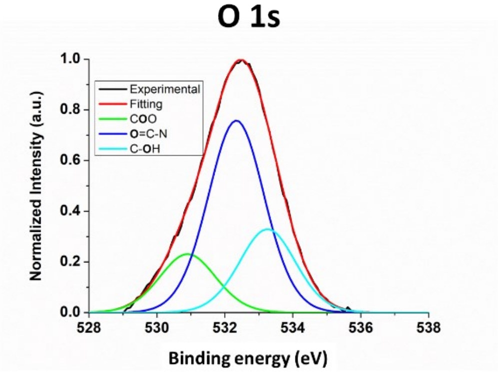

Name	Pos.	L.Sh.	Area	At%
COO	530.904	GL(30)	14.99	17.52
O=C-N	532.335	GL(30)	49.21	57.5
C-OH	533.254	GL(30)	21.38	24.98

d) **N 1s**

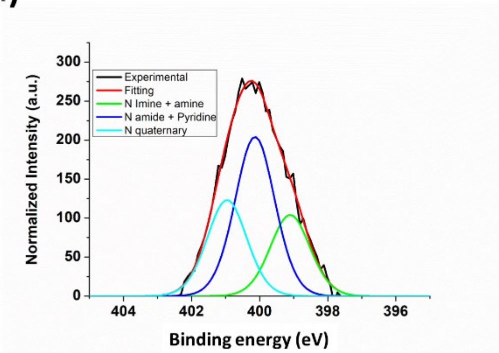

Name	Pos.	Area	At%
N imine + amine	399.105	5.66	24.12
N amide + N Pyridine	400.138	11.10	47.32
N quaternary	400.96	6.70	28.56

e) **S 2s**

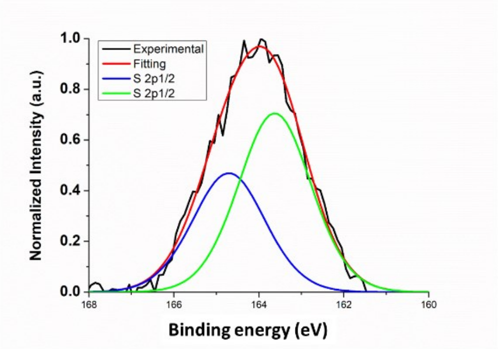

Name	Pos.	Area	At%
S 2p3/2	163.626	3.57	60.05
S 2p 1/2	164.708	2.38	39.95

Figure S26. High resolution XPS spectra of TTA-DFP-nCOF/insulin. (a) XPS survey spectrum, and binding energy spectrum for (b) C1s, (c) O1s, (d) N 1s and (e) S 2s.

4. pH- and glucose dependent Insulin-FITC Release from TTA-DFP-nCOF by fluorescence emission spectroscopy

4.1. Insulin release conditions

The effect of pH on the release of Insulin-FITC from TTA-DFP-nCOF was monitored over time in water buffered with PBS (10 mM) at 37 °C and pH =2.0 and 7.4. The pH of the solutions was adjusted using a 1 M HCl(aq) solution.
The effect of the glucose concentration on the release of insulin-FITC from TTA-DFP-nCOF was monitored using fluorescence spectroscopy over time in water buffered with PBS (10 mM) at 37 °C and in several glucose concentrations ([glucose] = 0, 1 3, and 5 mg.mL^{-1}).

The effect of human serum, a mix of 11 amino acids (alanine, arginine, asparagine, aspartate, cysteine, glutamate, glutamine, glycine, proline, serine, tyrosine), a saline solution of fructose (3 mg mL^{-1}) or sucrose (3 mg mL^{-1}) on the release of insulin-FITC from TTA-DFP-nCOF was monitored over time in water buffered with PBS (10 mM) at 37 °C and in hyperglycemic conditions ([glucose] = 3 mg.mL^{-1}).

At regular intervals, samples were withdrawn and the fluorescence intensity was measured.

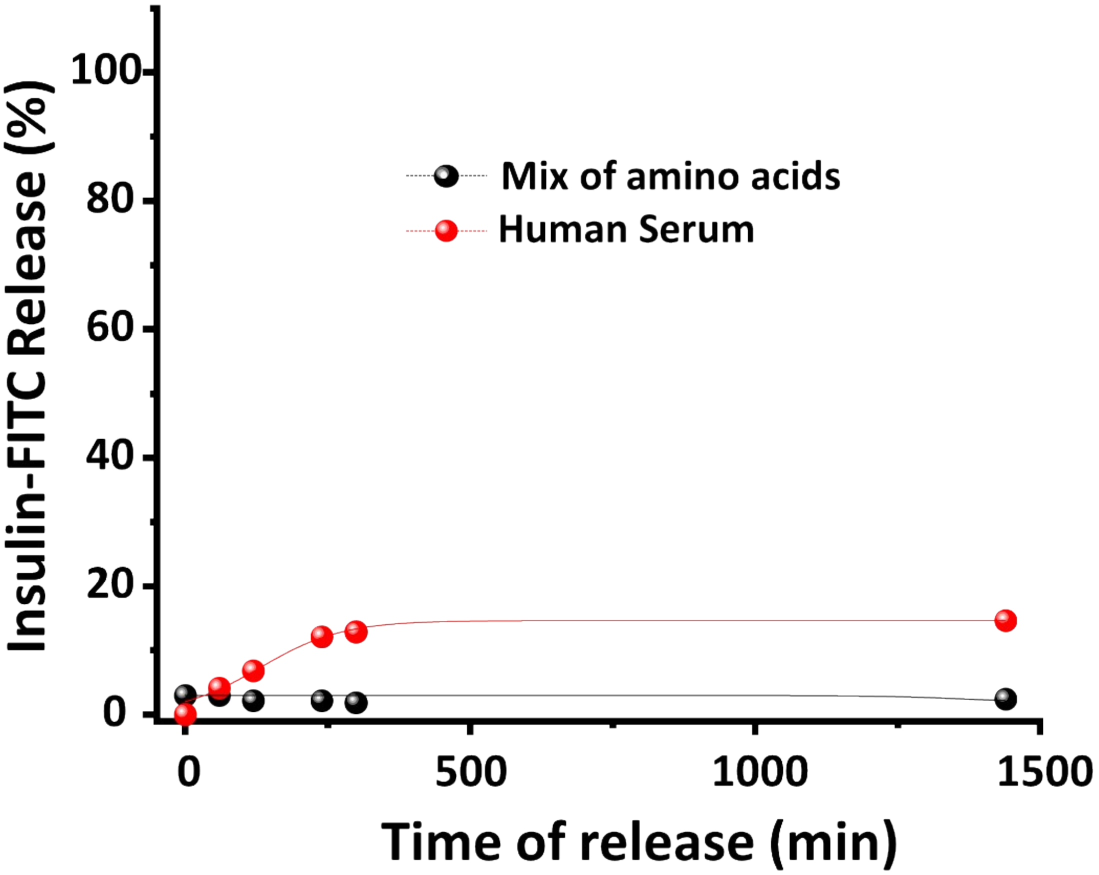

Figure S27. *In vitro* accumulated insulin-FITC release from the TTA-DFP-nCOF/insulin at 37 °C in human serum (red) and a mix of amino acids (black) for 24 hours. The % of drug released was measured using fluorescence emission. The experiment was performed in triplicate.

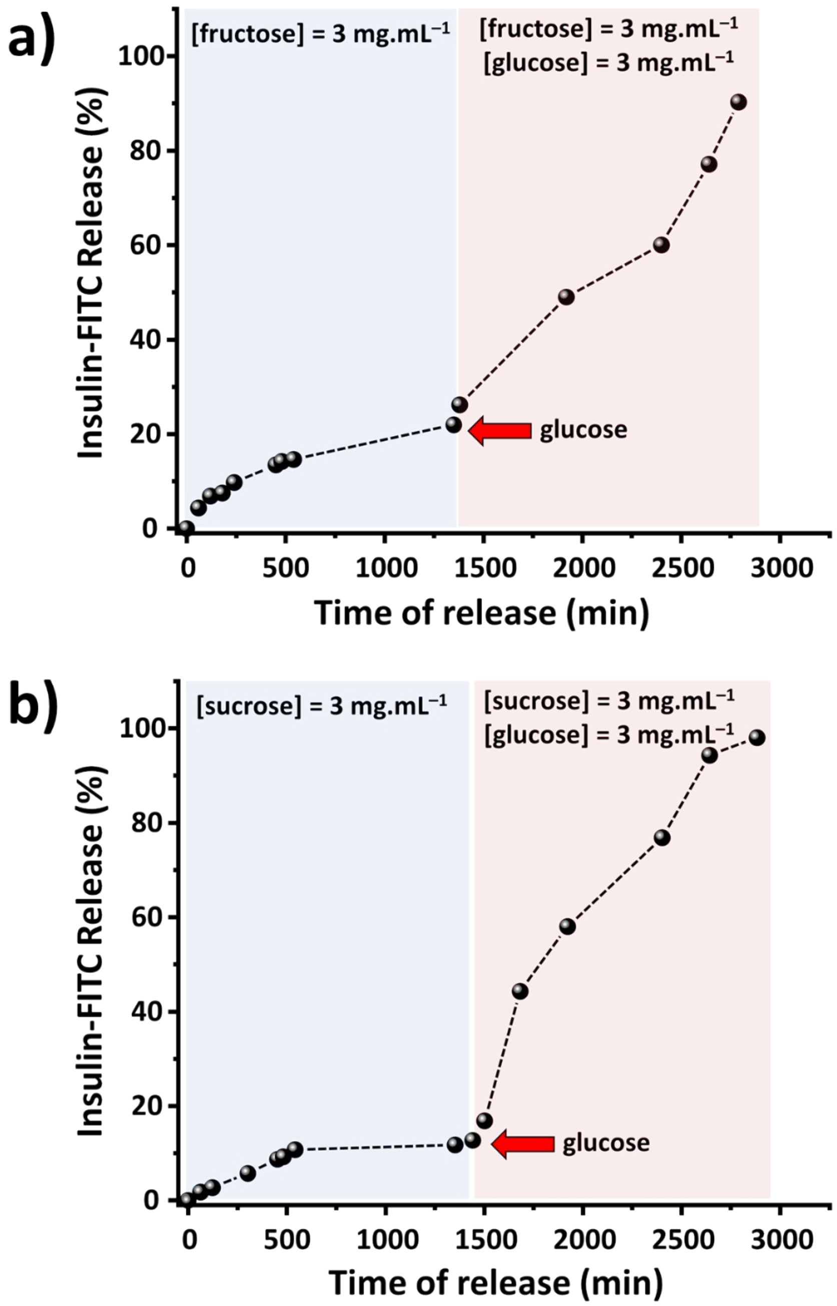

Figure S28. *In vitro* accumulated insulin-FITC release from the TTA-DFP-nCOF/insulin at 37 °C in PBS containing a) fructose ([fructose] = 3 mg.mL^{-1}) and b) sucrose ([sucrose] = 3 mg.mL^{-1}) for 24 hours then glucose was added ([glucose] = 3 mg.mL^{-1}) to trigger insulin-FITC release. The % of drug released was measured using fluorescence emission. The experiment was performed in triplicate.

4.2. Circular dichroism (CD) spectroscopy

To evaluate the changes of the activity and structure of insulin released from the nanoparticles, circular dichroism (CD) spectroscopy was performed as a common method to analyze the secondary structure of a protein with high reliability. In the CD spectra of the native insulin in HEPES (pH 7.4), there were two extrema at 208 and 222 nm, due to the α-helix structure and β-structure, respectively.

We evaluated the chemical stability of insulin loaded in TTA-DFP-nCOF exposed to the GI fluid simulations (pH 2.0, 24 hours). At acidic pH, insulin is not released from the nanoparticle, therefore in order to perform CD analysis we exposed TTA-DFP-nCOF/insulin to NaOH (0.1M) to release the protein from the NPs. As presented in Figure S29 far-UV CD spectroscopy of the insulin released from NPs and pure insulin solution showed two negative bands at 208 nm and 222 nm which correspond to the predominant α-helix structure and β-pleated sheet structure, respectively. The ratio of intensity of 208 and 223 nm bands ([Φ]208/[Φ]223) has usually been employed to provide a qualitative measure of insulin association. The [Φ]208/[Φ]223 ratios of native and GI fluid exposed insulin from TTA-DFP-nCOF were both 1.2 which reflected that there was no significant difference in the secondary structure between the native and GI fluid exposed TTA-DFP-nCOF/insulin.

The CD spectrum of the insulin after releasing for 12 hours from the TTA-DFP-nCOF/insulin under the hyperglycemic environment was similar to that of the native insulin with two extrema (Figure S30). The ratios between bands ([ϕ]208/[ϕ]222) for the native and released insulin were 1.25 and 1.24, respectively. Therefore, the secondary structure of the insulin released from the nanoparticles was similar to the original insulin. Accordingly, the released insulin maintained its structure and properties.

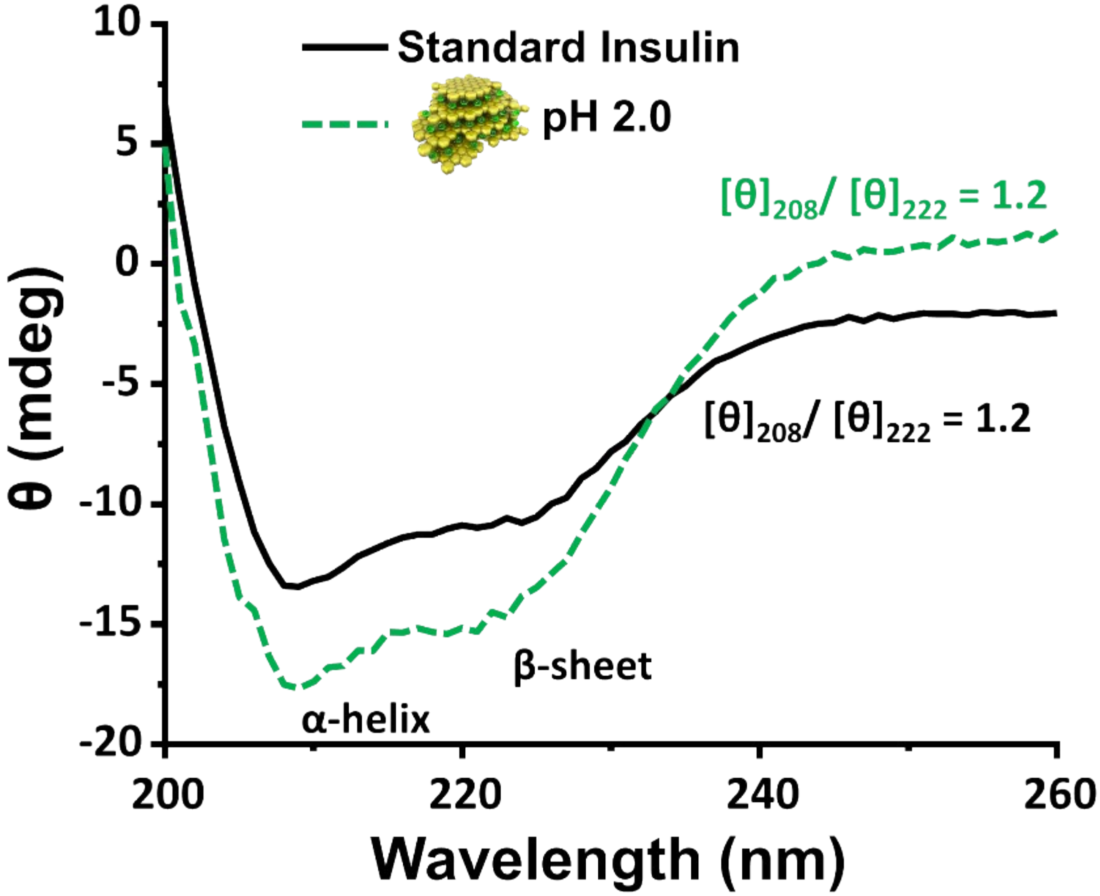

Figure S29. Circular dichroism spectra of native insulin solution (black) and insulin incubated in gastro-intestinal environment (green). Deg = degree. The experiment was performed in triplicate.

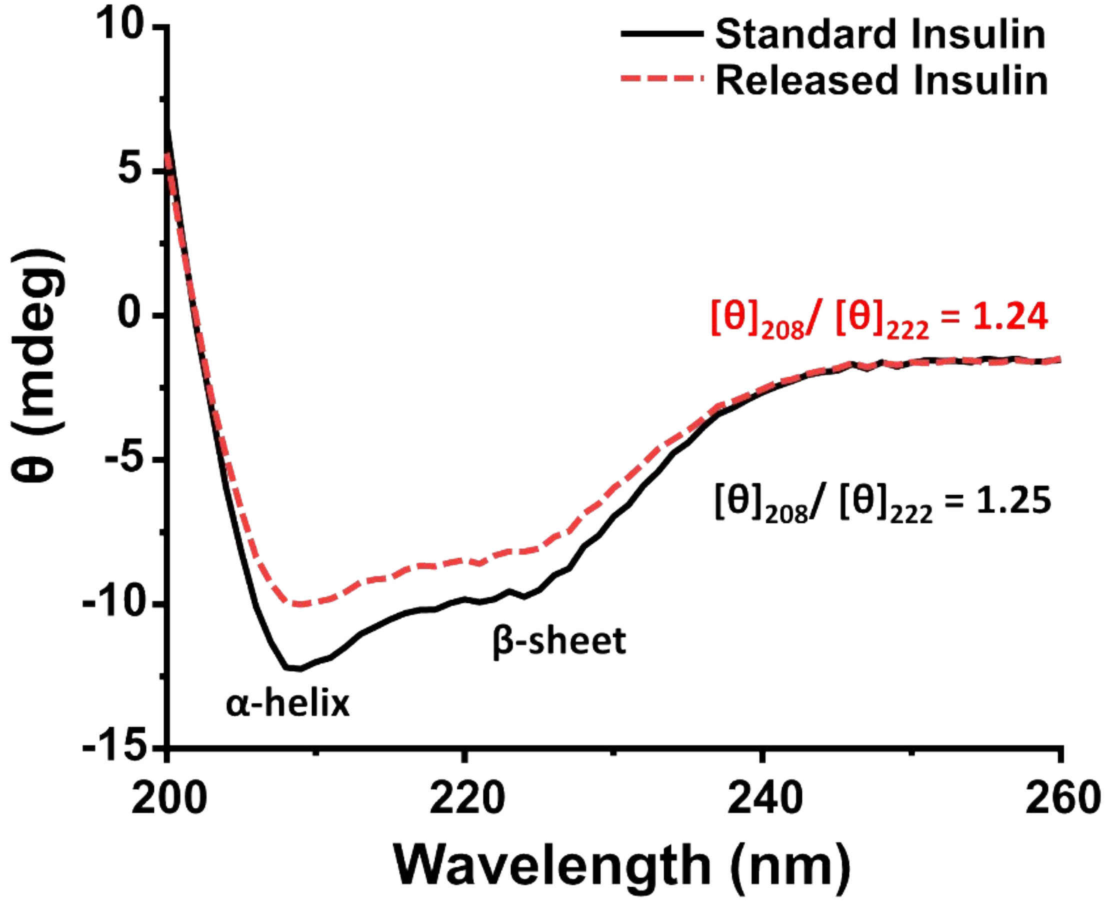

Figure S30. Circular dichroism spectra of native insulin solution (black) and insulin released from the TTA-DFP-nCOF/insulin (red) incubated in hyperglycemic conditions (5 mg.mL^{-1}) for 12 hours. Deg = degree. The experiment was performed in triplicate.

4.3 Stability study of TTA-DFP-nCOF/insulin

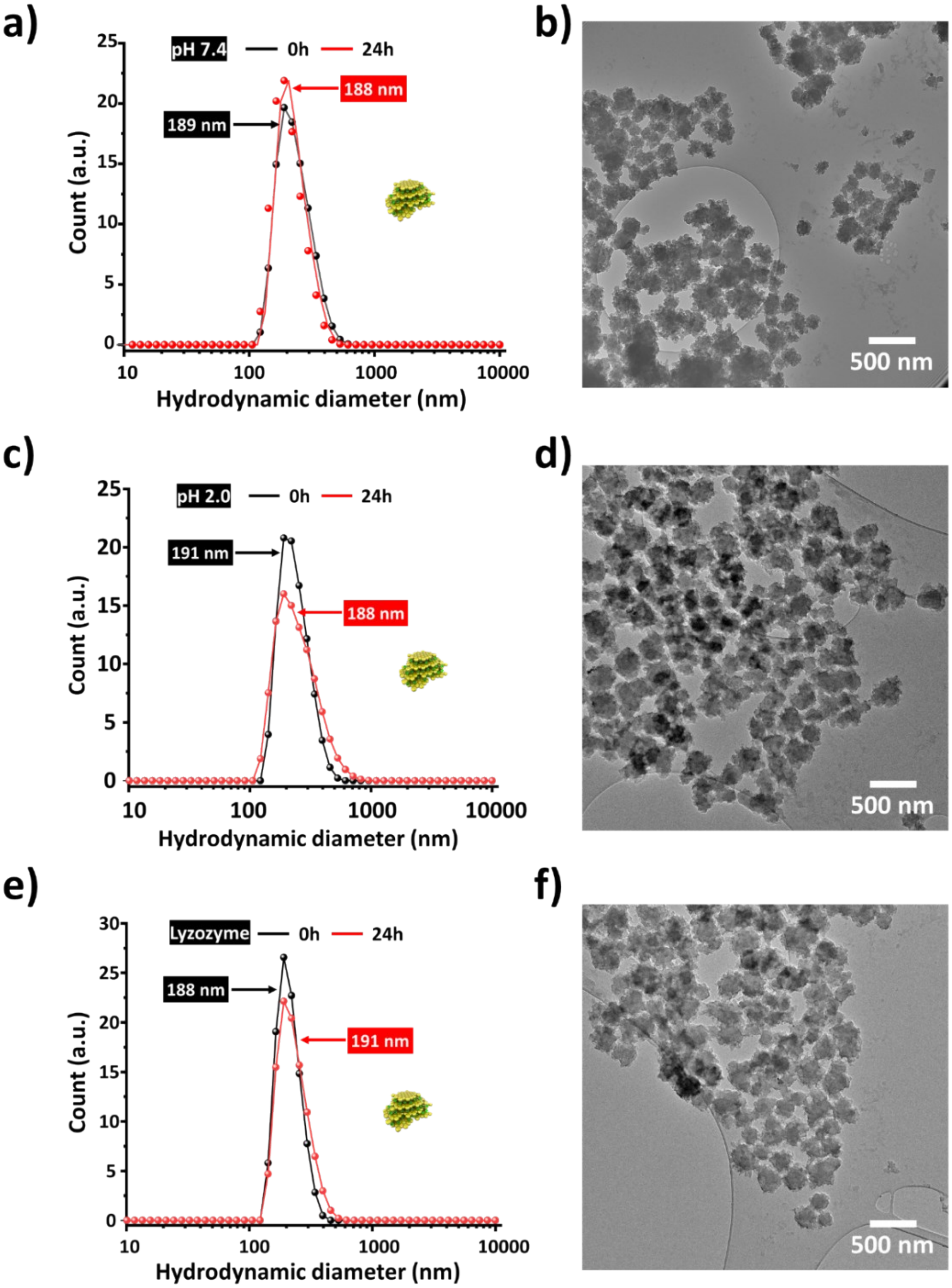

Figure S31. Hydrodynamic diameter (a, c and e) and TEM images (b, d and f) of TTA-DFP-nCOF/insulin in 100 mM HEPES buffer at pH 7.4 (a, b), pH 2.0 (c, d) and in presence of lysozyme (5 mg.mL^{-1}, e, f) at t = 0 hour (black) and 24 hours (red). The experiment was performed in triplicate.

4.4. Glucose interaction

In order to study the release mechanism of insulin triggered by glucose, we incubated the TTA–DFP-nCOF 24 hours and 37 °C with i) insulin alone, ii) glucose alone (5 mg.mL^{-1}) and iii) insulin followed by 24 hours with glucose (5 mg.mL^{-1}). Samples were washed thoroughly and freeze-dried using lyophilization. Loading efficiency (wt%) was calculated using mass differences by comparing with the TTA–DFP-nCOF mass.

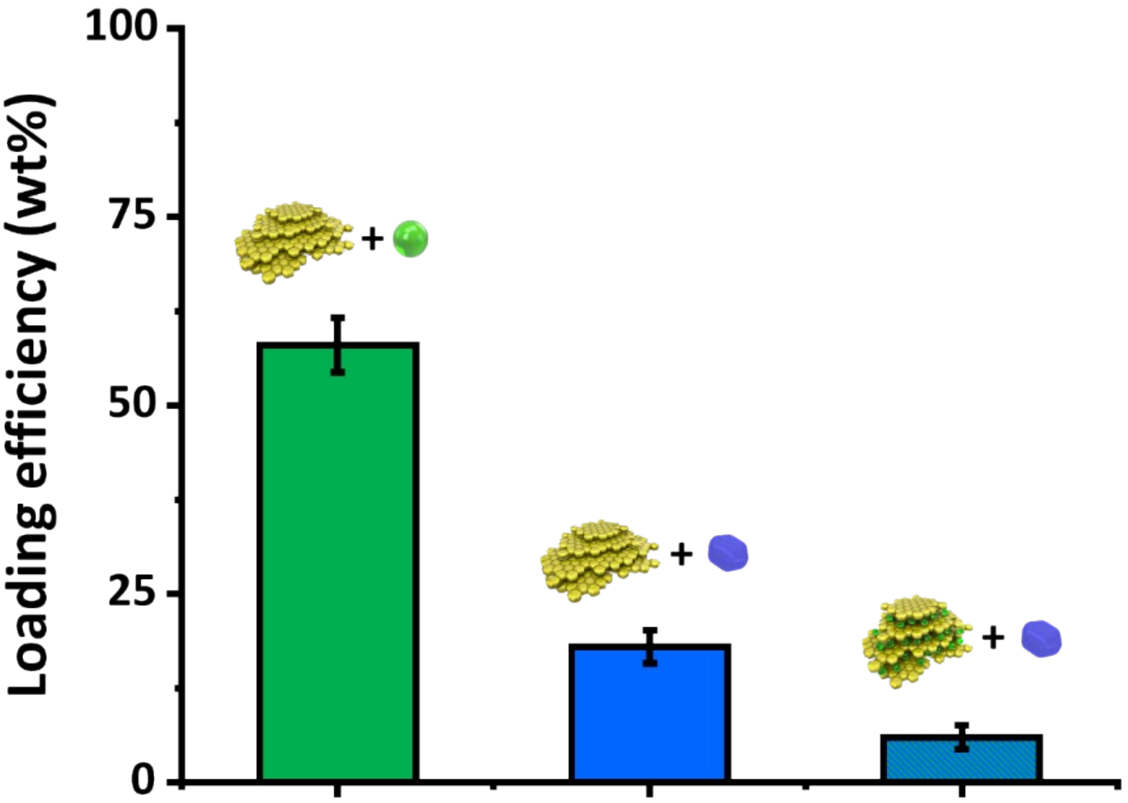

Figure S32 Loading efficiency (wt%) of TTA-DFP-nCOF when incubated with insulin (green), glucose (blue), and successively insulin followed by glucose (dashed blue-green) at pH 7.4 in 100 mM HEPES.

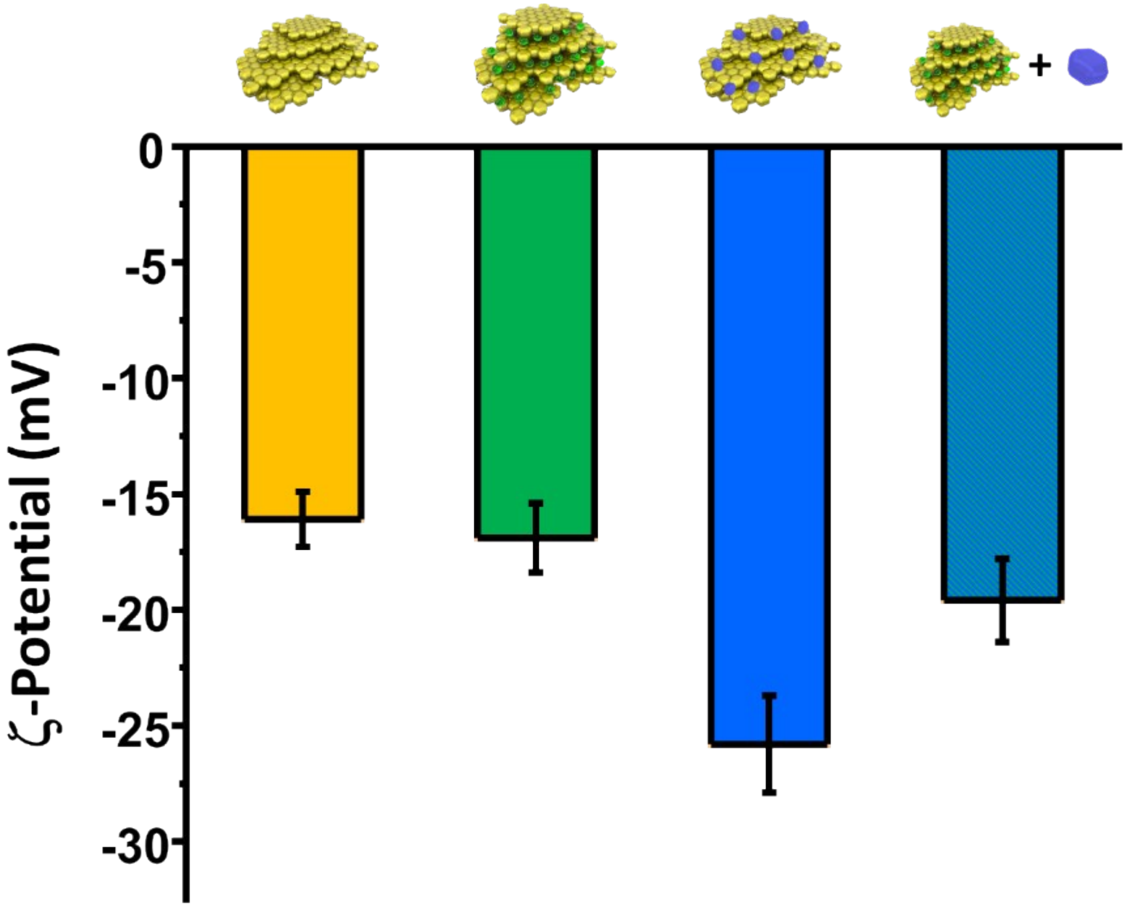

Figure S33. Zeta(ζ)-potential of TTA-DFP-nCOF (yellow), TTA-DFP-nCOF/insulin (green), TTA-DFP-nCOF/glucose (blue) and TTA-DFP-nCOF/insulin+glucose at pH 7.4 in 100 mM HEPES.

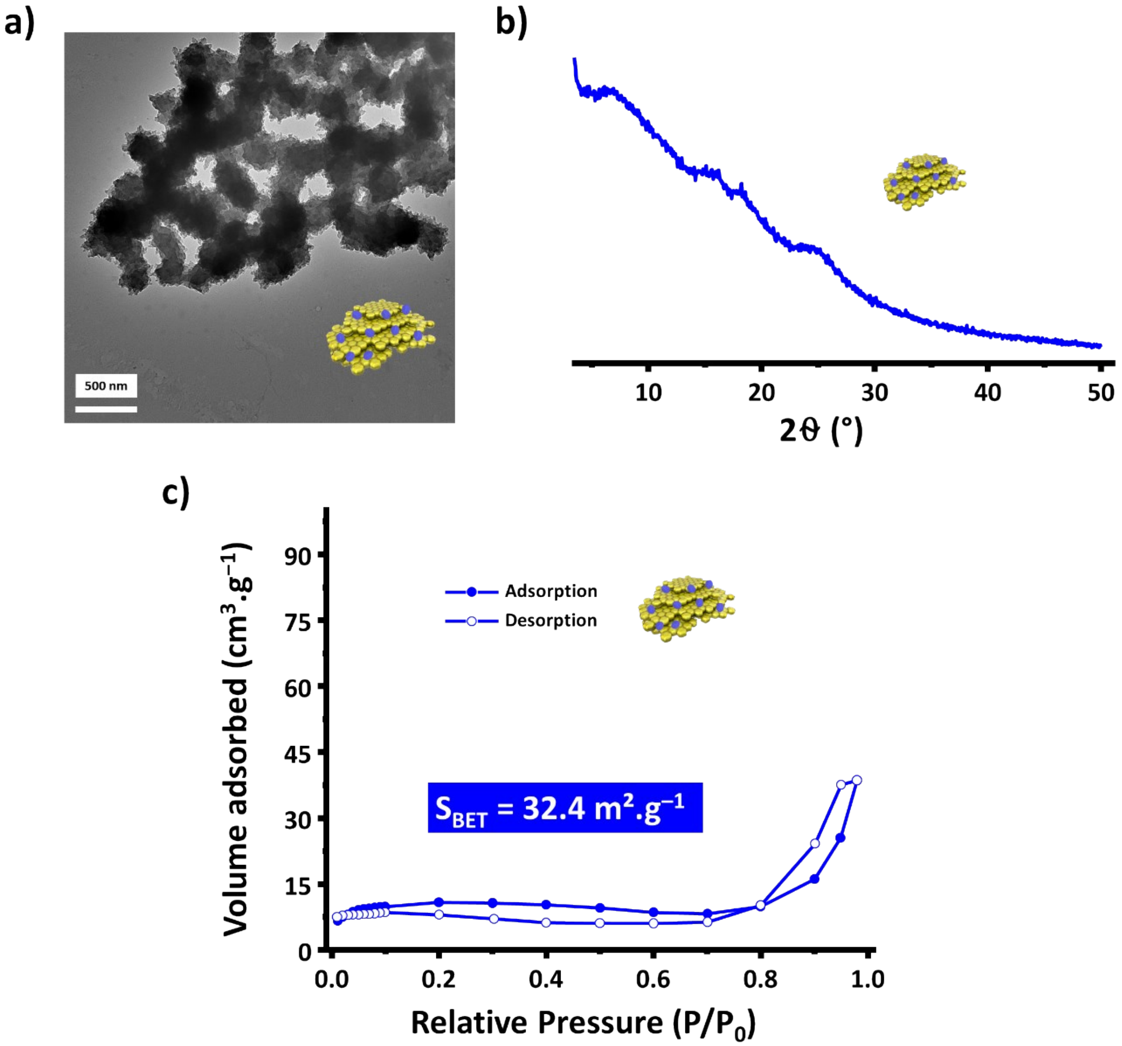

Figure S34. a) TEM image, b) PXRD pattern and c) nitrogen adsorption/desorption isotherms of TTA-DFP-nCOF loaded with glucose.

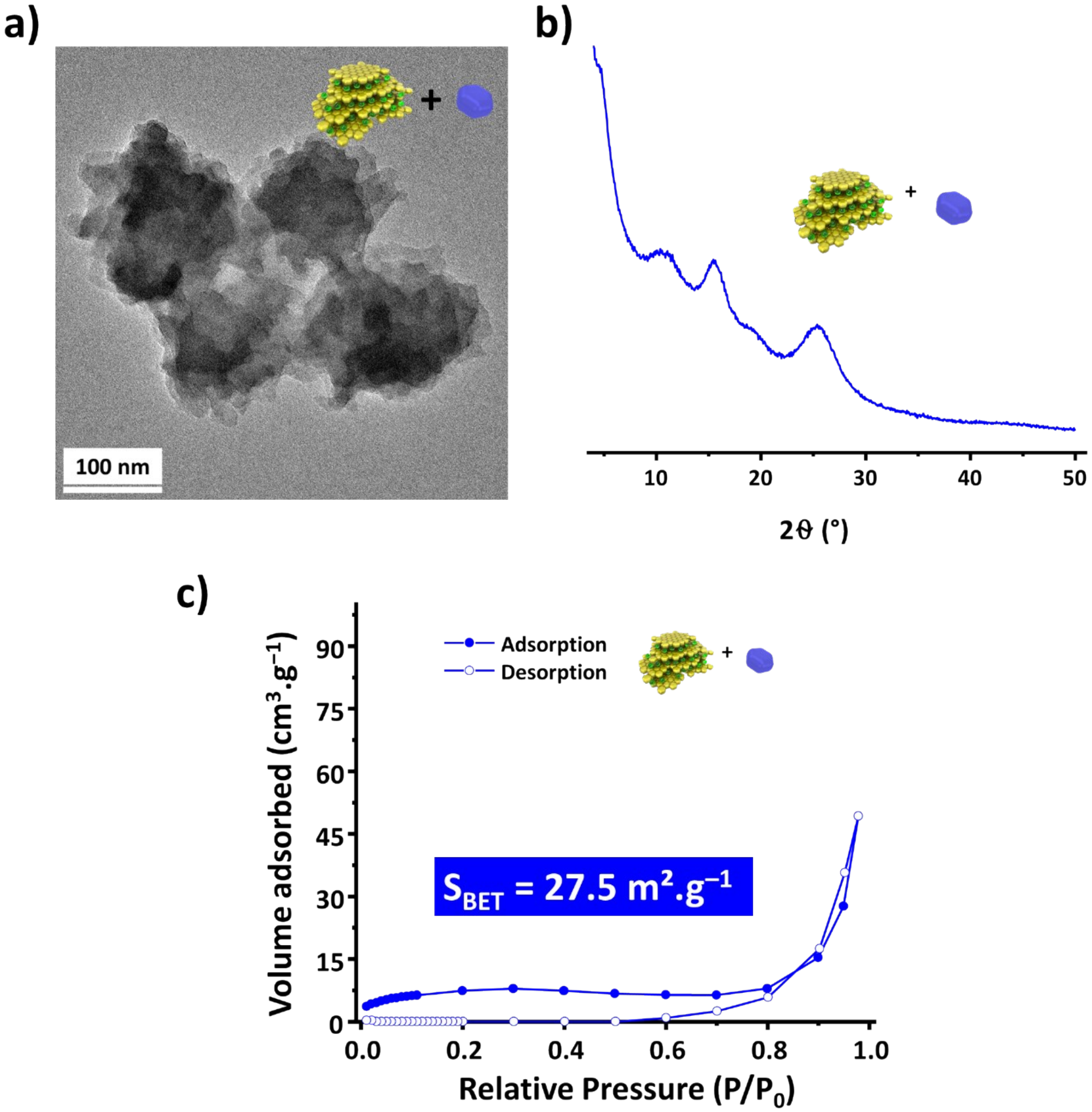

Figure S35. a) TEM image, b) PXRD pattern and c) nitrogen adsorption/desorption isotherms of TTA-DFP-nCOF/insulin (green) after release in hyperglycemic conditions ([glucose] = 5 mg.mL^{-1}).

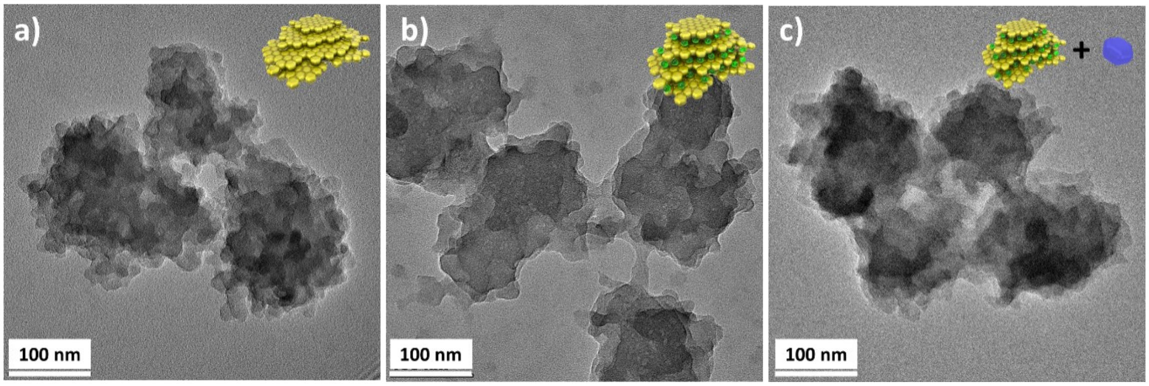

Figure S36. a) TEM images of a) TTA-DFP-nCOF, b) TTA-DFP-nCOF/insulin and c) TTA-DFP-nCOF/insulin after release in hyperglycemic conditions.

5. *In vitro* biological studies

5.1. Cell culture

Hepatocellular carcinoma (Hep-G2, ATCC HB-8065), colorectal carcinoma (HCT-116, ATCC CCL-247), colon carcinoma (RKO, ATCC CRL-2577), cervical adenocarcinoma (Hela, ATCC CCL-2), breast adenocarcinoma (MCF-7, ATCC HTB-22), metastatic breast adenocarcinoma (MDAMB-231, ATCC HTB. 26), epithelial embryonic kidney (HEK293-T, ATCC CRL-3216) and malignant glioblastoma (U251-MG, ATCC 09063001) human cell lines were cultured in Dulbecco's Modified Eagle's medium (DMEM) supplemented with 10 % fetal bovine serum (FBS), 1 % penicillin/streptomycin and 20 mL L-glutamine at 5 % CO_2 and 37 °C.
Ovarian cancer (A2780, ECACC 93112519) and intestine ileocecal adenocarcinoma (HCT-8 ATCC CCL-244) human cell lines were cultured at 5 % CO_2 and 37 °C in Roswell Park Memorial Institute (RPMI)-1640 medium complemented with 10 % fetal bovine serum (FBS) and 1% penicillin/streptomycin.

5.2. *In vitro* cell viability

Cell viability was assessed using CellTiter-Blue® Cell Viability assay (CTB, Promega). The assay measures the metabolic reduction of a non-fluorescent compound, resazurin, into a fluorescent product, resofurin, in living cells. As non-viable cells rapidly lose their metabolic activity, the

amount of the resofurin product can be used to estimate the number of viable cells following treatment. Once produced, resofurin is released from living cells into the surrounding medium. Thus, the fluorescence intensity of the medium is proportional to the number of viable cells present.

96-well plates were seeded with Hep-G2cells (~5,000 cells per well in 100 µL of DMEM) and incubated at 37 °C for 24 hours. The medium was removed and replaced with fresh medium (control) or various concentrations of test compounds and incubated at 37 °C for 48 hours. Thereafter, cells were incubated with 80 µL DMEM and 20 µL of CTB per well for 6 hours at 37 °C. The fluorescence of the resofurin product ($\lambda_{ex/em}$ 560/620) was measured. Untreated wells were used as control.

The percentage of cell viability were calculated using the following formula:

$$\text{Viability (\%)} = [(F_{treated} - F_{blank}) / (F_{control} - F_{blank})] \times 100$$

All assays were conducted in triplicate and the mean IC_{50} ± standard deviation was determined.

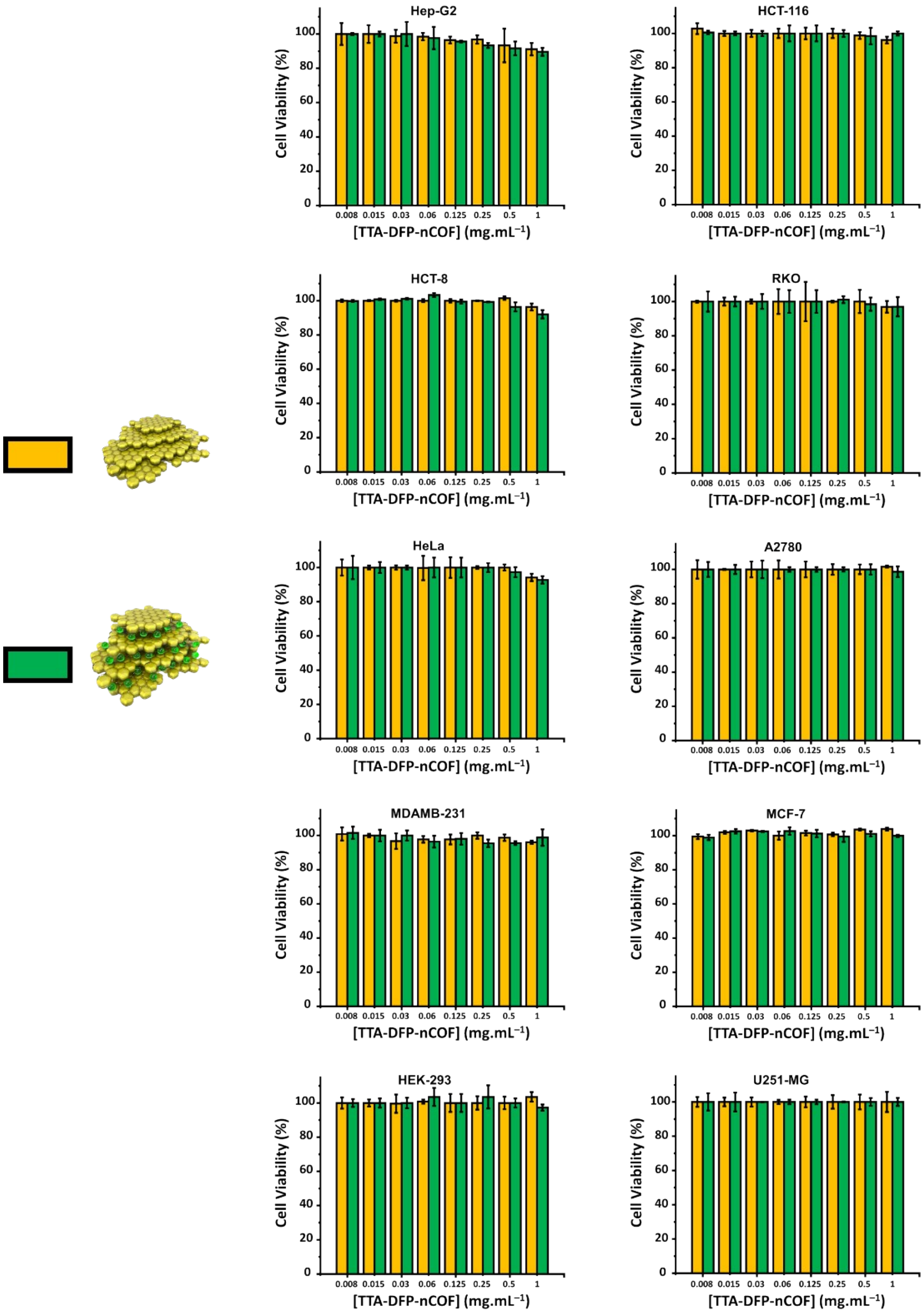

Figure S37. Viability of Hep-G2, HCT-116, HCT-8, RKO, HeLa, A2780, MDAMB-231, MCF-7, HEK-293 and U251-MG cells after 48 h incubation with TTA-DFP-nCOF (yellow) or TTA-DFP-nCOF/insulin (green) up to [TTA-DFP-nCOF] = 1 mg.mL^{-1}. Error bars represent standard deviations of triplicate measurements.

5.3. Intracellular distribution of nanoparticles using TEM analysis

TEM was used to investigate the fate of TTA-DFP-nCOF/insulin in cells, their impact on cellular structures and their interactions with organelles on 2 colon cell lines (RKO and HCT-116, 4 hours incubation times) and analyzed 4 h, 24 h and 48 h post-treatment.

For TEM analysis, cells were seeded in T75 flasks in complete DMEM and incubated 4 hours with cell-medium alone (control), TTA-DFP-nCOF/insulin ([TTA-DFP-nCOF] = 50 µg.mL^{-1}in DMEM). Cells were harvested 4, 24 and 48 hour post-treatment. Cell pellets were washed twice with phosphate-buffered saline (PBS). The cells were cryo-fixed within a few milliseconds at a pressure of 2000 bar under liquid nitrogen using a high-pressure freezer (Leica Microsystems, Germany). After freezing, the sample pod was released automatically into a liquid nitrogen bath. While still in liquid nitrogen, the sample carrier was separated from the specimen pod using precooled fine-tipped tweezers and transferred to the cryo-transfer storage box for the flat specimen carrier, where the samples were stored in preparation for freeze substitution. Freeze substitution was performed using an automatic freeze substitution (AFS) unit (Leica EM AFS2, Heerbrugg, Switzerland) in a 10 mL solution of cold dry absolute acetone (v/v) containing 1 % osmium tetroxide (w/v), 0.5 % uranyl acetate (w/v) and 5 % distilled water (v/v). The AFS unit was slowly warmed from –90 °C to 0 °C (2 °C/h), with the temperature being held at both –60 °C and –30 °C for a period of 8 h. Samples were transferred to room temperature in a closed container to prevent condensation, rinsed with absolute acetone (3 × 5 minutes) and infiltrated with 30, 60 and 100 % Epon resin for 3 h each. Epon was exchanged and individual samples were embedded in 1 mL Eppendorf® lids for 24 h at 60˚C. Finally, the samples were sectioned with an cryo ultra-microtome (Leica UC7/FC7) at room temperature using a diamond knife, and the ultrathin sections were examined under TEM (Talos F200X STEM).

The TEM images of untreated HCT-116 and RKO cells (Figures S39-40) showed typical morphological features of cells, including intact cellular membranes; numerous microvilli and membrane blebbing on the surface of plasma membranes; a well-developed rough endoplasmic reticulum, a large Golgi apparatus and other organelles or structures, such as mitochondria, small

vacuoles and granules; centric large nuclei with obvious nucleoli, surrounded by intact nuclear membranes; and, a normal distribution of heterochromatin.

All TTA-DFP-nCOF/insulin treated samples showed regular ultrastructure of the RKO and HCT-116 cells, with a roundish cellular shape and a plasma membrane rich in protrusions (such as microvilli), a well-developed rough endoplasmic reticulum, Golgi apparatus, and mitochondria, which indicate the maintenance of metabolic active cells (Figures S39-40).

After 4 h of incubation, significant amounts of TTA-DFP-nCOF/insulin can be visualized within some of the treated cells and at their surface (Figures S39-40). TTA-DFP-nCOF/insulin undergo endocytosis and are located in endosomes. Membrane deformation was also observed, confirming the internalization of TTA-DFP-nCOF/insulin by endocytosis. The TTA-DFP-nCOF/insulin which are internalized within the cell vacuoles could mainly be found as aggregates. Furthermore, we did not find any TTA-DFP-nCOF/insulin near the nucleus, as it is plausible these aggregates would be physically unable to breach the nuclear membrane pores with sizes in the range of 10–20 nm. After 24 h, TTA-DFP-nCOF/insulin could be located inside both cell lines vacuoles in the perinuclear region but no more on the membrane; cells continue growing with cells dividing (Figures S39-40). After 48 h, some HCT-116 cells still contain nanoparticles inside vacuole in the cytoplasm but most cells do not; cells continued growing and multiplying (Figure S39). In RKO cells, no nanoparticles could be detected (Figure S40).

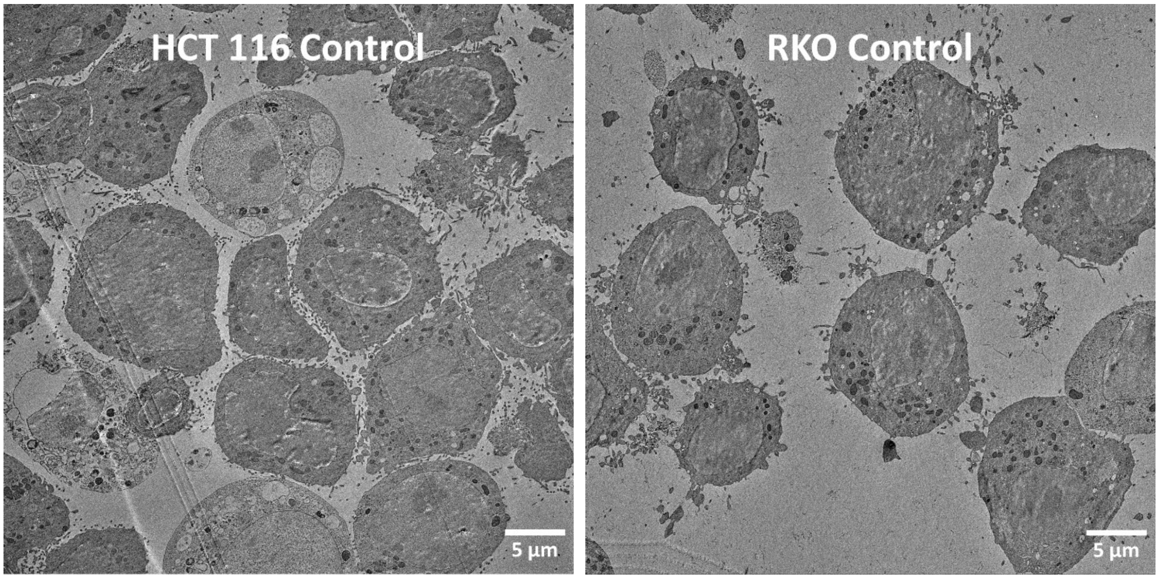

Figure S38. HCT-116 and RKO cells visualized by TEM (control cells).

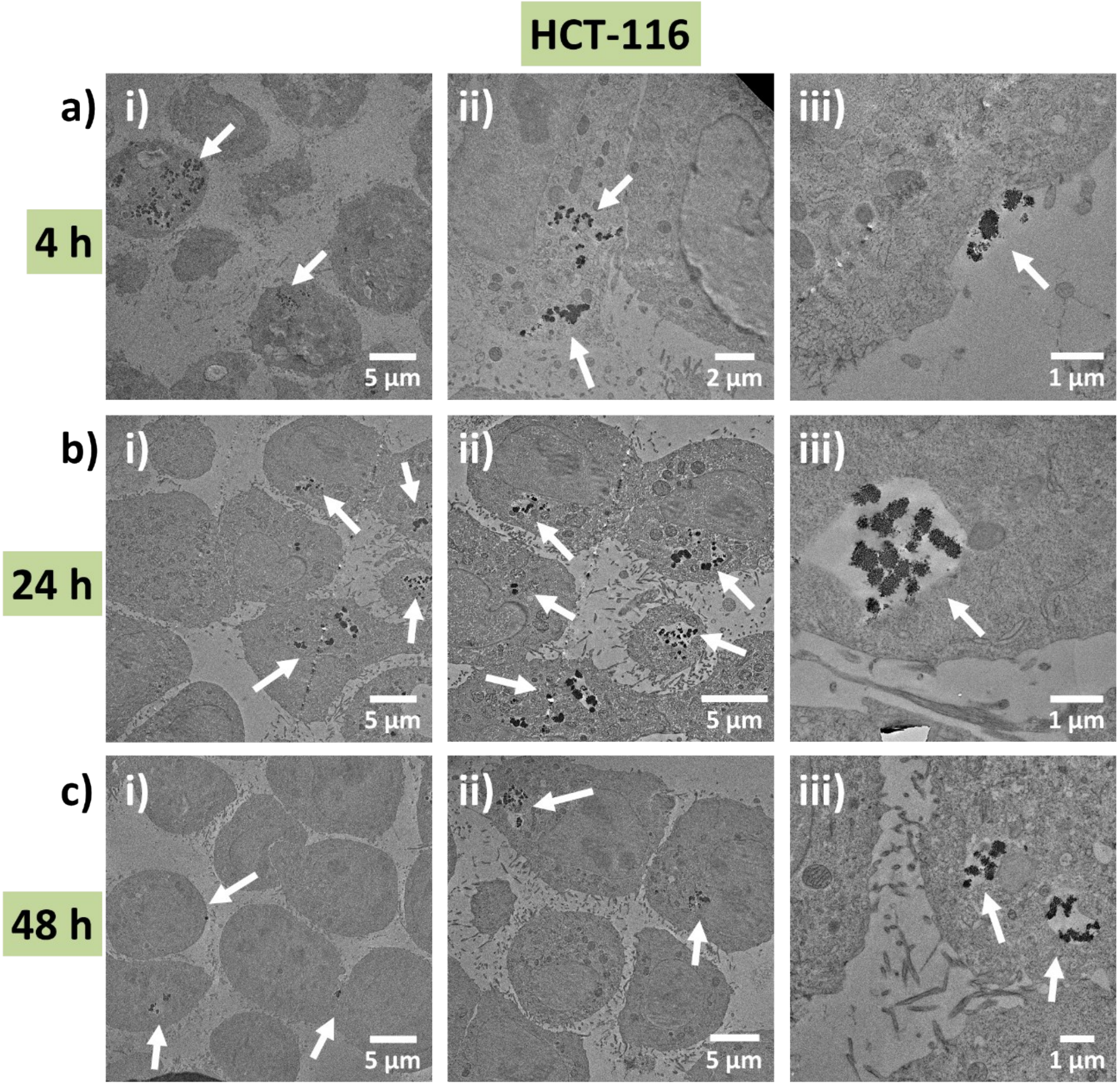

Figure S39. TEM images of HCT-116 cells treated with TTA-DFP-nCOF/insulin for 4 h at a) t = 4 h, b) t = 24 h and c) t = 48 h at various magnifications. White arrows show nanoparticle uptake in cells through endocytosis and their transit in the cytoplasm.

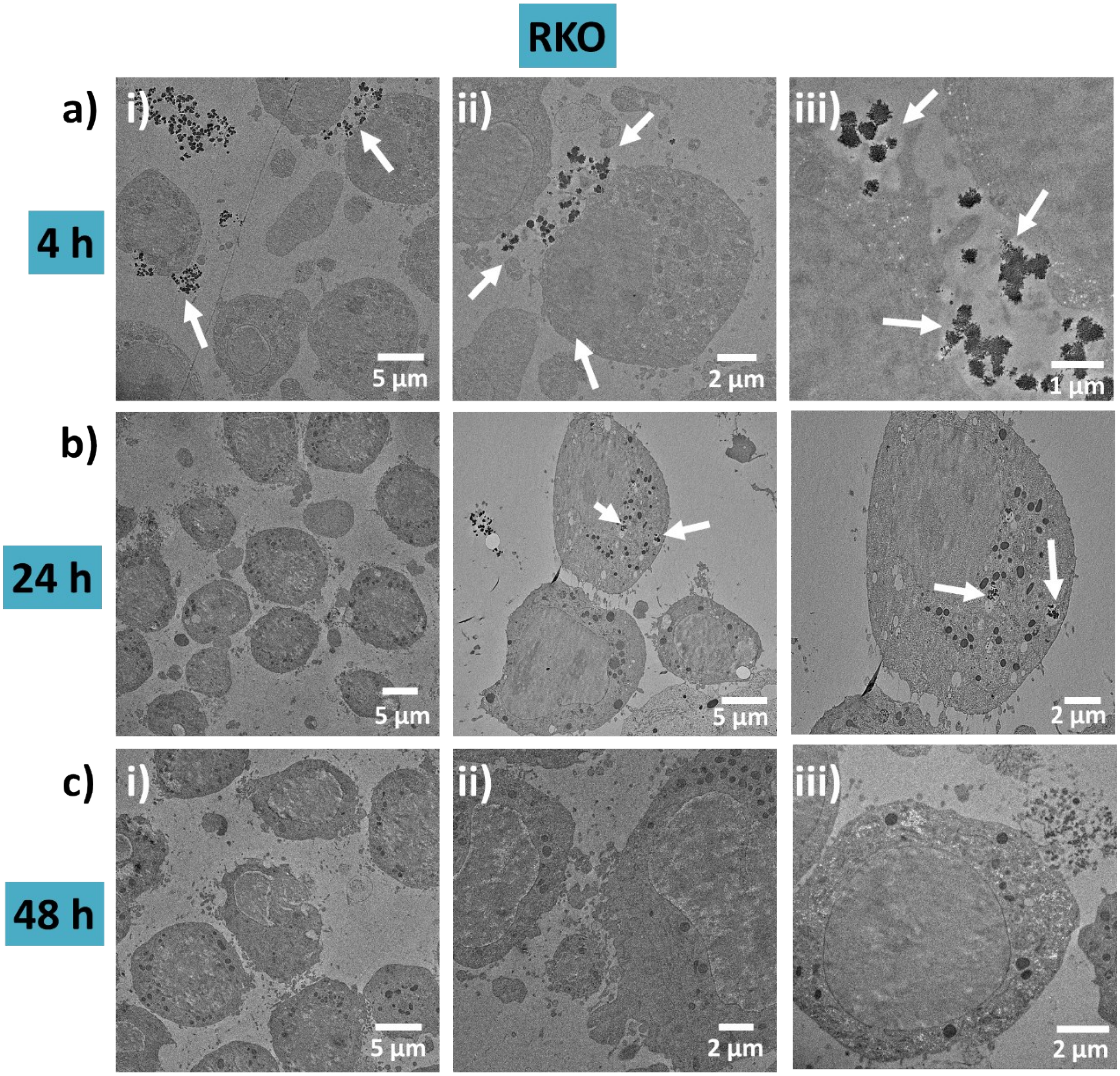

Figure S40. TEM images of RKO cells treated with TTA-DFP-nCOF/insulin for 4 h at a) t = 4 h, b) t = 24 h and c) t = 48 h at various magnifications. White arrows show nanoparticle uptake in cells through endocytosis and their transit in the cytoplasm.

5.4. Hemolysis assay

When the external membrane of the erythrocytes is destroyed, hemoglobin is released.[35-37] It is possible to estimate the amount of destroyed erythrocytes in a given test by measuring the quantity of hemoglobin in a sample by spectrophotometry.[38] Human blood was obtained from 3 healthy donors. 2.0 mL of an ethylenediaminetetraacetate-stabilized blood sample was added into 4 mL of physiological saline and then red blood cells were isolated by centrifugation (3000

rpm, 8 min). The red blood cells were washed five times with physiological saline buffer (PBS) and diluted into 2 % red blood cell suspension. TTA-DFP-nCOF or TTA-DFP-nCOF/insulin (0.75, 1.5 and 3.0 mg.mL^{-1}) was added into the red blood cell suspensions at the predetermined concentration and mixed using a gentle vortex. Meanwhile, physiological saline with or without Triton X-100 (0.3 %) was added into the red blood cell suspensions as negative and positive controls, respectively. Samples were placed in a static condition at 37 °C for 1 h. Finally, all samples were centrifuged at 5000 rpm and 100 µL of the supernatant was placed into a 96-well plate for detection at the wavelength of 540 nm. The hemolysis ratio (HR) represents the degree of red blood cell membranes destroyed in the samples.

$$\textbf{HR (\%)} = \frac{A_{sample} - A_{negative\ control}}{A_{positive\ control} - A_{negative\ control}} \times 100$$

A_{sample}, $A_{positive\ control}$, and $A_{negative\ control}$ represented the absorbance of the sample, the positive control, and the negative control, respectively. These tests were performed in triplicate.

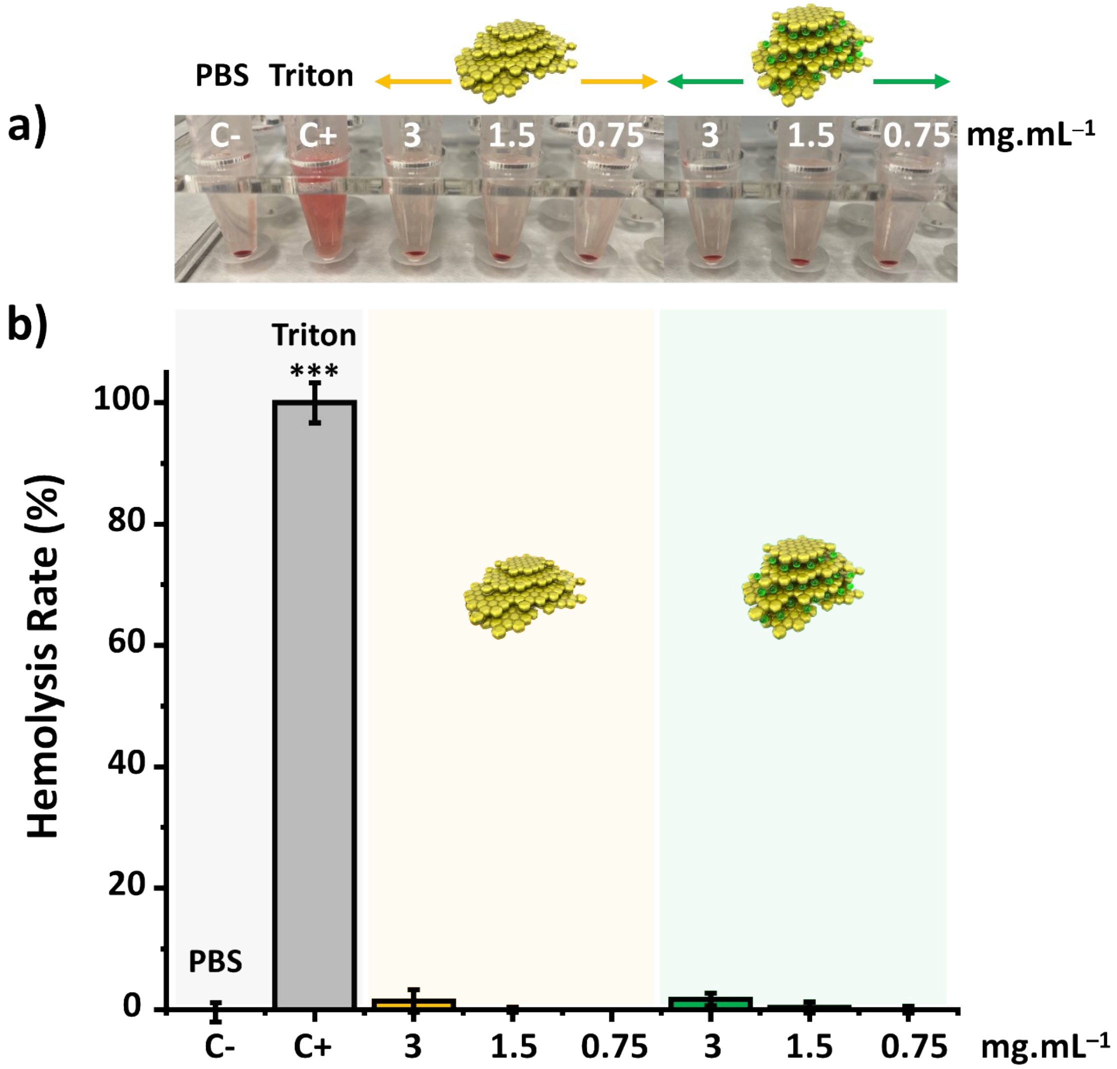

Figure S41. Hemolysis activity of TTA-DFP-nCOF and TTA-DFP-nCOF/insulin. a) Photograph after centrifugation of fresh human blood incubated with different concentrations of TTA-DFP-nCOF and TTA-DFP-nCOF/insulin up to 3 $mg.mL^{-1}$ for 1 hour. b) Hemolysis rates (%) induced by different concentrations of TTA-DFP-nCOF and TTA-DFP-nCOF/insulin up to 3 $mg.mL^{-1}$. Physiological saline in the absence or the presence of Triton X-100 (0.3 %) were respectively used as negative (C-) and positive (C+) controls. ***$p< 0.001$, significantly different from negative control. Error bars represent standard deviations of triplicate measurements.

6. *Ex Vivo* permeation study across mouse intestinal sac

Ex vivo absorption evaluation was carried out by permeation measurements in excised rat small intestine as described elsewhere.[39, 40] Mice (25 g) were anaesthetized with isoflurane, killed and exsanguinated. As the animal was placed under anesthesia and euthanized for a different purpose, no ethical approval was necessary for retrieval of the tissue. Freshly excised intestinal tissues were washed with PBS and cut into pieces of 5–4 cm. 0.3 mL of TTA-DFP-nCOF/insulin-FITC (1 $mg.mL^{-1}$) was syringed into intestinal sacs; the filled tissues were incubated in oxygenated tissue culture Dulbecco's Modified Eagle's Medium (DMEM, 10 mL) at 37 °C. Sample solution (0.1 mL) was withdrawn from the serosal side at fixed time intervals up to 180 min and replaced with fresh medium. Fluorescence signal for FITC was measured on a fluorescent plate reader, (FITC excitation/emission: 495 nm/519 nm) and compared to a standard curve of log dilutions for TTA-DFP-nCOF/insulin-FITC ranging from 1 to 1×10^{-6} M. Tests were carried out in triplicate on three different intestinal segments from three different mice.

$$\textbf{Apparent permeability } (\mu g.cm^{-2}) = \frac{\textbf{Concentration} \times \textbf{volume}}{\textbf{Mucosal surface area}}$$

To calculate mucosal surface area, the intestine was considered as a cylinder and the following equation was used:

$$\textbf{Mucosal surface area } (cm^{-2}) = 2\pi r(h + r)$$

where *h* = length and *r* = radius of intestinal sac

At the end of the experiments, tissues were washed with normal saline, and drug accumulation in the gut wall was investigated by TEM of the serosal medium as well the intestinal tissue.

Intestinal tissues were fixed in 4.5% paraformaldehyde solution. The fixed samples were cryo-fixed within a few milliseconds at a pressure of 2000 bar under liquid nitrogen using a high-pressure freezer (Leica Microsystems, Germany). Freeze substitution was performed using an automatic freeze substitution (AFS) unit (Leica EM AFS2, Heerbrugg, Switzerland) in a 10 mL solution of cold, dry, absolute acetone (v/v) containing 1 % osmium tetroxide (w/v), 0.5 % uranyl acetate (w/v), 5 % distilled water (v/v) and embedded with epoxy resin. Subsequently, an

ultrathin intestinal specimen was made and observed with high-resolution TEM (Talos F200X STEM).

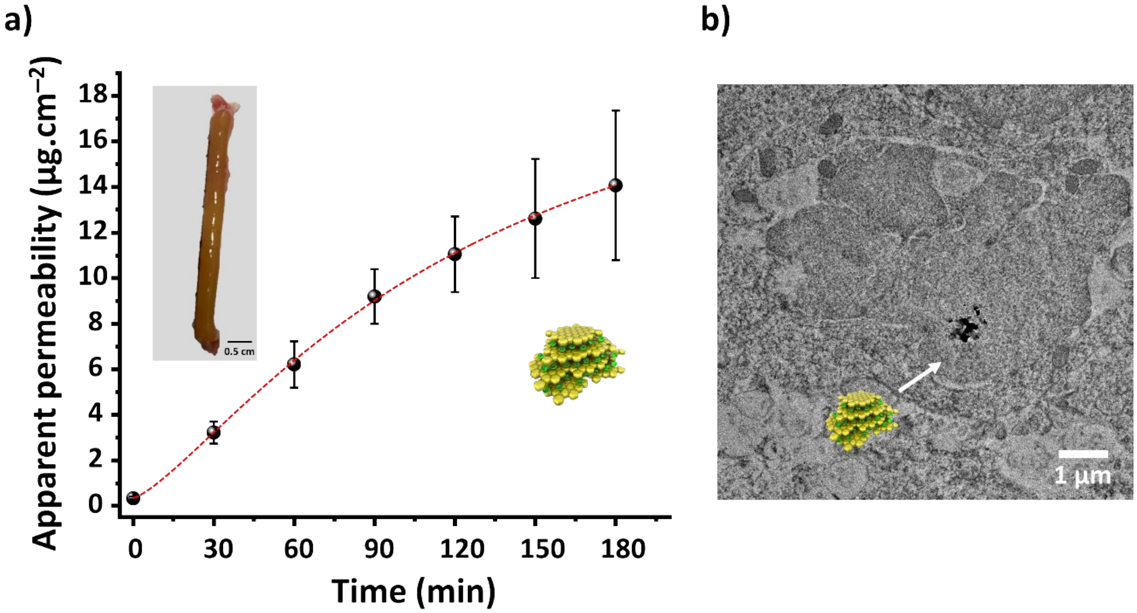

Figure S42. *Ex vivo* permeation studies of TTA-DFP-nCOF/insulin-FITC. a) Apparent permeability profile of TTA-DFP-nCOF/insulin-FITC across mouse intestinal tissue in DMEM at 37 °C. The formulations under study were syringed into intestinal sacs obtained from freshly excised mouse tissue. The filled tissues were incubated in oxygenated buffer at 37 °C. Sample solution was withdrawn at fixed time intervals up to 180 min and replaced with fresh medium. Data are shown as the mean. Inset: intestinal sac containing 300 μL of TTA-DFP-nCOF/insulin-FITC (1 mg.mL^{-1}). Tests were carried out in triplicate on three different intestinal segments from three different mice. b) TEM micrograph of *ex vivo* intestinal tissue after 180 min of TTA-DFP-nCOF/insulin-FITC treatment showing the presence of TTA-DFP-nCOF/insulin (white arrows).

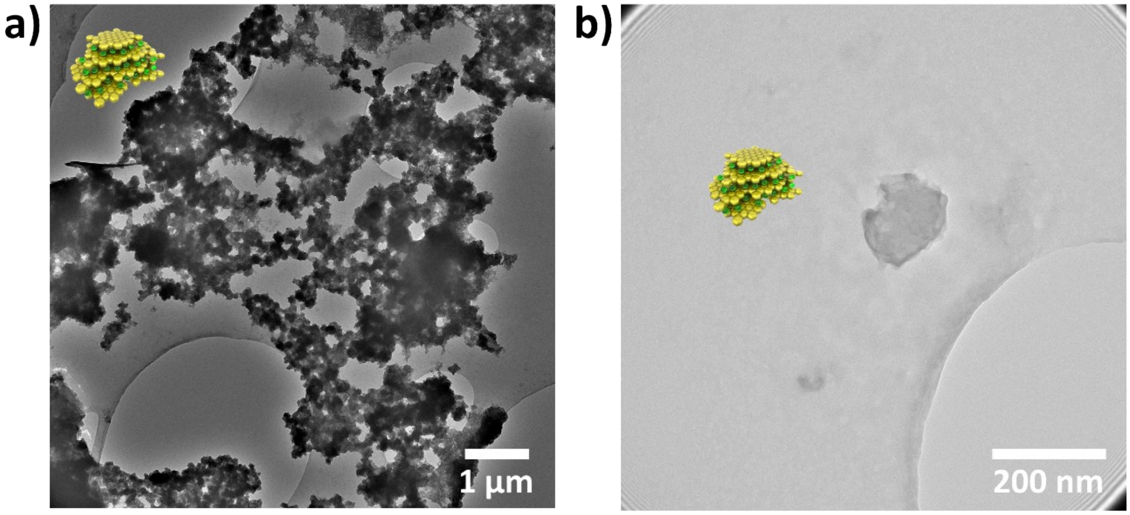

Figure S43. TEM images of the serosal medium of *ex vivo* permeation studies showing that TTA-DFP-nCOF can cross intact the intestinal barrier without change in morphology or size.

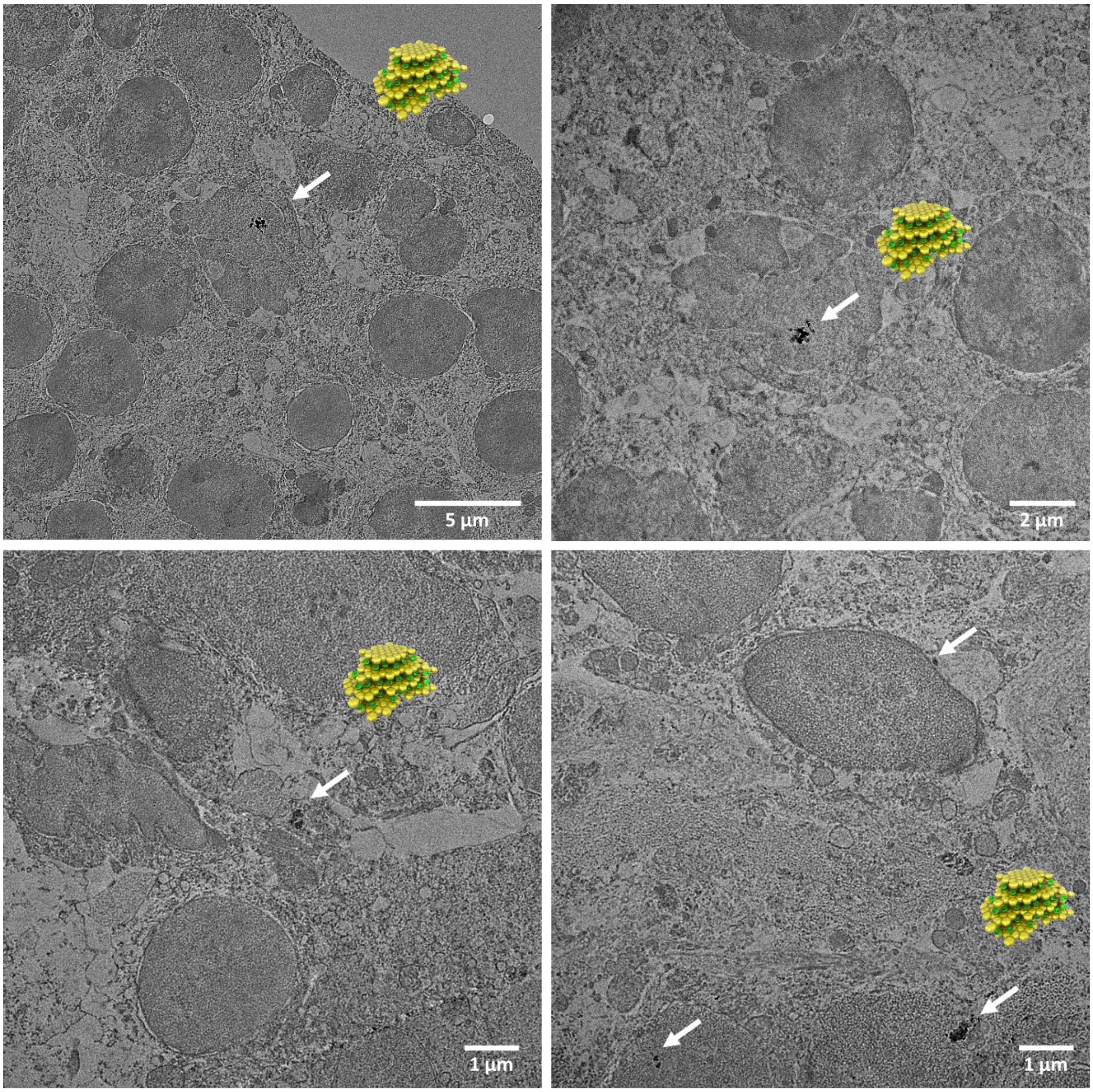

Figure S44. TEM images of *ex vivo* intestinal tissues after 180 min of TTA-DFP-nCOF/insulin-FITC treatment showing the distribution of TTA-DFP-nCOF/insulin through the intestine (white arrows).

7. *In vivo* animal assessments

All animals were raised in accordance with the policies of the University of Tlemcen Institutional Animal Care and Use Committee (IACUC) (accreditation number: D01N01UN130120150006).

7.1. Animal

Wistar rats (12 weeks, 200 g ±20) were used for this study. They were obtained from Pasteur Institute (Algiers, Algeria). Rats were housed individually in wood-chip bedded plastic cages at constant temperature (25 °C), maintained on a 12:12 hours light/dark cycle and fed with a standard pellet diet and water *ad libitum*. The study was conducted in accordance with the national guidelines for the care and use of laboratory animals.

7.2. T1D Induction

Type 1 Diabetes was induced via a single intraperitoneal injection of streptozotocin (STZ, dissolved in 10 mM citrate buffer at pH 4.5) at the STZ dose of 45 $mg.kg^{-1}$ body weight. Rats were returned to their cages, and given food and water for the next 4 days till the induction of diabetes. The blood glucose level was monitored using a blood glucose monitoring system (AccuChek Performa, Hoffman-La Roche) by taking samples from a rat tail vein. The rats showing fasting blood glucose level ≥ 250 mg/dL (13.7 $mmol.L^{-1}$) were considered as diabetic and were selected for the studies. The rats were fasted overnight and remained fasted during the period of experiment, but were allowed to drink water.

7.3. *In vivo* hypoglycemic effect

Rats were randomly divided into six groups (n = 3), the formulations administered to the T1D rats were as follows: 1) TTA-DFP-nCOF/insulin administered by oral gavage (o.g., 50 $IU.kg^{-1}$); 2) TTA-DFP-nCOF administered by oral gavage (o.g., 2 $mg.kg^{-1}$); 3) insulin solution administered by oral gavage (o.g., 50 $IU.kg^{-1}$); 4) insulin solution administered subcutaneously (5 $IU.kg^{-1}$) set as the positive control with 100 % pharmacological availability of insulin; 4) untreated diabetic rats and 6) non-diabetic rats. Blood glucose level was determined with a glucometer. Blood samples were taken from the tail veins every hour for 10 hours.

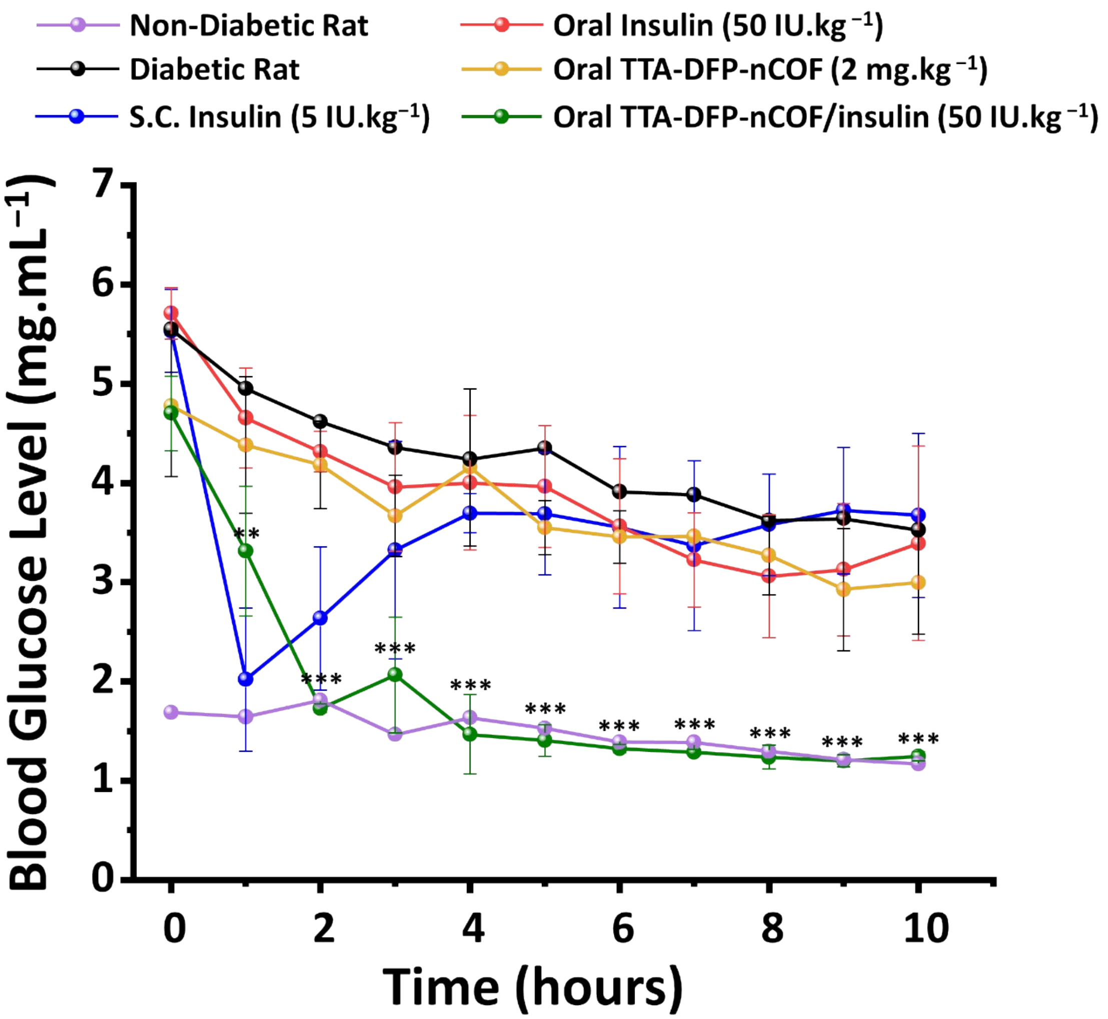

Figure S45. TTA-DFP-nCOF/insulin regulate glucose uptake *in vitro* and *in vivo*. a) *In vivo* blood glucose level (of initial %) changes versus time curves of the STZ-induced diabetic rats after oral administration of TTA-DFP-nCOF/insulin (green) and free-form insulin solution (red), all at an insulin dosage of 50 IU.kg^{-1}. The group by subcutaneous injection (S.C., blue) of insulin at 5 IU.kg^{-1} was set as a positive control, while the group orally administrated with empty TTA-DFP-nCOF (yellow) at 2 mg.kg^{-1} served as a negative control. Blood glucose level of diabetic (black) and non-diabetic (purple) rats are also shown. TTA-DFP-nCOF/insulin showed statistically significant differences in hypoglycemic effect compared with diabetic control (*p<0.05; **p<0.01; ***p<0.001). Each value represents mean ±S.D. (n=3).

7.4. *In vivo* plasma insulin level

Rats were randomly divided into 3 groups (n = 3), the formulations administered to the T1D rats were as follows: 1) TTA-DFP-nCOF/insulin administered by oral gavage (o.g., 50 IU.kg^{-1}); 2) insulin solution administered subcutaneously (SC, 5 IU.kg^{-1}) set as the positive control with 100 % pharmacological availability of insulin and 3) untreated diabetic rats. Pharmacological effect was determined by measuring the increase of serum insulin in diabetic rats during 10 h. Insulin plasma level was evaluated using a Rat Ins1 Insulin ELISA Kit from Sigma-Aldritch (RAB00904-1KT). Blood samples were collected from the tail veins every hour over 10 hours. The area above the curve (AAC) was calculated using the trapezoidal method. The pharmacological availability (PA) calculated as the cumulative hypoglycemic effect relative to 100 % PA of sc. free insulin was determined using the equation:

$$\textbf{Pharmacological Availability (PA)} = \frac{\textbf{AAC oral TTA-DFP-nCOF/Insulin} \times \textbf{Dose insulin sc.}}{\textbf{AAC insulin sc.} \times \textbf{Dose oral TTA-DFP-nCOF/Insulin}}$$ [19, 41]

Homeostatic model assessment (HOMA) of insulin resistance (HOMA-IR) and insulin-sensibility (HOMA-IS) of β-cell function were calculated using the following equations:

$$\textbf{HOMA-IR} = \frac{\textbf{Fasting glucose [mg.dL}^{-1}\textbf{]} \times \textbf{Fasting insulin [mU.L}^{-1}\textbf{]}}{\textbf{405}}$$

$$\textbf{HOMA-IS} = \frac{\textbf{10000}}{\textbf{Fasting glucose [mg.dL}^{-1}\textbf{]} \times \textbf{Fasting insulin [mU.L}^{-1}\textbf{]}}$$ [42]

7.5. *In vivo* oral glucose tolerance test (OGTT).

For oral glucose tolerance test (OGTT), rats were randomly divided into 3 groups (n = 3) corresponding to i) TTA-DFP-nCOF/insulin administered by oral gavage (o.g., 50 IU.kg^{-1}); ii) insulin solution administered subcutaneously (SC, 5 IU.kg^{-1}) and iii) untreated diabetic rats. Animals have first received the TTA-DFP-nCOF/insulin by oral gavage or the subcutaneous insulin injection. 3 hours after that, they received 2.5 g.kg^{-1} of glucose dissolved in 1ml of water and glycaemia was evaluated for 280 min. Glycaemia was measured at time zero (basal) and every 30 min up to 280 min after gavage of 2.5 g.kg^{-1} body weight of glucose from the tail vein.

7.6. Histopathology

The collected tissues were embedded in paraffin after fixing the tissues in 10 % formalin. Serial sections were cut and stained with hematoxylin and eosin. The sections were examined under high-power microscope (200×) and photomicrographs were taken.

SC insulin-treated rats' livers compared to the non-diabetic control displayed an increase of big hepatocytes, a necrosis of hepatocytes and a narrowing in the sinusoids (Figure 3g-ii) due to the STZ-induced diabetes.[43, 44] Histopathological study of the livers of the group treated with TTA-DFP-nCOF/insulin showed similar structures to the non-diabetic rats, with normal hepatocytes and sinusoids. Regarding the kidney function, SC insulin-treated rats displayed an increase of the size of Bowman capsules,[45] hypertrophy of the glomeruli[44] and necrosis of the tubules[46] (Figure 3g-v) also due to STZ administration to induced diabetes. The kidneys of the rats treated with TTA-DFP-nCOF/insulin showed fewer alterations than the subcutaneous insulin rat kidneys, smaller Bowman's spaces and well-individualized tubules.

7.7. Biochemical determinations

Liver function test was carried out using serum biomarkers such as aspartate amino-transferase (AST) and Alanine transaminase (ALT) measured from the plasma obtained from the tail vein using SPINREACT kit. Kidney function test was performed using as urea and creatinine measured by SPINREACT kit.

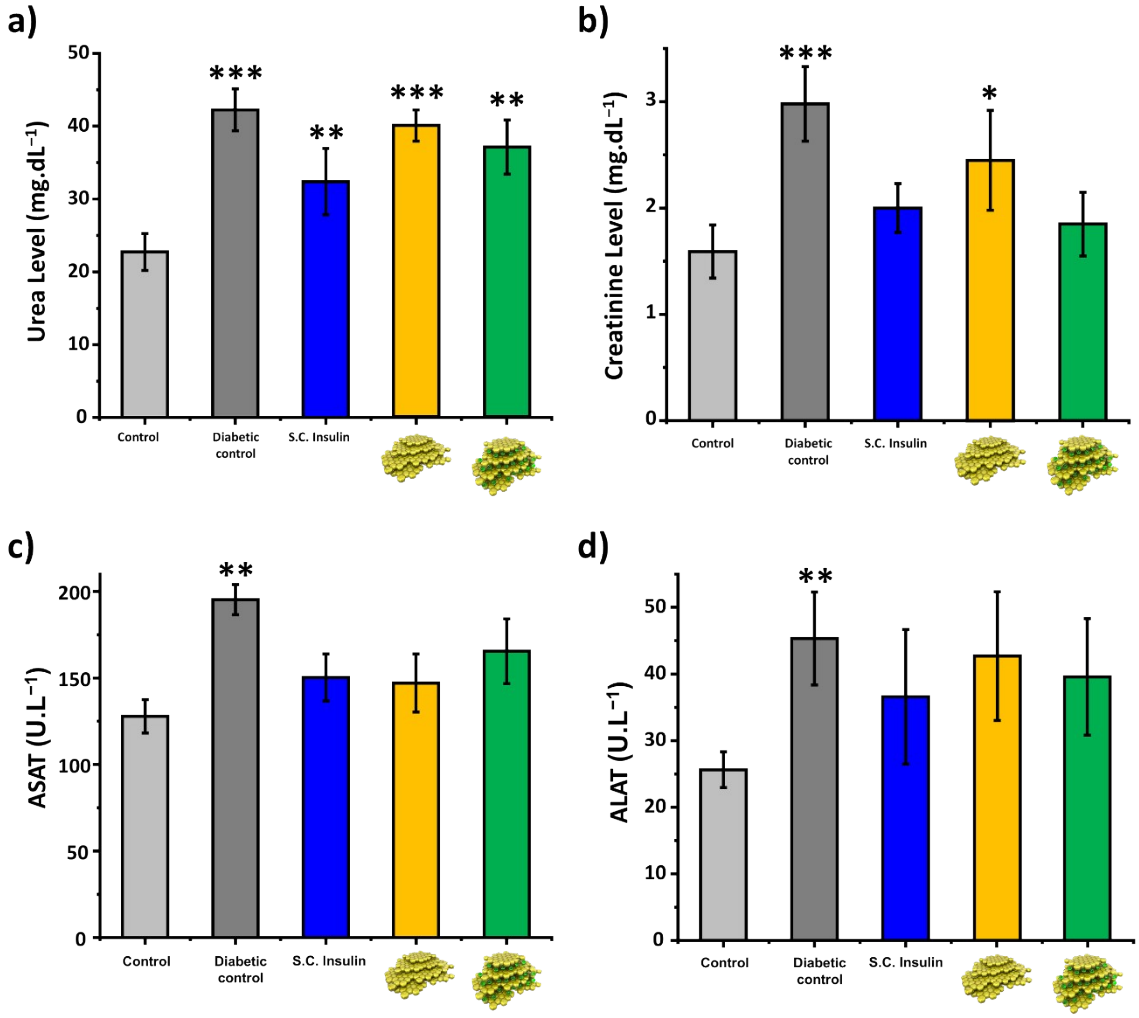

Figure S46. TTA-DFP-nCOF/insulin did not show toxicity *in vivo*. a) urea, b) creatinine levels and transaminase activities: c) aspartate aminotransferase (ASAT) and d) alanine aminotransferase (ALAT) activity of non-diabetic rats (grey) and the STZ-induced diabetic rats after oral administration of TTA-DFP-nCOF/insulin (green) at the insulin dosage of 50 $IU.kg^{-1}$. The group by subcutaneous injection (S.C., blue) of insulin at 5 $IU.kg^{-1}$ was set as a positive control, while the group orally administrated with empty TTA-DFP-nCOF (yellow) at 2 $mg.kg^{-1}$ served as a negative control. Diabetic rat levels (control, dark grey) are also shown. (*p<0.05; **p<0.01; ***p<0.001). Each value represents mean ±S.D. (n=3).

References

1. G. Bentley, E. Dodson, G. Dodson, D. Hodgkin and D. Mercola, *Nature*, 1976, **261**, 166-168.

2. A. Samui, Happy and S. K. Sahu, *Micropor. Mesopor. Mat.*, 2020, **291**, 109700.
3. M. Li, S. Qiao, Y. Zheng, Y. H. Andaloussi, X. Li, Z. Zhang, A. Li, P. Cheng, S. Ma and Y. Chen, *J. Am. Chem. Soc.*, 2020, **142**, 6675-6681.
4. G. Zhang, Y. Ji, X. Li, X. Wang, M. Song, H. Gou, S. Gao and X. Jia, *Adv. Healthc. Mater.*, **n/a**, 2000221.
5. Q. Sun, B. Aguila, P. C. Lan and S. Ma, *Adv. Mater.*, 2019, **31**, 1900008.
6. F. L. Oliveira, S. P. de Souza, J. Bassut, H. M. Álvarez, Y. Garcia-Basabe, R. O. M. Alves de Souza, P. M. Esteves and R. S. B. Gonçalves, *Chem. Eur. J.*, 2019, **25**, 15863-15870.
7. Q. Sun, C.-W. Fu, B. Aguila, J. Perman, S. Wang, H.-Y. Huang, F.-S. Xiao and S. Ma, *J. Am. Chem. Soc.*, 2018, **140**, 984-992.
8. S. Kandambeth, V. Venkatesh, D. B. Shinde, S. Kumari, A. Halder, S. Verma and R. Banerjee, *Nat. Commun.*, 2015, **6**, 6786.
9. N. Tennagels and U. Werner, *Arch. Physiol. Biochem.*, 2013, **119**, 1-14.
10. Y. Zhang, W. Wei, P. Lv, L. Wang and G. Ma, *Eur. J. Pharm. Biopharm.*, 2011, **77**, 11-19.
11. C. M. Silva, A. J. Ribeiro, I. V. Figueiredo, A. R. Gonçalves and F. Veiga, *Int. J. Pharm.*, 2006, **311**, 1-10.
12. M. Lopes, N. Shrestha, A. Correia, M.-A. Shahbazi, B. Sarmento, J. Hirvonen, F. Veiga, R. Seiça, A. Ribeiro and H. A. Santos, *J. Control. Release*, 2016, **232**, 29-41.
13. J. Sheng, L. Han, J. Qin, G. Ru, R. Li, L. Wu, D. Cui, P. Yang, Y. He and J. Wang, *ACS Appl. Mater. Interfaces*, 2015, **7**, 15430-15441.
14. R. Sheshala, K. K. Peh and Y. Darwis, *Drug. Dev. Ind. Pharm.*, 2009, **35**, 1364-1374.
15. C. J. Thompson, L. Tetley and W. P. Cheng, *Int. J. Pharm.*, 2010, **383**, 216-227.
16. S. Chen, F. Guo, T. Deng, S. Zhu, W. Liu, H. Zhong, H. Yu, R. Luo and Z. Deng, *AAPS PharmSciTech*, 2017, **18**, 1277-1287.
17. B. Sarmento, S. Martins, D. Ferreira and E. B. Souto, *Int. J. Nanomedicine*, 2007, **2**, 743-749.
18. E. Muntoni, E. Marini, N. Ahmadi, P. Milla, C. Ghè, A. Bargoni, M. T. Capucchio, E. Biasibetti and L. Battaglia, *Acta Diabetol.*, 2019, **56**, 1283-1292.
19. X. Li, J. Qi, Y. Xie, X. Zhang, S. Hu, Y. Xu, Y. Lu and W. Wu, *Int. J. Nanomedicine*, 2013, **8**, 23-32.
20. X. Zhang, J. Qi, Y. Lu, X. Hu, W. He and W. Wu, *Nanoscale Res. Lett.*, 2014, **9**, 185.
21. M. Ning, Y. Guo, H. Pan, H. Yu and Z. Gu, *Drug Deliv.*, 2005, **12**, 399-407.
22. X. Han, Y. Lu, J. Xie, E. Zhang, H. Zhu, H. Du, K. Wang, B. Song, C. Yang, Y. Shi and Z. Cao, *Nat. Nanotech.*, 2020, **15**, 605-614.
23. Z. Gu, T. T. Dang, M. Ma, B. C. Tang, H. Cheng, S. Jiang, Y. Dong, Y. Zhang and D. G. Anderson, *ACS Nano*, 2013, **7**, 6758-6766.
24. H.-J. Cho, J. Oh, M.-K. Choo, J.-I. Ha, Y. Park and H.-J. Maeng, *Int. J. Biol. Macromol.*, 2014, **63**, 15-20.
25. X. Zhao, C. Shan, Y. Zu, Y. Zhang, W. Wang, K. Wang, X. Sui and R. Li, *Int. J. Pharm.*, 2013, **454**, 278-284.
26. A. Díaz, A. David, R. Pérez, M. L. González, A. Báez, S. E. Wark, P. Zhang, A. Clearfield and J. L. Colón, *Biomacromolecules*, 2010, **11**, 2465-2470.
27. Y. Zhou, L. Liu, Y. Cao, S. Yu, C. He and X. Chen, *ACS Appl. Mater. Interfaces*, 2020, **12**, 22581-22592.
28. Y. Duan, F. Ye, Y. Huang, Y. Qin, C. He and S. Zhao, *Chem. Commun.*, 2018, **54**, 5377-5380.
29. Y. Chen, P. Li, J. A. Modica, R. J. Drout and O. K. Farha, *J. Am. Chem. Soc.*, 2018, **140**, 5678-5681.
30. S. Wang, Y. Chen, S. Wang, P. Li, C. A. Mirkin and O. K. Farha, *J. Am. Chem. Soc.*, 2019, **141**, 2215-2219.
31. T. Prakasam, M. Lusi, M. Elhabiri, C. Platas-Iglesias, J.-C. Olsen, Z. Asfari, S. Cianférani-Sanglier, F. Debaene, L. J. Charbonnière and A. Trabolsi, *Angew. Chem. Int. Ed. Engl.*, 2013, **52**, 9956-9960.

32. A. Halder, S. Kandambeth, B. P. Biswal, G. Kaur, N. C. Roy, M. Addicoat, J. K. Salunke, S. Banerjee, K. Vanka, T. Heine, S. Verma and R. Banerjee, *Angew. Chem. Int. Ed. Engl.*, 2016, **55**, 7806-7810.
33. A. Manciulea, A. Baker and J. R. Lead, *Chemosphere*, 2009, **76**, 1023-1027.
34. F. Benyettou, H. Fahs, R. Elkharrag, R. A. Bilbeisi, B. Asma, R. Rezgui, L. Motte, M. Magzoub, J. Brandel, J. C. Olsen, F. Piano, K. C. Gunsalus, C. Platas-Iglesias and A. Trabolsi, *RSC Adv.*, 2017, **7**, 23827-23834.
35. M. A. Dobrovolskaia, P. Aggarwal, J. B. Hall and S. E. McNeil, *Mol. Pharm.*, 2008, **5**, 487-495.
36. M. A. Dobrovolskaia and S. E. McNeil, *J. Control. Release*, 2013, **172**, 456-466.
37. D. Morera and S. A. MacKenzie, *Vet. Res.*, 2011, **42**, 89-89.
38. B. I. Macías-Martínez, D. A. Cortés-Hernández, A. Zugasti-Cruz, B. R. Cruz-Ortíz and E. M. Múzquiz-Ramos, *J. Appl. Res. Technol.*, 2016, **14**, 239-244.
39. M. J. Ansari, M. K. Anwer, S. Jamil, R. Al-Shdefat, B. E. Ali, M. M. Ahmad and M. N. Ansari, *Drug Deliv.*, 2016, **23**, 1972-1979.
40. S. W. Mateer, J. Cardona, E. Marks, B. J. Goggin, S. Hua and S. Keely, *J. Vis. Exp.*, 2016, DOI: 10.3791/53250, e53250-e53250.
41. N. Reix, A. Parat, E. Seyfritz, N. Ebel, L. Danicher, M. Pinget, Y. Frère, N. Jeandidier and S. Sigrist, *Diabetes Metab.*, 2010, **36**, A77.
42. A.-B. M. Aref, O. M. Ahmed, L. A. Ali and M. Semmler, *J. Diabetes Res.*, 2013, **2013**, 429154.
43. Y. Wang-Fischer and T. Garyantes, *J. Diabetes Res.*, 2018, **2018**, 8054073-8054073.
44. O. Ozdemır, P. P. Akalın, N. Baspınar and F. Hatıpoglu, *Bull. Vet. Inst. Pulawy*, 2009, **53**, 783-790.
45. X. Yang, *Drug Deliv.*, 2019, **26**, 849-859.
46. O. M. Ahmed, T. M. Ali, M. A. Abdel Gaid and A. A. Elberry, *PloS one*, 2019, **14**, e0214349.
